# Chemotherapy induces feedback up-regulation of CD44v6 in colorectal cancer initiating cells through *β*-catenin/MDR1 signaling to sustain chemoresistance

**DOI:** 10.3389/fonc.2022.906260

**Published:** 2022-10-18

**Authors:** Shibnath Ghatak, Vincent C. Hascall, Nikos Karamanos, Roger R. Markwald, Suniti Misra

**Affiliations:** ^1^ Department of Regenerative Medicine and Cell Biology, Medical University of South Carolina, Charleston, SC, United States; ^2^ Department Natural Sciences, Trident Technical College, North Charleston, SC, United States; ^3^ Department of Biomedical Engineering/ND20, Cleveland Clinic, Cleveland, OH, United States; ^4^ University of Patras, Matrix Pathobiology Res. Group, Department of Chemistry, Patras, Greece

**Keywords:** colon rectal cancer (CRC), cancer initiating cells (CICs), CD44v6, WNT3A, MDR1

## Abstract

Chemoresistance in colorectal cancer initiating cells (CICs) involves the sustained activation of multiple drug resistance (MDR) and WNT/*β*-catenin signaling pathways, as well as of alternatively spliced-isoforms of CD44 containing variable exon-6 (CD44v6). In spite of its importance, mechanisms underlying the sustained activity of WNT/*β*-catenin signaling have remained elusive. The presence of binding elements of the *β*-catenin-interacting transcription factor TCF4 in the *MDR1* and CD44 promoters suggests that crosstalk between WNT/*β*-catenin/TCF4-activation and the expression of the CD44v6 isoform mediated by FOLFOX, a first-line chemotherapeutic agent for colorectal cancer, could be a fundamental mechanism of FOLFOX resistance. Our results identify that FOLFOX treatment induced WNT3A secretion, which stimulated a positive feedback loop coupling *β*‐catenin signaling and CD44v6 splicing. In conjunction with FOLFOX induced WNT3A signal, specific CD44v6 variants produced by alternative splicing subsequently enhance the late wave of WNT/*β*-catenin activation to facilitate cell cycle progression. Moreover, we revealed that FOLFOX-mediated sustained WNT signal requires the formation of a CD44v6-LRP6-signalosome in caveolin microdomains, which leads to increased FOLFOX efflux. FOLFOX-resistance in colorectal CICs occurs in the absence of tumor-suppressor disabled-2 (DAB2), an inhibitor of WNT/*β*-catenin signaling. Conversely, in sensitive cells, DAB2 inhibition of WNT-signaling requires interaction with a clathrin containing CD44v6-LRP6-signalosome. Furthermore, full-length CD44v6, once internalized through the caveolin-signalosome, is translocated to the nucleus where in complex with TCF4, it binds to *β*-catenin/TCF4-regulated *MDR1*, or to *CD44* promoters, which leads to FOLFOX-resistance and CD44v6 transcription through transcriptional-reprogramming. These findings provide evidence that targeting CD44v6-mediated LRP6/*β*-catenin-signaling and drug efflux may represent a novel approach to overcome FOLFOX resistance and inhibit tumor progression in colorectal CICs. Thus, sustained drug resistance in colorectal CICs is mediated by overexpression of CD44v6, which is both a functional biomarker and a therapeutic target in colorectal cancer.

## 1 Introduction

Colorectal cancer (CRC) is the second leading cause of cancer-related deaths in Western countries, including the USA, with incidences increasing by 2% annually (1, 2). Despite improved survival attributed to early detection and chemotherapy with FOLFOX (1 x FOLFOX: 50 µM 5-fluorouracil [5-FU] + 10µM oxaliplatin [OXA] + 1 μM leucovorin), the first-line treatment for CRC, the emergence of multidrug resistance (MDR) that accounts for the poor tumor response to FOLFOX has limited the efficacy of this chemotherapeutic drug and finally results in therapy failure in CRC patients (3–7).

In recent years, a growing body of evidence suggests that tumor tissue is composed of a heterogeneous hierarchy of cells that differ in morphology, gene expression, proliferative capacity, and invasiveness (8). This heterogeneity originates from a small subset of cancer cells, called cancer stem cells or cancer initiating cells (CICs), that are the unique source of all tumor cells and responsible for tumor propagation and relapse (9–12). Since the first identification of CICs in breast cancer where a CD44/CD24 marker (13) was used to isolate the CICs, CICs have been now identified in a variety of solid tumors (5, 14–19) including colon carcinomas. Unlike naturally occurring somatic stem cells, CICs initiate tumorigenic activity when transplanted into animals (20, 21). Moreover, variation in the genetics and epigenetic damages of CRC patients is so different that markers to detect CICs from more differentiated progeny have not been completely informative across all patient tumors (22–25). In addition, most CIC enhancement markers mediate interactions between a tumor cell and its stromal environment, indicating that the tumorigenic characteristics associated with that marker may be lost after depletion of CICs from their microenvironment. However, the cell-surface markers that recognize CICs and have a functional role in the antiapoptotic signaling to drive tumorigenesis have remained poorly defined.

CD44 is a multi-structural and multi-functional transmembrane glycoprotein that acts as a receptor for hyaluronan (also called hyaluronic acid). *CD44* is encoded by a single gene containing 20 exons, ten of which are alternatively spliced to generate the numerous CD44 splice variants (CD44v) (15, 26). The standard isoform of *CD44* (*CD44s*) has no variant exons, is small, and is nearly ubiquitous in vertebrate cells (27). Experiments using knock-in mice that express either CD44v4-10 or CD44s isoforms have demonstrated that CD44v isoforms, promote adenoma formation in Apc (Min/+) mice but not the CD44s (28). Variant 6 of CD44 (CD44v6) participates in tumor development and progression in many ways that are restricted to stem cell subpopulations and promotes generation of gut adenomas in mouse models of familial adenomatous polyposis (28). Its role in CRC progression derives from its ability to bind ligands associated with both tyrosine kinase receptors and non-tyrosine receptors including c-Met, VEGF, TGFβ1 and ERB2 (29–39), leading to changes in biological activities such as activation of anti-apoptotic signaling and survival (40, 41). Studies have reported that CRC cells expressing CD166 (42), CD44 (43), CD44v6 (19), CD66c (42) and aldehyde dehydrogenase (ALDH1) (44) describe CRC/CIC characteristics. CD44v6 (+)/CICs have been associated with increased metastatic behavior in both pancreatic cancer (15, 45–47) and CRC (19). Further, in this study we showed that tumorigenic potentiality of the Non-CICs (CD44v6 (-) cells) was entirely lost in secondary xenograft tumors whereas tumorigenic potential of CD44v6 (+)/CICs in primary, secondary and tertiary recipients in xenograft models are confined to the small CD44v6 (+) population. Thus, the CD44v6 (+)/CICs cell population residing in the colon tumor mass is able to generate serial xenografts showing a virtually unlimited growth potential.

Recent studies indicate that several regulatory serine/arginine rich 2 splicing factors (SRSFs), such as SRSF1 (48, 49), SRSF3 (50), SRSF6 (48, 51), HNRNPA2/B1 (52), or HNRNPH (53), have oncogenic properties, whereas other factors, including RNA binding protein QKI (54), RBM5, RBM6, and RBM10 (55), act as tumor suppressors. Some of these splicing factors (SRF1/Sam68 (56) and SRSF3 (SRp20) (57) depend upon exon splicing enhancers in the case of CD44 variable exons. Their activity in promoting the inclusion of CD44 variable exons is controlled by several oncogenic signaling pathways such as, Ras/MAPK signaling (58) and *β*-catenin signaling (57), at least in part through modification of splicing factors at the level of activation (phosphorylation) (59–62). However, the signaling pathway between FOLFOX induced WNT3A activation and stimulation of alternative splicing in the nucleus is not well defined. In an unpublished study (manuscript under preparation), we found that transcription of the alternative splicing regulator SRSF3 responsible for production of CD44 variants (v6-v8) and SRF3 expression is regulated by WNT3A/*β*-catenin signaling (a FOLFOX-WNT3A-*β* catenin-TCF4-CD44v6 pathway). This might be the way FOLFOX regulates alternate splicing of CD44 in the nucleus by splicing factor 3 which is stimulated by *β*-catenin/signaling. Thus, the CD44v6 isoform is likely to be a better CIC marker than the CD44s isoform in CRCs. The WNT/ *β* -catenin signaling pathway remains important throughout life as it has crucial roles in self-renewal for adult stem and progenitor cells (63–65). WNTs are lipid-modified glycoprotein ligands that bind to both Frizzled and low-density lipoprotein receptor-related protein 6 (LRP6) (65). In physiological conditions, in the absence of a WNT signal, *β*-catenin is phosphorylated and degraded by a complex composed of glycogen synthase kinase 3*β* (GSK3*β*), Axin, adenomatous polyposis coli (APC), and casein kinase 1 (CK1). Upon binding of WNT to Frizzled and LRP5/6 (65, 66), the WNT-Frizzled-LRP5/6 complex is phosphorylated and activates disheveled protein (DVL) (67). DVL activation inhibits GSK3*β*, which subsequently decreases *β*-catenin degradation and allows for its stabilization and translocation to the nucleus, where it binds to the T-cell factor (TCF)/lymphoid enhancer factor (LEF) transcription factor and activates gene transcription (68). Endocytic adaptor DAB2 is a tumor suppressor protein (69, 70) involved in several receptor-mediated pathways (71–74). In most carcinomas, the expression of DAB2 is only expressed in low levels.

Many receptors and their protein partners, including CD44, concentrate at caveolin-1 (CAV1)-enriched lipid-rafts within the plasma membrane to mediate signaling cascades (33, 75, 76). A previous study has shown that CD44 also regulates WNT signaling in the developing brain of Xenopus Leavis embryos by association with LRP6 in the membrane (77). Many of the oncogenic activities that have been previously attributed to CD44, in particular those relevant to ligand induced translocation of receptors into discrete caveolin-microdomain in the plasma membrane that strengthen signaling pathways, could be ascribed in part to CD44-mediated caveolin-dependent endocytic signaling interactions in CRC (78).

CICs develop several mechanisms that protect them from long-term side effects caused by chemotherapeutic-drugs and make them resistant to chemotherapeutic drugs (79). In the clonal evolution model, tumor cells develop drug resistance by sequential alteration of DNA by genetic modifications. This model predicts that after chemotherapy only the drug-resistant cells within the tumor survive, proliferate, and regenerate the tumor mass that is made up of the drug-resistant cells. In the CIC model, although successful cancer therapy abolishes the bulk of proliferating tumor cells, a subset of remaining CICs can survive and promote cancer relapse due to their ability to establish higher invasiveness and chemoresistance. Generally, resistance to a clinically relevant chemotherapy combination such as FOLFOX involves the participation of a variety of cellular mechanisms, including: drug target mutations; oncogene/onco-suppressor deregulations; activation of pathways blocking the drug action; increased DNA damage repair; and overexpression of a drug extrusion pump MDR-1 (multidrug resistance-1 [P-GP, ABCB1]). The decreased influx of drugs leading to the generation of reactive oxygen species (ROS) can directly induce WNT-*β*-catenin signaling through DVl protein-mediated drug resistance that originates due to the crosstalk between tumor and stromal cells (6, 80–82). Among the WNT targets involved in drug resistance, the drug extrusion pump MDR-1 and the cell adhesion molecules from the CD44v family are highlighted (83–87). Additionally, we showed that CD44 regulates *β*-catenin-COX2 signaling in colon tumor cells (30, 88). Oncogenic CD44v expression is a downstream target gene of the WNT3A/*β*-catenin signaling pathway (89). CICs exhibit high expression levels of the two main *MDR* genes, *ABCB1* (*MDR1*) and *ABCG2* (ATP-binding cassette G2) (79). Additionally, since the basal promoter of MDR1 has several *β*-catenin/TCF4/LEF binding sites (90, 91), this protein is a target gene of the *β*-catenin/TCF4 transcriptional regulators. Thus, activation of *β*-catenin augments MDR1 expression, which confirms the direct connection between the WNT/*β*-catenin pathway and chemoresistance (90–92). Additionally, we showed that CD44 regulates *β*-catenin-COX2 signaling in colon tumor cells (30, 88). CD44v expression is downstream of the WNT signaling and induced by the *β*-catenin/Tcf-4 signaling pathway (89). A WNT3A canonical pathway (WNT3A/Frizzled/LRP6-GSK3*β*-catenin/TCF4) induces drug resistance (93, 94). However, the requirement of CD44v6 for the FOLFOX induced *β*-catenin-TCF4/MDR1 activation remains to be addressed since induction of this pathway with a chemotherapy induced WNT ligand was not tested.

Given that CICs are defined by their capacity for the development of drug-resistance, treatment failure, and tumor relapse in cancer (5, 6, 95), we investigated the mechanisms by which CICs contribute to the cell autonomous resistance against FOLFOX-chemotherapy with distinct modulation of WNT-CD44v6 signaling by regulating the endocytic fate of the CD44v6-LRP6 receptor interaction in membrane microdomain. We base this investigation on the following observations.

1) Our results identify that FOLFOX treatment induced WNT3a secretion, which stimulated a positive feedback loop coupling *β*‐catenin activation signals and CD44v6 splicing for sustained drug resistance. In addition, CD44v6 could sustained cell cycle S phase responses through a positive feedback loop, where this isoform is speculated to be important for a secreted factor WNT3A signaling. 2) *β*-catenin interacts with TCF4 binding elements in the *MDR1* and *CD44* gene promoters. This suggests that crosstalk between WNT/*β*-catenin/TCF4-activation and the expression of the CD44v6 isoform mediated by FOLFOX could be a fundamental mechanism of FOLFOX resistance in colorectal CICs. 3) FOLFOX-mediated sustained WNT/*β*-catenin signaling requires the formation of a CD44v6-LRP6-signalosome in caveolin-microdomains, leading to increased FOLFOX efflux. Conversely, in the absence of FOLFOX, DAB2 links CD44v6 and LRP6 in clathrin containing vesicles that attenuate WNT/*β*-catenin signaling to maintain drug sensitivity in sensitive cells. 4) In FOLFOX resistant cells, CD44v6 is internalized through the caveolin containing signalosome, and this signalosome is recruited to the endosome for sorting of CD44v6-*β*-catenin/TCF4-complex vesicles, which are then destined to the nucleus. In the nucleus, CD44v6 binds to various promoters, including *β*-catenin/TCF4-regulated promoters, leading to FOLFOX resistance through transcriptional reprogramming. TCF4 maintains distinctive transcriptional programs *via* interactions with MDR1 and *CD44* promoters and sustains CD44v6-mediated autonomous-resistance in CICs.

## 2 Materials and methods

### 2.1 Materials

Dulbecco’s Modified Eagle’s Medium (DMEM), Eagle’s Minimum Essential Medium (EMEM), McCoy’s 5A Medium, F-12K Medium, Leibovitz’s L-15 Medium, L-Glutamine, Sodium pyruvate, Penicillin (100 µg/ml) and Streptomycin (100 µg/ml), sodium pyruvate, 0.05% EDTA solution (Versene), Phosphate buffered saline (PBS, Calcium and Magnesium free), and 0.05% Trypsin were from Corning Inc. Fetal Bovine Serum (FBS) was from Atlanta Biologicals. Amphotericin B was from Hyclone, Thermo Fisher Scientific. Waltham, MA, USA. Nonidet P-40, EGTA, sodium orthovanadate, glycerol, phenylmethylsulphonyl fluoride, leupeptin, pepstatin A, aprotinin and HEPES were from Sigma-Aldrich, Inc. St. Louis, MO, USA. Recombinant human WNT3A protein (5036-WN) was from R&D Systems, Inc. Minneapolis, MN, USA). Blocking antibody for protein WNT3A (703666, Rabbit monoclonal IgG clone 1H12L14) was from Thermo Fisher Scientific, Waltham, MA, USA. The anti-Active-*β*-catenin antibody (05-665, anti-ABC antibody clone 8E7) was from Millipore Sigma, Burlington, MA, USA, and the anti- *β*-catenin antibody (610153, mouse IgG1, BD, Tempe, Arizona, USA) was used for total *β*-catenin detection in western blotting analysis. The antibodies p-LRP6 (Serine 1490) (#2568, Rabbit IgG), LRP6 (#2560, Rabbit mAb clone C5C7), TCF4 antibody (#2569, Rabbit mAb clone C48H11) were from Cell Signaling Technology, Inc. Danvers, MA, USA. P-Glycoprotein (MDR1) western blotting antibody (PA5-28801, Rabbit Polyclonal against Human) was from Invitrogen Thermo Fisher Scientific, Waltham, MA, USA. *β*-tubulin Antibody (D-10) (sc-5274, Mouse monoclonal IgG_2b_ κ, SCBT), Mouse anti-rabbit IgG-HRP (sc-2357, IgG, SCBT), Rabbit anti mouse IgG-HRP (sc-358914, IgG, SCBT), and Western blotting Luminol reagent (sc-2048, SCBT) were purchased from Santa Cruz Biotechnology, Inc. Dallas, Texas, USA. Blocking antibodies for CD44v6 (BBA13, Monoclonal Mouse IgG_1_ Clone # 2F10, R&D), and isotype control (MAB002, IgG1, R&D) and the mouse IgG1 antibodies were from R&D Systems, Inc. Minneapolis, MN, USA. Radiocarbon-labeled oxaliplatin ([^14^C]oxaliplatin) was purchased from Amersham Biosciences, Piscataway, NJ, USA.

### 2.2 Cell lines

Human colorectal adenocarcinoma cell lines: 1) WIDR (CCL-218) was maintained in Eagle’s Minimum Essential Medium (EMEM) +10% FBS; 2) LOVO (CCL-229) was maintained in F-12K Medium 2 mM L-glutamine and 1500 mg/L sodium bicarbonate; 3) HT29 (HTB-38) was maintained in McCoy’s 5A medium; 4) SW480 (CCL-228) was maintained in Leibovitz’s L-15 Medium; and 6) SW948 (CCL-237) was maintained in Leibovitz’s L-15 Medium that was purchased from ATCC, Manassas, Virginia. The cell lines were maintained in medium mentioned next to the cell line in humidified atmosphere in the presence of 10% FBS, Penicillin (100 µg/ml) and Streptomycin (100 µg/ml), 5% CO_2_ at 37°C. HCA-7 colony 29 was purchased from European Collection of Authenticated Cell Cultures and maintained in DMEM + 10% FBS + 2 mM L-Glutamine + 110 mg/L sodium pyruvate.

### 2.3 Generation of drug resistant cells

**Figure 1:**
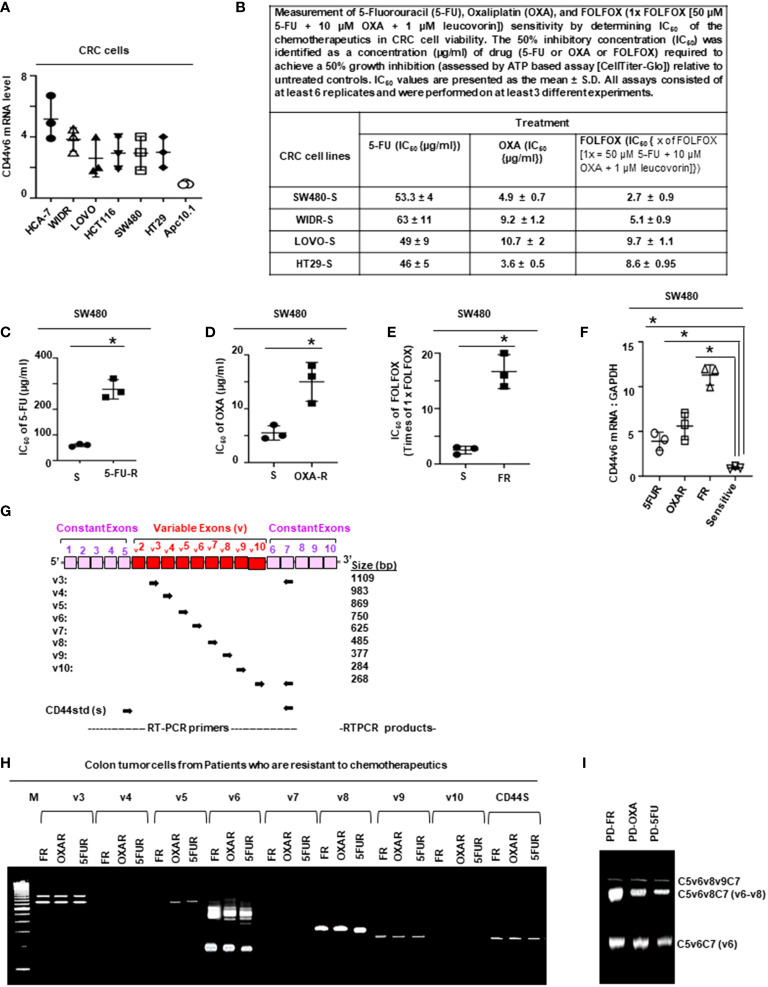
FOLFOX induces CD44v6 expression. **(A)**, QPCR data for CD44v6 expression in 6 CRC cell lines are shown as fold change relative to pre-neoplastic Apc 10.1 cells as controls. **(B–E)**, The concentrations (IC_50_) (µg/ml) of (5-FU) and (OXA) required to achieve a 50% growth inhibition relative to untreated controls using the ATP Glo-growth assay are shown **(B)**. The IC_50_ values of 5-FU **(C)**, OXA **(D)**, and FR **(E)** for sensitive (S) and corresponding FOLFOX resistant (FR) cells are shown. **(F)**, Real-time PCR data for the CD44v6 expression are presented for SW480 tumor cells resistant to either 5-Fluorouracil (5FUR), oxaliplatin (OXAR), or FOLFOX (FR) compared to sensitive (S) pairs of cells. Gene expression was normalized to the reference gene GAPDH. **(G)**, Constant and variable exons are shown for the PCR primers used to amplify CD44 variable (v) and standard (s) isoforms in the human CD44v6 gene. The primers for both the v6 and standard isoforms (CD44s) predominantly generate one PCR product c5v6c7 (v6) for CD44v6 and C5C7 for CD44s, whereas the primers for the v8 variants amplify two splice variants C5v6v7v8C7 (v6-v8) and C5v8C7 (v8). These PCR products are depicted in panel H and panel I experiments. **(H)**, RT-PCR results are shown for the CD44v isoforms using the different primers in the tumor cells derived from colorectal patients (PD) who were resistant to 5FU, OXA and FOLFOX (PD-5-FU, PD-OXA and PD-FR). **(I)**, RT-PCR results are shown for the CD44v isoforms using the different primers (C5v6v8v9C7, C5v6v8C7 and C5v6C7) in the PD-FR, PD-OXA and PD-5-FU cells. Data are presented as Mean ± SD from n = 3-6 independent replicates in three independent experiments. All semi quantitative RT-PCR data are representative of three experiments **(C–E)**, *P < 0.05 was considered significant, 50% inhibitory concentration (IC_50_) for 5-FU in SW480-5FUR cells was compared with SW480-S cells **(C)**, for OXA in SW480-5FUR cells was compared with SW480-S cells **(D)**, and for FOLFOX n SW480-5FUR was compared with SW480-S cells **(E, F)**, *P < 0.05 considered significant, fold expressions of CD44v6mRNA in SW480-5FUR, SW480-OXAR, and SW480-FR cell were compared to control (SW480-S) cells.

To determine the mechanisms of drug (FOLFOX) resistance, we selected three cell lines (HT29, SW480, WIDR and LOVO cells [CRC cells used in **Figure 1B**]) out of 7 cell lines (**Figure 1A**), which have low basal levels of CD44v6 mRNA expression. To generate these drug resistant cells, we first determined IC_50_ values of the parent CRC cells for 5-Flourouracil (5-FU) and oxaliplatin (OXA) (see **Figure 1B**), because these molecules are the components of FOLFOX. The 50% inhibitory concentration (IC_50_) was identified as a concentration of drug required to achieve a 50% growth inhibition relative to untreated controls. Next, we determined IC_50_ values of the parent sensitive SW480-S, HT29-S, WIDR-S, and LOVO-S cells for FOLFOX (1x FOLFOX = 50 µM 5-FU + 10 µM OXA + 1 µM leucovorin). The average IC_50_ values for tested CRC cells are in **Figure 1B**. The 5-FU resistance (5-FUR), and oxaliplatin resistance (OXAR) cells were generated by incubating the parental sensitive SW480-S, or HT29-S, or WIDR-S, and LOVO-S cells with repeated exposure to increasing concentrations of the drug (100 µM 5-FU) for generating 5-FUR cells; and 20 µM OXA for generating OXAR cells for 3 days. For generating FR cells from SW480-S and HT29-S, we incubated each of the two cells to 5 x FOLFOX for 3 days. To generate FR cells from WIDR-S and LOVO-S, we incubated each of the two cells to 10 x FOLFOX for 3 days. This exposure and withdrawal cycle was repeated five times for the above mentioned doses of each of the drug. The surviving 5-FUR, OXAR and FR clones were cultured in normal medium for 5 days and maintained with selection pressure of half the average IC_50_ dose of CRC cells for 5-FU, OXA and FOLFOX (**Figure 1B**). The resistances of these resistant clones were compared to sensitive pairs by determining the numbers of colonies in soft agar growth with 1x FOLFOX - 5x FOLFOX treatments

### 2.4 Tissue collection, isolation of CICs

All human tissues were acquired from primary human colorectal tumor patient specimens undergoing colorectal resection, in agreement with human experimental guidelines and the ethical standards of the institutional review board (IRB). Human protocols were approved by the IRB of the Medical University of South Carolina. The IRB has determined that this research project meets the criteria for ‘Non Human Subjects’ research. Patient-derived (PD) biopsies collected from 5-FU resistant (PD-5FUR), Oxaliplatin resistant (PD-OXAR), and FOLFOX resistant (PD-FR) tumor specimens and our FOLFOX resistant (FR), Oxaliplatin resistant (OXA-R), 5-flurouracil resistant (5-FUR) cell clones, and parental SW480 cells (sensitive [s]) cells were maintained through subcutaneous (SQ) xenografts in the flanks of immunocompromised (NOD-SCID/IL2Rγnull [NSG]) mice and in SCID mice, respectively. *Ex vivo* cultures from the fresh normal colonic tissue and colorectal PD-FR, PD-OXAR, PD-5FUR, SW480-FR, SW480-OXAR, SW480-5FUR, and SW480-S SQ tumors were rinsed in DMEM (Life Technologies) supplemented with 200 units/mL of penicillin, 200 µg/mL of streptomycin, and 4 units/mL of amphotericin B. After mincing, they were incubated with 300 units/mL of collagenase (Worthington Biochemical) at 37°C for 3 hours. A single cell suspension was obtained by filtration through a 40 µm filter. After discarding lymphocytes by gradient centrifugation, *Ex vivo* cultures were cultured in DMEM (Life Technologies) supplemented with 200 units/mL of penicillin, 200 µg/mL of streptomycin, and 4 units/mL of amphotericin B, serum-free media with basic fibroblast growth factor (bFGF, 10 ng/ml; R&D Systems) and epidermal growth factor (EGF, 10 ng/ml; R&D Systems). The cells were processed for sphere formation. Sphere-propagated cells were subjected to fluorescence-activated cell sorting (FACS) buffer [Phosphate-buffered saline (PBS) + 2% BSA + 1 mM EDTA + 0.1% sodium azide]. They were then incubated with FC blocking reagent (Millenyi Biotech) and stained with directly conjugated antibodies by incubating on ice for 20 minutes. They were then sorted in a Mo Flo cell sorter for CD44v6 CICs (CD44v6 high (+) by CD44v6-PE) and tested for tumor sphere formation at 37°C in 5% CO_2_.

### 2.5 Cell viability and apoptosis assays

Five thousand cells were plated in triplicate into 96-well plates containing appropriate growth media and incubated overnight. After 16 hours growth, cultures were incubated in media containing no serum for 16 hours at 37°C in 5% CO_2_, 95% air. Vehicle or chemotherapy drug was added to the plate. In each experiment, a total of five plates (6 wells/treatment) were used. Experiments were repeated 3 times. The growth of the cells was determined by measuring increases in readings of ATP levels for viability (CellTiter-Glo, Promega). Cell apoptosis was determined by the Caspase-Glo^®^ 3/7 assay (Promega) using DEVD-amino luciferin substrate. The luminescent signal is proportional to caspase 3/7 activity and measured using a luminometer (Perkin Elmer).

### 2.6 Flow cytometric analysis of CIC cells

Flow cytometry was done using a FACS Cell Sorter. To enrich cells for CICs, single cells were labeled with a phycoerythrin (PE)-conjugated monoclonal antibody against CD44v6 (Miltenyi Biotec), and then analyzed for the expression of Fluorescein-5-isothiocyanate (FITC) with conjugated monoclonal antibody against EpCAM (R&D Systems). Purified CD44v6 (+)/EpCAM (+), and CD44v6 (–)/EpCAM (+) cells from various tumors were cultured separately and grown in fresh CIC growth medium (see below) for 2 weeks. Then, the cultured CD44v6 (+)/EpCAM (+) and CD44v6 (-)/EpCAM (+) cells were subjected to flow cytometric analysis for isolation of CD44v6 (+)/EpCAM (+)/ALDH1 (+), and CD44v6 (–)/EpCAM (+)/ALDH1 (+) cells using a FITC-conjugated monoclonal antibody against ALDH1 and grown in fresh medium for 2 weeks. Cultured CD44v6 (+)/EpCAM (+)/ALDH1 (+) cells and CD44v6 (-)EpCAM (+)/ALDH1 (+) cells were subjected to flow cytometric analysis for isolation of CD44v6 (+)/EpCAM (+)/ALDH1 (+)/CD133 (+) (designated as CICs), and for CD44v6 (-)/EpCAM (+)/ALDH1 (+)/CD133(+) cells (designated as Non-CICs) using a FITC-conjugated monoclonal antibody against CD133.

CICs were cultured in serum-free media with basic fibroblast growth factor (bFGF, 10 ng/ml; R&D Systems) and epidermal growth factor (EGF, 10 ng/ml; R&D Systems). For isolation of CAFs, surgical tissues were similarly dissociated into single-cell suspensions, and PDGFRα-expressing cells were sorted using flow cytometry (FACS Aria II). Cells were then cultured in DMEM with 10% BSA. For cell counting before each experiment, a single-cell suspension was achieved using TrypLE (Invitrogen) dissociation.

### 2.7 Tumor sphere formation

An optimized serum substitute (1 x B27 supplement) (Creative Bio array, Shirley, NY, USA) was freshly added to tumor formation medium (500 ml Dulbecco’s Modified Eagle Medium/F12) containing 20 ng/ml epidermal growth factor, 10 ng/ml basic fibroblast growth factor, 5 mg/ml insulin, and 0.4% bovine serum albumin. After harvesting the cells, 200 live cells/200 µl of tumor sphere medium were suspended in ice. We followed the tumor sphere formation assay protocol from the Creative Bio-array (Shirley, NY, USA). After stipulated times of incubation, tumor sphere numbers were counted under a phase-contrast microscope using the 40X magnification lens. Data are presented as a percentage of wells containing tumor spheres compared to the total number of wells.

### 2.8 Cell lysis, immunoprecipitation and immunoblot analysis

Cells were cultured until they were 75% confluent. They were then washed twice at 4°C with phosphate-buffered saline (PBS), and harvested with 0.05% Versene. The cells were pelleted by centrifugation at 5,000 x g for 2 minutes at 4°C. The pellets were treated with the lysis buffer (containing 1% Nonidet P-40, 0.3 M NaCl, 1.5 mM MgCl2, 0.2 mM EDTA, 5 mM sodium orthovanadate, 10% (v/v) glycerol, 100 µg/ml phenylmethylsulphonyl fluoride (PMSF), 1 µg/ml leupeptin, 1 µg/ml pepstatin A, 1 µg/ml aprotinin, and 50 mM HEPES, pH 7.5) for 30 minutes. For immunoprecipitation, the cell extracts (1 mg total proteins) were precleaned by rotation for 1 hour with 20 µl of protein G-Sepharose beads (Santa Cruz, CA). The precleaned supernatants were incubated with antibodies needed for each specific experiment overnight. After incubation with 20 µl of protein G-Sepharose beads for 1 hour, the suspension was centrifuged, and pellets washed and collected as immunoprecipitation complexes. Western blotting was done as previously described (38, 39, 96–98). Each protein was analyzed in samples from at least three independent experiments from each set of tumor cells, CICs and CAFs. Images were recorded using a luminescent image analyzer, and the intensities of the bands were quantitated by densitometry (NIH Image J software). Each protein was analyzed in samples from at least three independent experiments from each set of tumor cells, CICs and CAFs.

### 2.9 Cell cycle synchronization and analysis of cell cycle profile

For synchronization into the G2/M phase of the cell cycle, SW480 cells were treated with 100 ng/mL of nocodazole (Sigma-Aldrich, Inc. St. Louis, MO, USA) for 16 hours. To study the enrichment of the cells into the different cell cycle phases, cells were released by drug removal by washing twice with E8 media (E8 medium consisted just of insulin, selenium, transferrin, L-ascorbic acid, FGF2, and TGF*β* in DMEM/F12 with pH adjusted with NaHCO_3_). Sixteen hours after release from G2/M phase, when 97% of the cells were in G1 phase (cell cycle analyzed by flow cytometry) the cells were transfected with either non-targeted (Control) or CD44v6 shRNA. Twenty four hours after transfection, the cells were incubated in low serum medium (0.5% serum) with 20 ng/ml WNT3A or with 1 x FOLFOX and stimulated for 30 minutes, 1, 2, 8, 12, and 24 hours. Cell cycle profiles were analyzed using the Click-iT™ EdU Cell Proliferation Kit (Invitrogen Thermo Fisher Scientific, Waltham, MA, USA) following the manufacturer’s instructions. Briefly, SW480 cells cultured cells were incubated at 37°C with 10 mM EdU (5-ethynyl-20-deoxyuridine) for 1 hour and harvested using cell dissociation buffer (Gibco Thermo Fisher Scientific, Waltham, MA, USA). After three washes with PBS/1% BSA, cells were fixed with 4% paraformaldehyde for 15 min at room temperature and released from paraformaldehyde by washing three times with PBS/1% BSA. Cells were then permeabilized for 15 minutes with saponin-based permeabilization/wash buffer and incubated with the Click-iT EDU Alexa Fluor reaction cocktail for 30 min in dark. Click-iT Assay Kits for Flow Cytometry provide the measurement by tracking new DNA content in fixed cells that were washed once with permeabilization/wash buffer and stained for DNA,content in fixed cells when combined with RNAse using the FxCycle™ Far Red stain (F10348, Invitrogen Thermo Fisher Scientific, Waltham, MA, USA) Cells were analyzed on the flow cytometer with FlowJo software.

### 2.10 Lipid-raft isolation

All procedures were done at 4°C. Cells were scraped into buffer containing 1% cold Triton X-100 buffer (20 mM Tris-HCl, pH 7.4, 150 mM NaCl, 1% Triton X-100, 1% deoxycholate, 0.1% sodium dodecyl sulfate [SDS], 1 mM EGTA, 1 mM sodium orthovanadate with a cocktail of protease inhibitors at final concentrations of 0.2 mM aminoethyl-benzene sulfonyl fluoride, 1 µg/ml aprotinin, 10 µM pepstatin, 3 µM E-64, 10 µg/ml leupeptin, 2 µM pepstatin, and 50 µg/ml calpain inhibitor I) and lysed on ice for 30 minutes. After centrifugation at 800 x g to remove nuclei and cell debris, lysates were subjected to sucrose gradient fractionation as described previously (99). An equal volume of each fraction was boiled for 3 minutes in SDS-Lammeli sample buffer and subjected to western blotting analyses as described previously (38, 39, 96–98, 100, 101). On the other hand, the Triton-insoluble rafts and Triton-soluble fractions were diluted with an equal volume of extraction buffer (25 mM HEPES, pH 7.6, 0.3 M NaCl, 1.5 mM MgCl2, 0.2 mM EDTA, 1% Nonidet P-40, and 0.5 mM dithiothreitol) and subjected to immunoprecipitation as described previously.

### 2.11 Endosome isolation

Endosomes from SW480-FR-NON-CICs/CD44v6 cell clones expressing actin binding NLS mutant (nuclear localization signal mutant) and Δ67 mutant cells (**Figures 9** and **10**) were isolated by sucrose density gradient (102). All operations were done at 4°C. The cells were grown in plates and washed with PBS three times to remove growth medium. The cells in 0.5 ml of homogenization buffer (250 mM sucrose, 1 mM EDTA, 1 mM phenylmethylsulphonyl fluoride (PMSF)) were lysed by passing through a 22G needle and syringe. After centrifugation at 1000xg for 10 minutes, the supernatant was adjusted to 25% sucrose/1 mM EDTA. Step gradients in four layers were set up in SW41Ti tubes and centrifuged at 100,000×g for one hour. Fractions (2 ml) were collected from top to bottom. The densities of the fractions were measured by refractometry. The fractions were analyzed by western blotting.

### 2.12 Plasmids and reporter assays

#### 2.12.1 Expression vectors

pcDNA-Wnt3A-V5 was a gift from Marian Waterman (Addgene plasmid # 35927; http://n2t.net/addgene:35927; RRID : Addgene #3 5927), human pcDNA3-*β*-catenin was a gift from Eric Fearon (Addgene plasmid # 16828; http://n2t.net/addgene:16828; RRID : Addgene # 16828). CD44v6 specific PCR amplification products were isolated with polyadenylated RNA from the HT29 cell line. The PCR product was cloned in the pcDNA3.1 vector and used as previously described. Myc-tagged human full length TCF4E pcDNA3 was a gift from Frank McCormick (Addgene plasmid # 32738; http://n2t.net/addgene:32738; RRID: Addgene # 32738). pDONR223_DKK1_WT was a gift from Jesse Boehm & William Hahn & Root (Addgene plasmid # 82250; http://n2t.net/addgene).

#### 2.12.2 Reporter vectors

The MDR1 and CD44v6 reporter constructs were synthesized by Bio basic (US) and cloned into the firefly pGL3-basic vector (Promega) upstream of the Luciferase reporter gene. The constructs named: 1) mdr1 (a) contains the basal promoter and multiple (four) TCF binding sites (−1301/+1); 2) mdr1 (b) contains the basal promoter and one TCF binding site (−1067/+1); and 3) mdr1 (c) contains just the basal promoter. The constructs named: 1) CD44v6 (a) contains one TCF binding site (-1700/500); and 2) CD44v6 (b) contains basal promoter and two TCF binding sites (-2100/500). The M50 Super 8x TOPFlash vector (plasmid 12456) with a luciferase gene under the control of seven TCF/LEF-binding sites and the corresponding M51 Super 8x FOPFlash vector (plasmid 12457) with mutated TCF/LEF-binding sites were obtained from Addgene (Cambridge, MA, USA). The normalization vector pRL-TK renilla with a HSV-TK promotor driving Renilla luciferase was purchased from Promega.

#### 2.12.3 Transient transfection and luciferase reporter assay

For the transient assays, 1.0 x 10^5^ cells from both cell lines were transfected using Lipofectamine LTX 2000 (Invitrogen) with 1 μg of each Luciferase construct and 100 ng of pRL-SV40 vector (Promega), according to the manufacturer’s instructions. Firefly and Renilla Luciferase activities were measured in cell lysates 48 hours after transfection using the DualGlo Luciferase Assay System (Promega) on a Veritas TM Microplate Luminometer (Perkin Elmer) following the manufacturer’s protocol. All experiments were done in triplicate. Ratios of Renilla luciferase readings to firefly luciferase readings were taken for each experiment, and triplicates were averaged. The average values of the tested constructs were normalized to the activity of the empty pGL3-basic vector, which was arbitrarily set at value 1.

#### 2.12.4 *β*-catenin/TCF Reporter assays

All reporter gene assays were done in 96-well plates. PD-FR/CICs or CD44v6 overexpressing SW480-FR/SQ/Non-CICs (Non-CICs/CD44v6) (1.0 × 10^4^/well) were transfected with Super TOPFlash reporter (25 ng) and TK-Renilla (5 ng), and with the respective plasmid DNA as indicated using Lipofectamine™ 3000 transfection reagent according to the manufacturer’s protocol. Each transfection was adjusted to 150 ng DNA/transfection with pcDNA3.1 empty vector. Where indicated, cells were transfected at 50–70% confluency with shRNA constructs using Lipofectamine™ 3000 transfection Reagent in 6 cm petri dishes according to the manufacturer’s protocol 24 hours before seeding the cells for the reporter assays. 50 ng/ml of WNT3A was added 24 hours after DNA transfection. Cells were lysed 72 hours after DNA transfection with 1 × Passive Lysis Buffer (Promega), and the luciferase activity was measured using the Luminescence counter (PerkinElmer). TOPFlash experiments were normalized to co-transfected Renilla gene expression. In parallel to the reporter assay, transfected CICs and Non-CICs/CD44v6 cells (CD44v6 overexpressed Non-CICs) were subjected to western blotting analysis to detect MDR1, CD44v6 and *β*-catenin for CD44v6-*β*-catenin-MDR1 signaling.

### 2.13 Primer design and PCR

#### 2.13.1 RNA extraction and cDNA synthesis were done following published work

Total RNA was isolated from cells using the RNeasy mini kit (Qiagen) according to the standard protocol provided by the manufacturer, with on-column DNA digestion. Five hundred ng of RNA was used for cDNA synthesis. One ml primer, 1 ml buffer (5x), 0.5 μl RNase inhibitor, 1 ml dNTP (10 mM) and 0.5 ml Reverse Transcriptase (Thermo Fisher Scientific) were mixed in a microtube (0.2 ml) (103). The synthesis was done at 50°C for 60 minutes in a thermal cycler (Bio Rad).

#### 2.13.2 Primer design and semiquantitative RT-PCR

Primers were designed by online Primer Quest Tool (https://www.idtdna.com/PrimerQuest/Home/Index). The quality of designed primers was analyzed by Oligoaniline Tool software. The semi-quantitative PCR primer sequences used for *CD44* exon specific PCRs are given in **Tables 1** and **2**. Semi-quantitative PCR was done using different amounts of cDNA of RNA samples. One ml of forward (F) and of reverse (R) primers were used. For each sample, PCR was repeated three times. The reaction contained 1 ml of each cDNA sample, 0.5 ml of each primer, 5 ml Taq DNA Polymerase 2× Master Mix Red (Amplicon Co.) and 3 μl dd water in a final volume of 10 μl. Before the main reactions, the PCR conditions, including thermal conditions, and the number of cycles and the cDNA concentrations, were optimized (5, 103, 104). During the main PCR cycles, temperature conditions included one initial denaturation cycle (3 minutes at 95°C) followed by 35 cycles with a denaturation step for 5 seconds at 95°C and a combined annealing and extension step for 35 seconds at 61°C (5, 103, 104). The PCR products were electrophoresed on agarose 2.5%, stained with ethidium bromide and photographed. The analysis of band intensities was done by ImageJ software.

**Table 1:** CD44 exon specific PCR examined using 5’ primers complementary to individual variable exons and a primer to the 3’ constitutive exon 7.

Genes	Accession number	Primers	
		Forward sequence (5’–3’)	Reverse sequence (5’–3’)
C_5_		AAGACATCTACCCCAGCAAC	
C_7_			TTTGCTCCACCTTCTTGACTCC
**h-CD44V2**	**NM_001001389.2**	**GAT GAG CAC TAG TGC TAC AG**	**TTTGCTCCACCTTCTTGACTCC**
**h-CD44V3**	**NM_001001390.2**	**ACG TCT TCA AAT ACC ATC TC**	**TTTGCTCCACCTTCTTGACTCC**
**h-CD44V4**	**NM_001001391.2**	**TCA ACC ACA CCA CGG GCT TT**	**TTTGCTCCACCTTCTTGACTCC**
**h-CD44V5**	**NM_001001392.2**	**GTA GAC AGA AAT GGC ACC AC**	**TTTGCTCCACCTTCTTGACTCC**
**h-CD44V6**	**NM_001202555.2**	**CAG GCA ACT CCT AGT AGT AC**	**TTTGCTCCACCTTCTTGACTCC**
**h-CD44V7**	**NM_001202556.2**	**CAG CCT CAG CTC ATA CCA G**	**TTTGCTCCACCTTCTTGACTCC**
**h-CD44V8**	**NM_001202557.2**	**TCC AGT CAT AGT ACA ACG CT**	**TTTGCTCCACCTTCTTGACTCC**
**h-CD44V9**	**XM_011520485.2**	**CAG AGC TTC TCT ACA TCA CA**	**TTTGCTCCACCTTCTTGACTCC**
**h-CD44V10**	**XM_005253238.3**	**GGT GGA AGA AGA GAC CCA AA**	**TTTGCTCCACCTTCTTGACTCC**
**h-CD44C5V6**		**ATCCCTGCTACCATCCAGGCAAC**	**TTTGCTCCACCTTCTTGACTCC**
**h-CD44s**	**X155150 (EMBL/Genebank)**	**AAGACATCTACCCCAGCAAC**	**TTTGCTCCACCTTCTTGACTCC**
**h-GAPDH**	**NM_002046.7**	**ACC ACA GTC CAT GCC ATC A**	**TCC ACC ACC CTG TTG CTG TA**

**Table 2:** CD44 exon specific PCR examined using 3’ primers complementary to v6 and v8 exons and a primer to the 5’ constitutive exon 5.

Genes	Accession number	Primers	
		Forward sequence (5’–3’)	Reverse sequence (5’–3’)
**C_5_**		**CATCCCAGACGAAGACAGTC**	
**h-CD44V6**	**NM_001202555.2**		**CAG GCA ACT CCT AGT AGT AC**
**h-CD44V8**	**NM_001202557.2**		**GTTGTCATTGAAAGAGGTCCT**
**h-CD44s**	**X155150 (EMBL/Genebank)**	**CATCCCAGACGAAGACAGTC**	**TTTGCTCCACCTTCTTGACTCC**
**h-GAPDH**	**NM_002046.7**	**ACC ACA GTC CAT GCC ATC A**	**TCC ACC ACC CTG TTG CTG TA**
**C_7_**			**TTTGCTCCACCTTCTTGACTCC**

#### 2.13.3 *Quantitative real-time RT–PCR (QPCR)*

Total RNA was isolated from cells after various treatments and transfections as described in the figure legends for each specified experiment using the RNeasy mini kit (Qiagen) according to the standard protocol provided by the manufacturer, with on-column DNA digestion. RNA integrity and concentration were analyzed using Bioanalyzer, and 100 ng of RNA was retrotranscribed into cDNA using the First Strand cDNA synthesis kit from Roche Applied Science (Qiagen). SYBR Green technology (Bio-Rad) was used for all real-time PCR experiments. Amplification was done with the real-time PCR analyzer (Bio-Rad). The PCR mixture (25 µl) contained 12.5 µl of 2 SYBR Green PCR Master Mix (Bio-Rad), 5 µl of diluted RT product (1:20), and 0.5 µM sense and antisense primer sets. The QPCR primers used in this study in analyses of various genes associated with CIC stemness function are presented in **Table 3**. The real-time PCR assays were done in three individual experiments with duplicate samples using standard conditions (5, 104) in a CFX96 real-time PCR detection machine. After incubations at 95°C for 3 minutes, the amplification protocol consisted of 50 cycles of denaturing at 95°C for 10 seconds, followed by annealing and extension at 60°C for 30 seconds. The standard curve was made from a series dilution of template cDNA. Expression levels of tested genes were calculated after normalization with the housekeeping gene *GAPDH* or *β*-actin (5, 104).

**Table 3:** Real-time PCR (QPCR) primers for various genes associated with CICs stemness function.

Genes	Accession number	Primers	
		Forward sequence (5’–3’)	Reverse sequence (5’–3’)
**SOX-2**	**NM_003106**	**GGACTGAGAGAAAGAAGAGGAGAG**	**CGCCGCCGATGATTGTTATTA**
**ALDH1**	**NM_000689.5**	**TGGCTTATCAGCAGGAGTGT**	**GCAATTCACCCACACTGTTC**
**OCT4**	**NM_002701.6**	**GGAGGAAGCTGACAACAATGA**	**CTCTCACTCGGTTCTCGATACT**
**c-MYC**	**NM_002467.6**	**AAGCTGAGGCACACAAAGA**	**GCTTGGACAGGTTAGGAGTAAA**
**Nanog**	**NM_024865.4**	**GCCTGTAGTCCCAGCTATTTG**	**GGAGTGCAGTGGTGTGATATT**
**TWIST1**	**NM_000474.4**	**AGACTCTGGAGCTGGATAACT**	**GCCTGTCTCGCTTTCTCTTT**
**EpCAM**	**NM_002354.3**	**AGCTGGTGTTATTGCTGTTATTG**	**GCATCTCACCCATCTCCTTTAT**
**MDR1**	**NM_001348945**	**TGCTGGTTGCTGCTTACA**	**GCCTATCTCCTGTCGCATTATAG**
**CD44v6**	**NM_001202555.2**	**GACAGAATCCCTGCTACCAATAG**	**TCCTTCGTGTGTGGGTAATG**
**NFkB**	**NM_003998.4**	**GTGACAGGAGACGTGAAGATG**	**TGAAGGTGGATGATTGCTAAGT**
**E2F1**	**NM_005225.3**	**TCCCTGAGCTGTTCTTCTG**	**CCTCCCTCACTTTCCCAATAAA**
**STAT3**	**NM_139276.3**	**GAGAAGGACATCAGCGGTAAG**	**CAGTGGAGACACCAGGATATTG**
**RUNX2**	**NM_001024630.4**	**CGGAATGCCTCTGCTGTTAT**	**TGTGAAGACGGTTATGGTCAAG**
**Snail**	**NM_005985.4**	**ACTATGCCGCGCTCTTTC**	**GCTGGAAGGTAAACTCTGGATTA**
**P300**	**NM_001429.4**	**ACTTTGGAGGCACTTTACCG**	**CTGTCCAGTGTCTAACTTCCTC**
**YB-1**	**NM_004559.5**	**TCAATGTAAGGAACGGATATGGT**	**AACATCAAACTCCACAGTCTCT**
**AP-1**	**NM_002228.4**	**GGACACGCCTTCTGAACG**	**CGGAGTCCAGTGTGGTTTG**
**Notch1**	**NM_017617.5**	**ATCAACTCACACGCCGAC**	**TGCATATCTTTGTTAGCCCCG**
**GAPDH**	**NM_002046.7**	**GAAGGTGAAGGTCG**	**CTTCCCGTTCTCAG**
***β*-actin**	**NM_001904.4**	**AGAAAATCTGGCACCACACC**	**AGAGGCGTACAGGGATAGCA**
**C/EBP**	**NM_001806.4**	**CGAACTGGACACGCTGC**	**ACCCCAAACCACTCCCT**
**TCF4**	**NM_001083962.2**	**GGACCTTCTCATAATGGAGCC**	**TGGTTTGGCAGAAGAGAATGG**

### 2.14 RNA silencing

For determining shRNA sequences used in this study: 1) coding nucleotide sequences of the genes were obtained from the NCBI, National Institutes of Health, website (www.ncbi.nlm.nih.gov); 2) hairpin shRNAs were designed to target a transcript sequence using the Broad Institute GPP Web Portal (http://portals.broadinstitute.org/gpp/public/); and 3) sequences for cloning in pSico/pSicoR vectors were designed following the MIT Jackson Lab website (http://web.mit.edu/jacks-lab/protocols). The resulting pSicoR-CD44v6 shRNA1 (CD44v6 sh1), pSicoR-CD44v6 shRNA2 (CD44v6 sh2), pSicoR-WNT3A shRNA1 (WNT3A sh1), pSicoR-WNT3A shRNA2 (WNT3A sh2), pSicoR-*β*-catenin shRNA1 (*β*-catenin sh1), pSicoR-*β*-catenin shRNA2 (*β*-catenin sh2) transfectants constitutively silence respective CD44v6, WNT 3A and *β*-catenin genes in the cells. pSicoR-Non targeted shRNA (NT sh) transfectants were used as control to the above shRNA transfectants (see **Table 4** for shRNA sequences used in this study).

**Table 4:** shRNA sequence in pSico and pSicoR vectors (https://web.mit.edu/jacks-lab/protocolsl).

Genes	Accession number	Primers	
		Sense sequence (5'-3')	Antisense sequence (5'-3')
CD44v6 shRNA1	Nl.1_001202555.2	TCCTCCCAGTATGACACATATTTTCAAGAGAAA TATGTGTCATACTGGGAGGTTTTTTC	TCGAGAAAAAACCTCCCAGTATGACACATATT TCTCTTGAAAATATGTGTCATACTGGGAGGA
CD44v6 shRNA2	NM_001202555.2	TGGACCMTTACCATAACTATTTCAAGAGA AA TAGTTATGGTAATTGGTCCTTTTTTC	TCGAGAAAAAAGGACCAATTACCATAACTATT TCTCTTGAAMTAGTTATGGTAATTGGTCCA
WNTJA shRNA 1	NM_033131.4	TGTAGCGAGGACATCGAGTTTGTTCAAGAGAC AAACTCGATGTCCTCGCTACTTTTTTC	TCGAGAAAAAAGTAGCGAGGACATCGAGTTT GTCTCTTGAACAAACTCGATGTCCTCGCTACA
WNTJA shRNA 2	NM_033131.4	TGAACTACGTGGAGATCATGCTTCAAGAGAGC ATGATCTCCACGTAGTTCCTTTTTTC	TCGAGAAAAAAGGAACTACGTGGAGATCATG CCTCTTGAACATGATCTCCACGTAGTTCCA
lk:atenin shRNA 1	NM_001904.4	TATCTGTCTGCTCTAGTAATAATTCAAGAGATT ATTACTAGAGCAGACAGATTTTTTTC	TCGAGAAAAAAATCTGTCTGCTCTAGTAATAAT CTCTTGAATTATTACTAGAGCAGACAGATA
lk:atenin shRNA 2	NM_001904.4	TTCTMCCTCACTTGCMTAATTTCAAGAGAAT TATTGCAAGTGAGGTTAGATTTTTTC	TCGAGAAAAAATCTAACCTCACTTGCAATAATT CTCTTGAAATTATTGCAAGTGAGGTTAGAA
Caveolin-1 shRNA1	NM_001753.5	TACCTTCACTGTGACGAAATATTCAAGAGA TATTTCGTCACAGTGAAGGTGTTTTTTC	TCGAGAAAAAACACCTTCACTGTGACGAAATA TCTCTTGAATATTTCGTCACAGTGAAGGTGA
caveolin-1 shRNA2	NM_001753.5	TACCTTCACTGTGACGAAATATTCAAGA GA TATTTCGTCACAGTGAAGGTG TTTTTTC	TCGAGAAAAAAATCAACTTGCAGAAAGAAATA TCTCTTGAATATTTCTTTCTGCAAGTTGATA
Clathrin shRNA1	NIII_004859.4	TTGACTATGGAGTCTGACAAATTTCAAGAGA ATTTGTCAGACTCCATAGTCA TTTTTTC	TCGAGAAAAAATGACTATGGAGTCTGACAAAT TCTCTTGAAATTTGTCAGACTCCATAGTCAA
Clathrin shRNA2	NM_004859.4	TACTATGGAGTCTGACAAATTTTCAAGAGA AATTTGTCAGACTCCATAGTC TTTTTTC	TCGAGAAAAAAGACTATGGAGTCTGACAAATT TCTCTTGAAAATTTGTCAGACTCCATAGTCA
Firefly luciferase shRNA1	M15077.1	TGCCCTGGTTCCTGGAACMTTTTCAAGAGA MTTGTTCCAGGAACCAGGGCTTTTTTC	TCGAGAAAAAAGCCCTGGTTCCTGGAACAATT TCTCTTGAAAATTGTTCCAGGAACCAGGGCA
Firefly luciferase shRNA2	M15077.1	TTGAGTATTTCTGTCTGATTTTTCAAGAGA AATCAGACAGAAATACTCAC TTTTTTC	TCGAGAAAAAAGTGAGTATTTCTGTCTGATTTT CTCTTGAAMTCAGACAGAAATACTCACA

### 2.15 Confirming the specificity of shRNA experiments

To confirm the shRNA knockdown efficiencies in specific experiments, more than one shRNA was used. The knockdown experiments were confirmed by comparing the knockdown effects of shRNAs for CDS either with those of NCDS (as proper negative controls) or with rescue of the observed shRNA-mediated knockdown phenotype by expression of a resistant form of the targeted mRNA. This was done: 1) by transfecting the cells with specific shRNAs for the CDS of the target gene, or 2) by co-transfecting the shRNA (CDS) for the target gene with or without corresponding cDNA transfection, or 3) by the indicated shRNA-mediated knockdown and corresponding KI gene transfection. Total cell lysates were examined by Western blot analysis for the indicated proteins, and for *β*-tubulin or β-actin (as internal standards). In some cases Total mRNAs were analyzed for the indicated mRNAs by QPCR.

### 2.16 Chromatin immunoprecipitation assay

The chromatin immunoprecipitation (ChIP) assay was done using the ChIP assay kit (Upstate Biotechnology) following the manufacturer’s directions as described (44). A ChIP assay was done with chromatin from SW480-FR CICs using anti-CD44v6 antibody. The immunoprecipitated DNA was amplified using ChIP primers in PCR using Taq polymerase and subcloned in the TA vector (Invitrogen). The reaction mixtures containing clones were transformed in DHα competent bacteria. Plasmids were prepared from randomly selected colonies. Plasmid DNAs from 11 clones were analyzed by M13 sequencing primers. Computer-based analysis of these DNA sequences revealed the presence of various consensus-binding sites for common transcription factors (see **Table 5**). QPCR analyses showed the expressions of these 11 transcription factors in CICs of SW480-FR cells.

**Table 5:** Cis-sequences bourd by CD44v6.

Transcription factors	Accession number	Abundance/11 clones
P300	NM 001429.4	11/11
STATs	NM 139276.3	11/11
TCF4	NM_001083962.2	9/11
cMyc	NM 002467.6	9/11
Snai11	NM 005985.4	8/11
Twist1	NM_000474.4	8/11
Oct4	NM_002701.6	8/11
SOX2	NM 003106	8/11
Nanog	NM 024865.4	8/11
YB-1	NM_004559.5	7/11
NFkB	NM_003998.4	7/11
E2F1	NM 005225.3	7/11
RUNX2	NM 001024630.4	7/11
AP-1	NM_002228.4	7/11
Pgp (MDR1)	NM 001348945	7/11
C/EBP	NM 001806.4	7/11
Notch1	NM_017617.5	7/11

ChiP assay was performed with chromatin from SW480-FR CICs using CD44v6 antibody. The immunoprecipitated DNA-Chromatin complex was amplified by PCR and subcloned. A total of 11 clones were sequenced. Blast analysis revealed the presence of various cis- binding sites for sternness/drug resistance related transcription factors in these DNA sequences.

For ChIP PCR analysis in **Figures 11**, **12**, nuclear fractions (after crosslinking with formaldehyde) from SW480-S and SW480-FR cells were immunoprecipitated with 5 μg of anti-*β*-catenin or TCF4 antibodies, or with CD44v6 antibody, or with 1 μg of normal mouse IgG for 3 hours. Chromosomal DNAs were purified and analyzed using semi-quantitative PCR to detect the MDR1, and for CD44v6 promoter regions, and for *MDR1* and *CD44* promoters (**Figures 11**, **12**). SW480-FR cells were transfected with or without either NT sh, or CD44v6 sh1, or *β*-catenin sh1 for 48 hours. Nuclear *β*-catenin-associated chromatins were immunoprecipitated with *β*-catenin or CD44v6 antibodies for 3 hours. Chromosomal DNAs were purified and analyzed using QPCR with primers for TCF4 sites of MDR1 to detect the MDR1 promoter regions. Similarly, SW480-FR cells were transfected with or without non-targeted (NT) and **β*-catenin* small hairpin RNA (shRNA) sequences, or with dominant negative TCF4 (TCF4-DN) constructs for 48 hours. Nuclear TCF4-associated chromatins were immunoprecipitated with *β*-catenin, or CD44v6 antibodies for 3 hours. Chromosomal DNAs were purified and analyzed using QPCR with primers for TCF4 sites of CD44v6 to detect the *CD44* promoter regions. Control IgGs were used as negative controls for immunoprecipitation. Chromatin inputs were used as loading controls for PCR. The primers used for ChIP PCR experiments studies are presented in **Table 6**.

**Table 6:** ChiP PCR primers for MDR1and CD44v6 promoters.

Genes	Accession number	Primers	
		Forward sequence (5'-3')	Reverse sequence (5' 3')
**MDR1 (A)** **[-644-(-447)]**	Nlil_001348945	TAGGTCTTTCCACTAAAGTC	AGAGGACTTCACACTATCCA
**MDR1 (B) [-1218-(-980)]**	Nl.1_001348945	TTTCTTTCATTCCATTTATC	AAGTCTTCATATCCATATAA
**MDR1 (C) [-1301-(-1056)]**	Nl.1_001348945	AATGTAAGAATTTAAAATGC	CTTTGAAAAGGCTAGGAGAA
**CD44v6 (A) [-1618-(-1370)]**	NIII_001202555.2	AGAAGTCCTGGCATGGTTCC	TCTTCAGGGGAAGCCTTTTGA
**CD44v6 (B) [-1997-(-1793)]**	Nlii_001202555.2	GGATGACTTACTTGTCCCTGT	ACTCACAAGCAGGCCATTACCA

### 2.17 *In vivo* tumorigenic potential of CICs

All animal studies described were approved by the Institutional Animal Care and Use Committee (IACUC) at the Medical University of South Carolina (IACUC # -2017-00250; approval date: 2019/03/14-2021/03/29). Procedures for animal studies were conducted in accordance with the National Institutes of Health Guide for the Care and Use of Animals. For studies of subcutaneous tumors, 2 × 10^3^ or 5 x 10^5^ NON-CICs, or 5 x 10^5^ unsorted bulk tumor cells from a xenograft derived from the patient tissues (PD-FR), or from SW480-FR cells, were suspended in Matrigel and then implanted in 25 mice/cell types, 5 mice per week (Wk) in 6-wk-old female NSG mice (for PD-FR cells) or in SCID mice (for SW480-FR cells) that were obtained from The Jackson Laboratory. Tumors were monitored, and after 2 weeks from the first tumor growth, every 2 weeks, 5 mice were sacrificed, and tumors were removed and weighed to evaluate the tumor development (**Figures 4B**, **4D-G**).

### 2.18 Biotin labelled receptor internalization assays

For cell surface protein labelling, cells were treated in the presence or absence of FOLFOX or WNT3A conditioned media at 37^0^ C for the times indicated and washed three times with ice-cold phosphate-buffered saline (PBS; pH 8.0) to remove any contaminating proteins. Cells were biotinylated using Sulfo-NHS-SS-Biotin (21331, EZ-Link™ Sulfo-NHS-SS-Biotin, ThermoFisher Scientific). Cells from 70–80% confluent cultures (2.5 x 10^7^ cells/ml) were resuspended in PBS, and cell surfaces were biotinylated following the manufacture’s instruction. Cell lysates were prepared in lysis buffer, and biotinylated proteins were precipitated using streptavidin beads from equal amounts of cell lysates. Precipitates were washed three times with cell lysis buffer and analyzed by SDS–PAGE and immunoblotting with appropriate antibodies.

For internalization assays, cell surface proteins were biotin-labelled as described above at room temperature for 1 hour, followed by treatment with or without WNT3A for the indicated times at 37°C. Following stimulation, cells were incubated with 0.1 M glycine in PBS for 30 min at 4°C to quench the unreacted biotin. Surface-retained biotin was removed using reduced glutathione (60 mM glutathione, 0.83 M NaCl), with 0.83 M NaOH and 1% bovine serum albumin (BSA) added before use for two 30-min incubations, followed by ice-cold PBS washes four times. Cells were collected and lysed, and biotinylated proteins isolated using streptavidin beads from equal amounts of cell lysates. The amounts of receptor bound to beads were determined by SDS–PAGE and immunoblot analysis.

### 2.19 Drug Efflux and Retention Assays

[^14^C] Oxaliplatin Efflux/Retention in SW480-S and SW480-FR cells by FOLFOX induced hyaluronan-CD44v6-mediated ankyrin function were analyzed. For drug retention (105), exponentially grown SW480-S and SW480-FR tumor cells (transfected with indicated constructs or with shRNAs) were harvested by trypsinization. The single cell suspensions were plated into tissue culture plates and incubated for 24 hours for attachment. The cells were then washed three times with PBS and incubated for 24 hours with 0.2 µM oxaliplatin containing 300 dpm (2.16 pmole) [^14^C] oxaliplatin (77.6 µCi/mmole). The cells were then washed to remove free radioactive oxaliplatin and incubated in drug-free medium containing 1 x FOLFOX or WNT3A (20 mg/ml) or no FOLFOX, or no WNT3A, or CD44v6shRNA, or CD44_D67_ construct transfection for 48 hours prior to treatment with or without FOLFOX or WNT3A for 2 hours. At the end of treatment, cells were harvested, washed, and cell numbers were measured with a coulter counter. Radioactivity associated with cells (indicated as intracellular drug retention) was then measured by a liquid scintillation counter. Radioactive [^14^C] oxaliplatin in vector control cells was used as 100% (**Figure 11A**).

### 2.20 Statistics

A two-tailed Student’s t-Test was used to compare mean values between sensitive and resistant cells using the following parameters: mean ΔΔCT values for QPCR; mean colony number for soft agar growth assays; mean densitometry values for QPCR and WB; mean percentage of cell viability assay (CellTiter-Glo) and FACS analysis; mean luminescence for ATP activity in cell growth; Caspase Glow assays in Apoptosis measurements; and mean tumor weight in xenograft studies. Chi-squared analysis was done to compare incidences between sensitive and resistant cells for the following assays: number of positive wells containing tumor spheres in sphere formation assays; and number of mice developing tumors in xenograft studies. For experiments involving three or more groups, statistical significance was calculated with GraphPad Prism Software (version 8) using a 1-way or 2-way ANOVA with a Bonferroni’s posttest, Student’s *t* test, or log-rank (Mantel-Cox) test where appropriate (Graph-Pad Software Inc.). Data are presented as the mean ± SD.

### 2.21 Ethics statement

The animal study was approved by the Institutional Animal Care and Use Committee (IACUC) at the Medical University of South Carolina (MUSC). Procedures for animal studies were conducted in accordance with the National Institutes of Health Guide for the Care and Use of Animals IACUC-2017-00250 (approval date: 2019/03/14-2021/03/29).

## 3 Results

### 3.1 Upregulations of CD44v6 and active *β*-catenin contribute to acquired chemoresistance in colon tumor cells

In order to determine the mechanism of FOLFOX resistance in CRC, a cellular model of FOLFOX resistance was developed. Seven CRC cell lines, including the pre-neoplastic APC10.1 cells derived from the APCMin/+ mouse (31, 106), were screened for CD44v6 expression, and SW480, WIDR, LOVO, and HT29 cells that exhibited lower steady-state expressions of CD44v6 were selected (**Figure 1A**). In order to determine the mechanism of resistance to FOLFOX in CRC cells, FOLFOX-resistant (FR) CRC cell clones were established using serially escalated doses of FOLFOX (1-5 × FOLFOX; [1 × FOLFOX = IC_50_ of 5-FU + IC_50_ OXA + 1 µM leucovorin]) in parent sensitive CRC cells (SW480-S, WIDR-S, LOVO-S, and HT29-S) (for details, see Method section). To determine the mechanism of FOLFOX resistance in CRC, ex vivo cultures were established from patient-derived (PD) biopsies collected from 5-FU resistant (PD-5FUR), Oxaliplatin (PD-OXAR), and FOLFOX (PD-FR) tumor specimens and from subcutaneous (SQ) tumor samples derived from our FOLFOX resistant (FR) cell clones. Next, the IC_50_ concentrations of drug-values of 5-FU and OXA for inhibiting SW480, WIDR, HT29 and LOVO CRC cell growth were assessed by a cell viability ATP based assay (Cell Titer-Glo) in the presence of increasing concentrations of these drugs (5-FU, OXA, and FR). **Figure 1B** shows the IC_50_ values of 4 sensitive cell lines (SW480-S, WIDR-S, LOVO-S, and HT29-S) treated with 5-FU or OXA with average values ~49-63 µg/ml for 5-FU and ~5-10 µg/ml for OXA treatments. **Figures 1C-E** show the IC_50_ values of sensitive SW480-S cells compared with SW480-5FU resistant (SW480-5FUR) cells, SW480-OXAR resistant (SW480-OXAR) cells, or SW480-FOLFOX resistant (SW480-FR) cells. In each case the resistant cell lines have 3-5 fold higher IC_50_ values compared to sensitive cells.

While 5-FU, OXA and FOLFOX have been associated with increased CD44v6 mRNA expression in CRC cells (5, 6), their stimulating actions to attain chemoresistance in CICs have not yet been clearly identified. To examine the effects of 5-FU, OXA, and FOLFOX on the regulation of chemoresistance by CD44v6 signaling, we first investigated CD44 variants expressions in sensitive cells and compared their expressions with those of 5-FUR, OXAR, and FR cells of SW480. A specific primer pair was used to amplify the CD44v6 variant by QRT-PCR in these sensitive and drug resistant cells of SW480. Results in **Figure 1C-E** demonstrate that basal CD44v6 expression was very low in SW480-S cells but significantly increased with resistance to chemotherapeutics (5-FU, OXA or FOLFOX). The basal expression of CD44v6 in these cells increased in the order of SW480-S < SW480-FR < SW480-OXAR < SW480-5-FR (**Figure 1F**). Similar results were found in WIDR, HT29, and LOVO CRC cells (data not shown).

We evaluated the kinetics of CD44v6 induction in SW480-S cells upon exposure to 1x FOLFOX. To determine whether FOLFOX resistance is associated with CD44v6, the expression profiles of CD44 variants in PD-FR, PD-OXAR and PD-5FUR cells were monitored by quantitative RT–PCR using distinct sets of primers. See the schematic diagram of the CD44 gene in **Figure 1G**. Sets of CD44 variants were detected using a series of forward 5’ primers that were made to base-pair with v3, v4, v5, v6, v7, v8, v9 and v10 exons independently and with one 3’ primer from the constitutive constant exon 7 (c7) (reverse primer) (shown in **Figure 1G**). Primers are presented in **Table 1** in Methods. In addition, the CD44s standard form having no alternate splicing was detected using primers that base-pair to the constitutive constant exons 5 and 7 of CD44. Exon v6 was expressed together with exons v6–v8 and as an independent isoform (**Figure 1H**). The v6-v8 variants were detected using a 3′ primer from c7 (reverse primer) of CD44 and two distinct 5′-primers (forward primers) complementing to v6 and v8 exons of CD44, respectively (**Figure 1G**). The v6 primers and CD44s primers each principally amplified a single product (**Figure 1H**). The C5v6v8 primer gave rise to two alternately spliced variants of CD44 containing (1): variant exons v6 and v8 (illustrated as v6–v8); and (2) variant exon v8 (shown as v8), all joined to the 3′-constitutive exon C7 (**Figure 1I**). Although RT-PCR results showed that PD-FR, PD-OXAR and PD-5FUR expressed similar CD44 isoforms (**Figure 1H**), PD-FR specimens also express low-molecular-weight isoforms detected by the RT-PCR analysis with primers v3, v5, v6 and v9 (**Figure 1H**). Comparative analysis of matched colorectal cancer specimens from patients after cytotoxic treatment revealed a significant increase in *de novo* CD44v6 transcript across all drug resistant specimens (**Figure 1H**).

We focused on CD44v6 signaling in this study. To characterize specifically the CD44v6 transcript variant, further RT-PCR analysis was done using a forward primer that base pairs with both the v6 and C5 exons, and a reverse primer that base pairs with the C7 exon. RT-PCR results showed that PD-FR, PD-OXA and PD-5-FU cells predominantly expressed the C5v6v8C7 (v6-v8) and C5v6C7 (v6) isoforms (**Figure 1I**). Therefore, we concluded that C5v6v8C7 and C5v6C7 isoforms are unique to chemo resistant cells derived from patients who are resistant to 5-FU, or OXA, or FOLFOX (**Figure 1I**). No changes in CD44s were observed (**Figure 1H**). Overall, our data (**Figures 1F, H, I**) indicate that in patient tumor derived cells, FOLFOX and its components 5-FU and OXA considerably and distinctively induced CD44v6 transcript expression, which could interact with various cellular targets and offer one of the fundamental mechanisms for the drug resistance in CRC cells.

**Figure 2:**
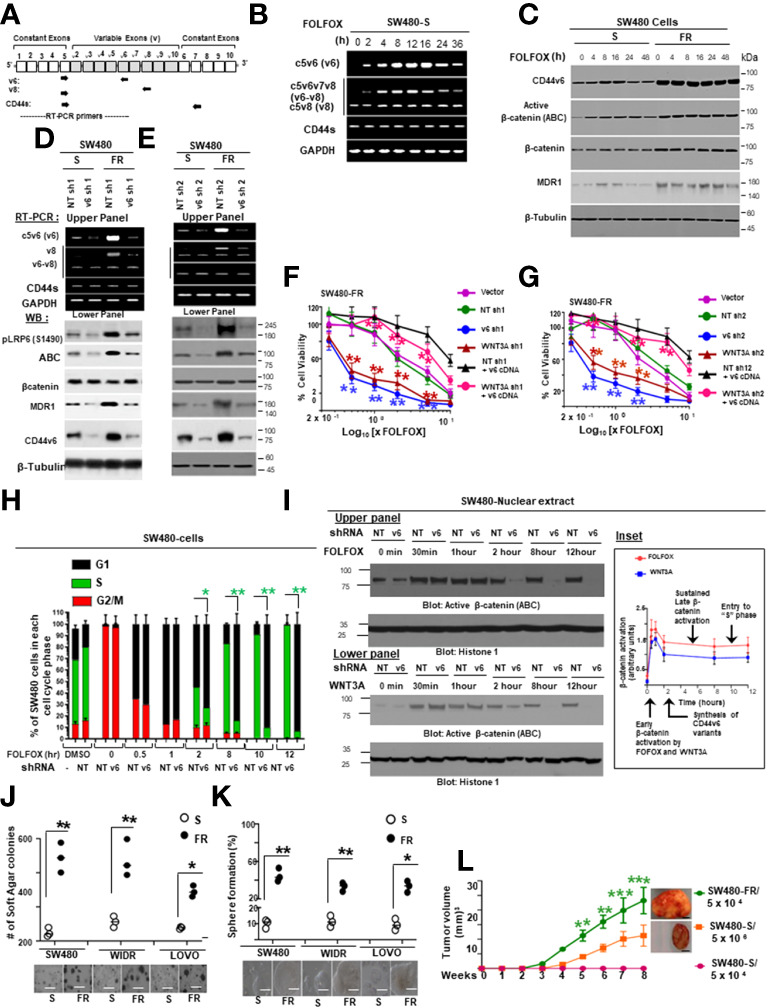
FOLFOX induced CD44v6 expression establishes FOLFOX (FR) resistant colorectal cancer cells (CRCs). **(A)**, Schematic illustration of the CD44 gene. Both constitutive (c) and variable (v) exons are represented. The PCR primers used to amplify CD44 variable and standard isoforms are shown as arrows and the PCR products are depicted in panel **(B)** experiments. **(B)**, Serum-starved SW480-S cells were stimulated with 1 x FOLFOX (50 µg/ml 5-flurouracil + 10 µM oxaliplatin + 1 µM leucovorin) at the indicated time periods. The mRNA expressions show that primers for v6 isoforms generate v6 and v6-v8 PCR products. The primers for v8 and the standard isoform of CD44 primarily generate one product. **(C)**, Western blots are shown for antibodies that recognize either CD44v6, the active hypo phosphorylated *β*-catenin (Active *β*-catenin (ABC)), *β*-catenin, MDR1 or *β*-tubulin in sensitive (S) and FR clones of SW480 cells following stimulation with 1x FOLFOX for 4-48 hours. **(D, E)**, Upper panels: Semi-quantitative RT-PCR analyses are shown for CD44 variants in SW480-S and SW480-FR cells transfected with pSicoR-non targeted shRNA1 (NT sh1) or pSicoR-v6 shRNA1 (v6 sh1) **(D)**, and NT sh2 or v6 sh2 **(E)** for 72 hours followed by FOLFOX stimulation for another 12 hours. Lower Panels: Western blot analyses are shown for p-LRP6 (S1490), active ABC, *β*-catenin, MDR1, or *β*-tubulin following 1 x FOLFOX stimulation for 12 hours in cells transfected with NT sh1 or v6 sh1. **(F)**, Effects of CD44v6 shRNA1 and WNT3A shRNA1 knockdown of CD44v6 and WNT3A respectively on the viability of SW480-FR cells in presence of v6 cDNA that were treated with various concentrations of FOLFOX. An ATP based assay (CellTiter-Glo) measured cell viability compared with vector transfectant without FOLFOX treatment as 100%. Error bars represent calculated SDs (n = 3). **(G)** Same experiments as in F carried out in presence of CD44v6 shRNA2 and WNT3A shRNA2. **(H)** Sixteen hours after release from G2/M phase by nocodazole treatment, when 97% of the cells were in G1 phase (cell cycle analyzed by flow cytometry) the cells were transfected with either non-targeted (Control) or CD44v6 shRNA. Twenty four hours after transfection, the cells were incubated in low serum medium (0.5% serum) with 1 x FOLFOX for different time periods to re-enter the cell cycle. Cell cycle analysis were carried out in these cells. Samples were analyzed through a time course of 12 hours. Bar graph summarizing the flow cytometry cell cycle profile analysis of SW480 cells. Error bars represent ± SEM of five independent experiments. **(I)** Upper and lower panels: Western blot for the activated *β*-catenin accumulation in nuclear fraction in G2/M arrested and 1 x FOLFOX or 20 ng/ml WNT3A stimulated SW480-cells which were previously transfected with NT shRNA or v6 shRNA for 24 hours. These cells were collected at indicated times after 1 x FOLFOX or WNT3A -stimulation. Inset: A model for sustained WNT3A signaling and G1–S transition, dependent on CD44 v6 variants (details are in the text). **(J)**, Anchorage-independent growth in soft agar is shown for SW480-FR, WIDR-FR and LOVO-FR cells and compared with their sensitive (S) pairs. Scale bars, 100 μm. **(K)**, Tumor-sphere formation assays were done for the SW480-FR, WIDR-FR and LOVO-FR cells and compared with their sensitive (S) pairs. Scale bars, 100 μm. **(L)**, Tumor formation is shown in nude mice injected with either 5 x 10^4^ SW480-FR cells, or 5 x 10^4^ SW480-S cells, or 1 x 10^6^ SW480-S cells. SW480-FR cells formed tumor nodules in all injected mice (7/7). Starting at week 3, SW480-R (5 x 10^4^) cells induce tumor nodules whereas SW480-S (5 x 10^6^) cells induced much smaller tumor nodules starting a week later than the SW480-FR cells (7/7 mice). SW480-S (5 x 10^4^) cells were unable to induce tumors. Growth curves are shown for these xenograft tumors in immunocompromised mice. Data are presented as Mean ± SD from n = 3-6 replicates in three independent experiments. All WBs, FACS data, semi quantitative RT-PCR data are representative of three experiments **(F, G)**, *P < 0.05, **P < 0.01 were considered significant, percent cell viability in WNT3A shRNA and CD44v6 shRNA transfected cells compared with vector control and NT shRNA transfected. *P < 0.05, **P < 0.01 were considered significant, percent cell viability in WNT3A shRNA + v6 cDNA transfected cells compared with WNT3 shRNA transfected cell. Student’s t-test was used to assess the significance. **(H)**, *P < 0.05, **P < 0.01 were considered significant, percent cells in S phase in CD44v6 shRNA transfected cells compared with NT shRNA transfected cell. **(J–L)** *P < 0.05, **P < 0.01, ***P < 0.001 were considered significant. Soft agar colonies, tumor sphere growth, and SQ tumor growth of SW480-FR cells were compared with SW480-S cells. Tumor growth kinetics data in (L) (n = 7) represent mean +/- SD, from at least 3 independent experiments.

To determine the effects of the expression profiles of CD44 variants, SW480 cells were examined after stimulation with FOLFOX by exon-specific reverse transcription-PCR (RT-PCR). The expression levels of CD44v6 transcripts were monitored by quantitative RT–PCR using distinct sets of primers. See the schematic diagram of the CD44 gene in **Figure 2A**. A set of variants were detected using a 5’ primer from a constitutive exon C5 and two different 3’ primers complementary to either the v6 or v8 exon, respectively (primers are shown in **Table 2**). In addition, the standard form of CD44 (CD44s) was detected using primers that base-pair to the constitutive exons C5 and C6. The v6 primer and standard primer predominantly amplified a single product C5v6 (CD44v6 [v6]) (**Figure 2B**). The v8 primer amplified two spliced variants containing (1) C5v6v7v8 (referred as CD44v6-v8 [v6-v8]), and (2) C5v8 (referred as CD44v8 [v8]) all joined to the c5 constitutive exon (**Figure 2A**). All products were confirmed by DNA sequencing. As shown in **Figure 2B**, following 24 hours of serum starvation, the relative expression levels of CD44 variants were low. Stimulation of these cells with 1 x FOLFOX upregulated the v6 gene transcript that peaked between 4 and 16 hours and returned to basal levels at 24–36 hours likely due to the exhaustion of FOLFOX within the media (**Figure 2B**).

Next, we found that FOLFOX-resistant (FR) cells express constitutively high levels of CD44v6 and hypo-phosphorylated, active *β*-catenin (ABC), as a read-out for WNT pathway activity, and for increased MDR1 protein expression, as a read-out for drug resistance compared to sensitive cells (**Figure 2C**). In contrast, sensitive cells have low basal levels of these proteins that transiently increase following FOLFOX treatment but return to basal levels following drug withdrawal (**Figure 2C**). However, this induction in sensitive cells fails to reach the constitutive level as observed in resistant cells (time 0, **Figure 2C**). These results of high expression of CD44v6 in SW480-FR cells (**Figures 1F, H**, **2B, C**) were consistent with high expression of the CD44v6 transcript in PD-FR cultures (**Figures 1H, I**). To further validate that active *β*-catenin and MDR1 expressions in CRC cells are correlated with CD44v6 expression, we evaluated the effects of FOLFOX on expression of these proteins in SW480-S and SW480-FR cells following FOLFOX treatment with or without CD44v6 shRNA1, and CD44v6 shRNA2 (**Figures 2D, E**). These two sets of shRNAs were used to confirm that the effects of FOLFOX on CD44v6 function are specific to v6. Knockdown of CD44v6 in both FOLFOX-treated SW480-S and SW480-FR cells down-regulated expressions of pLRP6, active *β*-catenin, and MDR1 and inhibited the FOLFOX-induction of v6-containing variants but not of standard or v8 variants (**Figures 2D, E**). To further test that CD44v6-regulated WNT/*β*-catenin signaling affects resistance in response to FOLFOX, FOLFOX resistant SW480-FR tumor cell viability was assayed using different doses of FOLFOX in the presence or absence of two different sets of shRNAs (shRNA1 and shRNA2) targeted to CD44v6 and WNT3A (CD44v6 shRNA1/2, or *WNT3A* shRNA1/2, and CD44v6 shRNA1/2 plus pCD44v6 cDNA) (**Figures 2F, G**). A similar experiment was done with a second set of *shRNAs* for CD44v6 and *WNT3A* to confirm that the effects of CD44v6 and *WNT3A* are specific for FOLFOX stimulated colon tumor resistance (**Figures 2G**). WNT3A shRNA alone inhibited tumor cell proliferation to nearly the same extent as v6 shRNA at the highest concentration of FOLFOX treatment (**Figures 2F, G**). However, *WNT3A* shRNA1, or *WNT3A* shRNA2 combined with v6 cDNA overexpression nearly eliminated WNT3A shRNA-mediated inhibition of FOLFOX resistance compared to v6 cDNA + NT shRNA1, or v6 cDNA + NT shRNA2 groups in SW480-FR cells (**Figures 2F, G**, magenta lines compared to black lines). This suggests that WNT3A-CD44v6 interaction promotes resistance to FOLFOX induced death in the presence of the chemotherapeutic drug FOLFOX in CRC cells (**Figures 2F, G**). Overall, these data indicate that FOLFOX transiently upregulates CD44v6, active *β*-catenin and MDR1 expression in sensitive cells, while these proteins are already constitutively high in FR cells (as seen in **Figure 2C**) and facilitate FOLFOX resistance (**Figures 2F, G**). **Supplemental Figure1A-C** show the validation of WNT3A shRNA, CD44v6 shRNA, and v6 cDNA expression vectors. Our results (**Figures 2B, C, F, G**) also show that two sets of shRNAs display similar results confirming that this effect on FOLFOX resistance was specific to CD44v6 and WNT3A/CD44v6 signaling and not an off-target effect of using CD44v6 shRNA and WNT3A shRNA.

Next, results in **Supplemental Figure 1D** showed that knock down of WNT3A in SW480-FR cells inhibited the up-regulation of CD44v6 variants. These data indicated that WNT3A signaling may be required for FOLFOX-induced alternative splicing of CD44. These results (**Figures 2F, G**, and **Supplemental Figure 1C**) provide strong support for a positive feedback loop in which specific v6 isoform-dependent activation of WNT3A signaling in response to FOLFOX results in additional synthesis of these CD44v6 isoforms. The results confirm that the positive feedback loop altering WNT3A signaling could lead to long lasting changes in cellular properties such as cell cycle progression. A previous study showed that cytoplasmic and nuclear *β*-catenin are dynamically regulated during the cell cycle and increased during S phase (107). To investigate the role of the positive feedback loop between CD44v6 and WNT3A signaling in cell cycle progression, we determined the time course of active *β*-catenin (ABC) expression, and the cell cycle progression of quiescent cells into S phase in SW480 cells treated with CD44v6 shRNA followed by treatment with WNT3A or FOLFOX treatment for various time periods (**Figures 2H, I**, and **Supplemental Figure 1D**). After 2 hours of FOLFOX or WNT3A stimulation, 35-43% of the cells entered S phase in NT shRNA transfected cells compared with 15-23% of CD44v6 shRNA transfected SW480 cells treated with FOLFOX, and WNT3A respectively (**Figure 2H**, and **Supplemental Figure1D**). Upon WNT3A, or FOLFOX stimulation, WNT3A either by itself, or through FOLFOX, induced an initial burst of nuclear *β*-catenin activation independent of CD44v6. This activated signal was rapidly downregulated by CD44v6 shRNA after 2 hours. As shown in **Figure 2H** and **Supplemental Figure 1D**, ∼90% of the NT shRNA treated cells had entered S phase after 10-12 hours of treatment with FOLFOX and WNT3A. In contrast, only ∼7-12% of the CD44v6 shRNA-treated cells had progressed into S phase at 12 hours of stimulation with FOLFOX and WNT3A, indicating an interruption of S-phase access following down-regulation of the CD44v6 isoform. However, these results initiate a positive feedback loop between WNT3A mediated *β*-catenin activation and CD44v6 splicing (**Figures 2H, I**, and **Supplemental Figure 1D**) by stimulating v6-specific CD44 expression, and this could establish a mechanism for persistent activated *β*-catenin signaling in FOLFOX resistant cells. In addition, our FOLFOX resistant cells were generated by repeated exposure of the cells to 1x – 5x FOLFOX, and the resistant clones were maintained in 0.5 x FOLFOX selection pressure. Therefore, the FOLFOX-resistant cells sustain WNT3A production, and the maintenance of WNT3A/*β*-catenin signaling necessary for S phase could constitute a mechanism for a positive feedback loop between *β*-catenin activation and CD44v6. The depletion of this circuit by the use of v6 exon-specific shRNA in our study uncovered new understandings into how regulated alternative splicing can control intracellular *β*-catenin signaling sufficient to drive cell cycle progression in response to FOLFOX induced WNT3A secretion.

To further understand whether resistance to chemotherapeutics has been independently associated with increased CD44v6 variant expression that may be associated with a *β*-catenin/MDR1 pathway, we evaluated stemness in sensitive and FOLFOX resistant (FR) cells of SW480, WIDR and LOVO, by determining their clonogenicity, tumor sphere formation, and their *in vivo* tumor development by implanting FR and sensitive (S) cells in immunocompromised mice. To determine the clonogenicity of these cells *in vitro*, their clonal capacity was measured in a soft agar colony formation assay. Compared to parental S cells, FR cells were able to form increased anchorage-independent growth assessed by formation of large numbers of soft agar colonies (**Figure 2J**). Further, compared with parental S cells, FR cells were able to form significantly greater numbers of tumor-spheres in serum free medium (**Figure 2K**).

Next, to evaluate whether FOLFOX resistant cells increased tumor growth *in vivo* compared to the corresponding sensitive cells, 5 × 10^4^ SW480-FR cells, 5 x10^4^ SW480-S cells and 5 x 10^6^ SW480-S cells, were each implanted into 7 immunocompromised mice in 3 separate experiments. In agreement with the soft agar growth and tumor sphere formation results (**Figures 2J, K**), 5 × 10^4^ FR cells generated tumors in at least 90–100% of immunocompromised mice injected with SW480-FR cells (**Figure 2L**, green, tumor formation = 7/7 mice), whereas 5 × 10^4^ sensitive cells (SW480-S) were not adequate to form tumors (**Figure 2L**, purple, tumor formation = 0/7 mice). However, implantation of 200-fold more sensitive SW480-S cells (5 × 10^6^) initiated tumors in three independent experiments (**Figure 2L**, orange, tumor formation = 6/7 mice). When tumor volumes were examined every day to evaluate the latency, tumors initiated from 5 × 10^4^ SW480-FR cells began to increase at 2 weeks while tumors initiated from 5 × 10^6^ SW480-S cells began to increase later at 3–4 weeks and had much smaller size at 8 weeks compared with 5 × 10^4^ SW480-FR cell-derived sub cutaneous (SQ) tumors (averages 1175 mm^3^ compared to 2400 mm^3^ at 8 weeks, **Figure 2L**). The results from **Figures 2J–L** provide evidence that FOLFOX-resistant FR cells were more tumorigenic *in vitro* and *in vivo* and had greater sphere-forming activity than parental sensitive cells, which are hallmark characteristics of CRC-CICs. This provides evidence that expansion of CICs expressing CD44v6 can have an important role for the acquisition of FOLFOX resistance.

Overall, these results indicate that: 1) CD44v6 has key roles for FOLFOX-induced WNT3A/*β*-catenin/MDR1 activation that is clearly inhibited by CD44v6 shRNA, confirming that FOLFOX might induce WNT ligands to mediate CD44v6-dependent WNT/*β*-catenin signaling, 2) constitutive activation of CD44v6 and WNT3A are necessary for maintaining FOLFOX resistance in CRC cells through a WNT3A-CD44v6-*β*-catenin-MDR1 pathway, and 3) Our data, suggest (**Figure 2H, I**, and **Supplemental Figure 1D**) that upon FOLFOX or WNT3A stimulation, WNT/*β*-catenin signaling generate an early burst of *β*-catenin activation independent of CD44v6 variants. This activation of *β*-catenin is promptly down-regulated by CD44v6 shRNA and the findings suggest that a positive feedback loop between WNT/*β*-catenin signal activation and CD44v6 splicing occurs by stimulating v6-specific CD44 expression.

### 3.2 CD44v6 expression defines highly tumorigenic colorectal cancer-initiating cells

Cancer initiating cells (CICs) have two decisive features: stemness and resistance to conventional chemotherapies, and thus are a hallmark of drug resistance. CICs are considered to remain after chemotherapy to initiate metastasis (108). According to the published data, CD44v6 has important roles in the stemness of CICs (15, 19, 109). Therefore, we investigated whether expression of CD44v6 defines CRC/CIC subpopulations with drug resistance and tumorigenic properties in clinical samples (PD-FR, PD-5FUR and PD-OXAR) isolated from patients who were resistant to several chemotherapeutic drugs as well as in our FOLFOX resistant WIDR, HT29 and SW480 cells.

**Figure 3:**
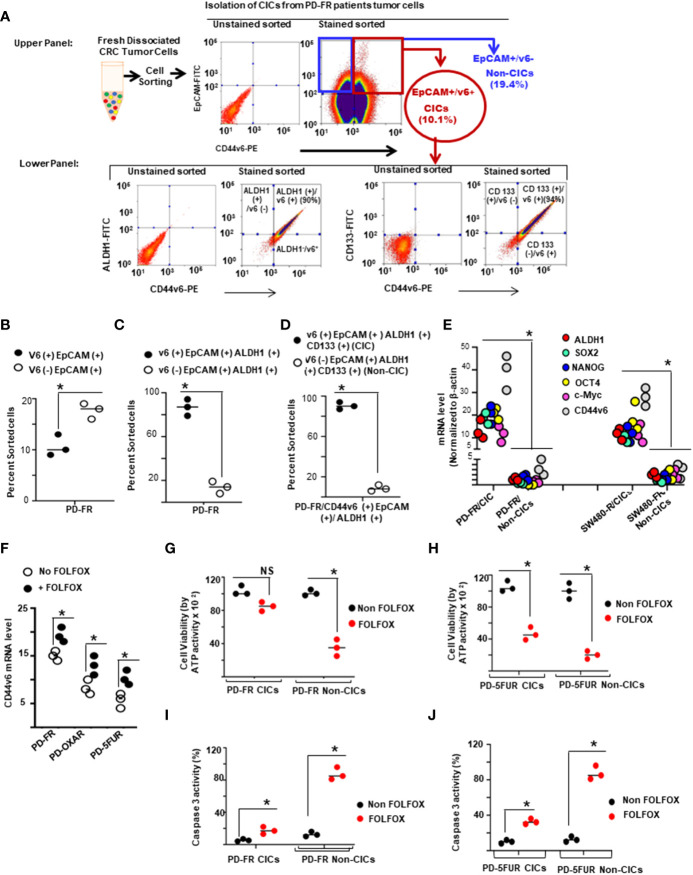
CD44v6 identifies colorectal cancer (CRC) initiating cells (CICs). **(A)**, Single-cell suspensions from patient derived (PD) specimens collected from 5-FU (PD-5FUR), Oxaliplatin (PD-OXAR) and FOLFOX (PD-FR) resistant tumor tissues, and from SW480-FR/subcutaneously (SQ) tumor cells were processed for tumor spheres. Sphere propagated cells were FACS sorted for high expressions of CD44v6-PE. CD44v6 positive (+) populations were sorted using EpCAM (–) FITC and CD44v6-PE. CD44v6 (+)/EpCAM (+) cells from various tumors were cultured separately and grown in fresh medium for 2 weeks. The cells were then subjected to flow cytometric analysis for isolation of CD44v6 (+)/EpCAM (+)/ALDH1 (+)/CD133 (+) (CICs), and for CD44v6 (–)/EpCAM (+)/ALDH1 (+)/CD133 (+) (Non-CICs) using antibodies to ALDH11-FITC, CD133-FITC or CD44v6-PE. **(B–D)**, Percentages of CD44v6 (+) and CD44v6 (–) fractions in EpCAM (+) **(B)**, in EpCAM (+)/ALDH1(+) **(C)**, and in EpCAM (+)/ALDH1 (+)/CD133 (+) **(D)** sorted cells in PD-FR tumor tissues are shown. Henceforth, the CD44v6 (+)/EpCAM (+)/ALDH1 (+)/CD133 (+) cells are identified as CICs, and the CD44v6 (–)/EpCAM (+)/ALDH1 (+)/CD133 (+) cells as Non-CICs (details in Methods). **(E)**, QPCR analyses of CIC-stemness markers (ALDH1, SOX2, OCT4, Nanog, c-Myc and CD44v6) were done on PD-FR CICs, PD-FR Non-CICs, SW480-FR CICs, and SW480-FR Non-CICs isolated from SW480-FR and SW480-S SQ tumor samples. **(F)**, CD44v6 mRNA expressions (by QPCR) are shown in PD-FR, PD-OXAR and PD-5FUR cells treated with or without 1 x FOLFOX for 12 hours. Data are presented as fold change of CD44v6 mRNA expressions relative to adjacent control cells from colon tissue. **(G, H)**, Cell viability of CICs and non-CICs from three independent PD-FR **(G)** and PD-5-FUR **(H)** cultures following treatment with or without FOLFOX were assessed by an ATP based assay (CellTiter-Glo). **(I–J)**, Apoptosis of CICs and non-CICs from three independent PD-FR **(I)** and PD-5-FUR **(J)** cultures following 1 x FOLFOX treatment were assessed by a Caspase 3 ELISA assay. Data are presented as Mean ± SD from n = 3-6 replicates in three independent experiments. All QPCR, and FACS data are representative of three independent experiments **(B–D)**, *P < 0.05, were considered significant, CD44v6 (+) cells were compared with CD44v6 (–) cells. **(E)**, *P < 0.05, were considered significant, expression of stemness associated factors of CICs were compared with Non-CICs. (FH–J), *P < 0.05, were considered significant, FOLFOX treated cells were compared with Non FOLFOX cells.

First, CICs were isolated from the tumor sphere-propagated cells from colorectal human specimens and colorectal xenograft (SQ) tumors by FACS sorting using several of the previously reported candidates (CD44v6, CD133, EpCAM and ALDH1) (19, 43, 110–112). The data in **Figure 3A** (upper panel) show that CD44v6 (+) EpCAM (+) sorted cells (10% of unsorted PD-FR tumor cells, **Figure 3B**) overlapped with CIC markers ALDH1 and CD133 antigen expressions in PD-FR patient tissues (lower panel of **Figure 3A**). The data in **Figures 3C, D** show the percentages of CD44v6(+) and CD44v6 (–) cells in EpCAM (+)/ALDH1 (+), and EpCAM (+)/ALDH1 (+)/CD133 (+) cells with respect to unsorted cells. Hereafter freshly isolated CD44v6 (+)/EpCAM (+)/ALDH1 (+)/CD133 (+) cells from the corresponding sphere-propagated tumor cells will be referred to as CICs, and CD44v6 (–)/EpCAM (+)/ALDH1 (+)/CD133 (+) cells as Non-CICs (**Figure 3D**). Results in **Figure 3E** showed increased CIC-stemness related gene expressions (primers are given in **Table 3** in Methods) in CICs isolated from both PD-FR patient tissues and from SW480-FR/SQ tumors compared to their respective Non-CICs. Overall, the data in **Figures 3A-E** validate that the CICs overexpressing CD44v6 were originated from epithelial and stem cells.

Second, given that CD44v6 and *β*-catenin activation are CRC-CIC markers (19, 113) and that FOLFOX therapy induces CD44v6 associated *β*-catenin-MDR1 signaling (as seen in **Figures 2C-G**), we examined whether CD44v6-*β*-catenin signaling can classify CRC/CICs as a FOLFOX-resistant phenotype. In agreement with the results in our generated FR and S cells of SW480 (as seen in **Figures 1F**, **2B-E**), data in clinically relevant human specimens demonstrated that basal CD44v6 expression was also significantly increased in ex vivo cultures from PD-FR tumor specimens compared to PD-OXAR and PD-5-FUR tumor specimens, and FOLFOX stimulation further increased CD44v6 mRNA expressions in each of the cultures from PD-FR, PD-OXAR and PD-5-FU specimens (**Figure 3F**). Next, the viable cell growth of CICs and Non-CICs derived from our PD-FR and PD-5FUR specimens were compared to FOLFOX treatment (**Figures 3G, H**). The data in **Figure 3G** demonstrate that in a PD-FR tissue, the cell viability of CICs was little or not affected by FOLFOX treatment, whereas Non-CICs displayed an ~2.8-fold reduction in cell viability following FOLFOX therapy that correlated with increased Caspase 3 activation (**Figure 3I**). In PD-5FUR cells, Non-CICs displayed ~2.3 fold more sensitivity to FOLFOX (measured by Caspase 3 activity) compared with CICs from PD-5FUR SQ tumor cells (**Figures 3H, J**). Importantly, PD-5FUR CICs from SQ tumors displayed partial sensitivity to FOLFOX (40%) compared to no sensitivity in PD-FR CICs indicating that our PD-FR CICs are indeed resistant to FOLFOX, whereas PD-5FUR CICs are only partially resistant to FOLFOX (**Figures 3G-J**).

**Figure 4:**
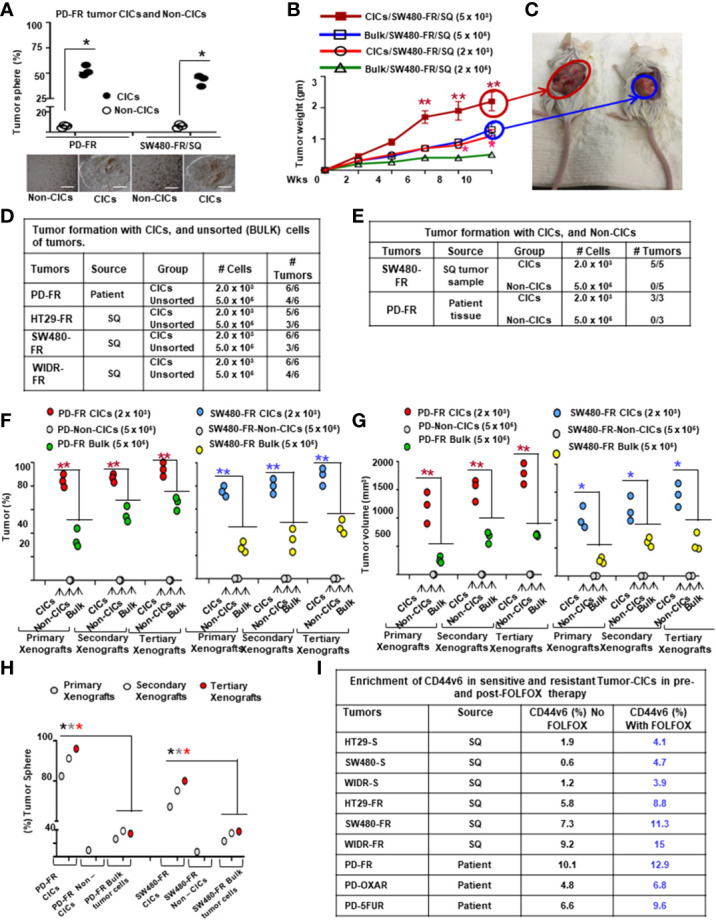
Tumor response to FOLFOX is correlated with enrichment of CD44v6 (+) CICs and resistance of CICs. **(A)**, Percentages of tumor sphere formation of PD-FR CICs and Non-CICs were measured in a sphere-formation assay. Representative pictures of tumors are shown. **(B)**, Implantation of 5 x 10^3^ CICs from SW480-FR (SQ) tumors resuspended in Matrigel were tumorigenic while 100-fold more unsorted cells (Bulk) resuspended in Matrigel were required to generate tumors in four independent implantations. Five mice per group were used. SW480-FR SQ tumor weights following injection of indicated cell numbers from CICs and unsorted (Bulk) tumor cells are shown. **(C)**, A representative image of tumors initiated from **(B)** is shown. **(D)**, FACS sorted 2 x 10^3^ CICs resuspended in Matrigel were tumorigenic while 250-fold more unsorted bulk cells (resuspended in Matrigel) were unable to generate the same capacity of tumorigenesis in four independent specimens. Six mice per group were used for four independent experiments. **(E)**, Numbers of SQ tumors formed by implantations of the indicated numbers of CICs and Non-CICs resuspended in Matrigel that were FACS sorted from the SW480-FR cells and PD-FR cells are shown. Five mice were used per group. **(F)**, FACS sorted CICs (2 x 10^3^), Non-CICs (5 x 10^5^), and the unfractionated bulk tumor cells (5 x 10^5^), from indicated tumor samples were resuspended in Matrigel and implanted in immunocompromised mice. The same cells from the first generation of SQ tumors were further implanted into immunocompromised mice. The experiments were repeated to generate tumors into the third generation of xenograft tumors. Only CICs and the unfractionated bulk tumor cells were capable of inducing tumor formations. Isolation of second and third generation xenograft tumor cells displayed similar results (n = 5 mice; experiments were performed in triplicates). **(G)**, Tumor volumes from the experiment **(F)** were measured in implanted tumors from the indicated CICs, and from the unfractionated bulk tumor cells (n = 5 mice; experiments were performed in triplicates). **(H)**, Percentages of tumor sphere formation in freshly isolated dissociated primary, secondary and tertiary xenograft tumors generated with the indicated CICs, Non-CICs, and unsorted bulk tumor cells from the experiment **(F)** are shown. **(I)**, Enrichments of CICs in bulk cells from three sources – 1) patient derived specimens, 2) SQ tumor samples developed using FR-cells, and 3) the corresponding sensitive pairs, were assessed by FACS analysis for CD44v6 after FOLFOX treatment. Data are representative of four independent human specimens, and of three independent tumor samples from sensitive and FR cells. Data are presented as Mean ± SD from n = 3 replicates in three independent experiments. **(A)** *P < 0.05, were considered significant, tumor sphere growth in PD-FR CICs were compared with Non-CICs. **(B)**, *P < 0.05, **P < 0.01, were considered significant, SQ tumor growth in SW480-FR CICs were compared with bulk tumor from SW480-FR cells. Tumor growth kinetics data in **(B, C)**, n = 6 mice/each group represent mean +/- SD, from at least 3 independent experiments. **(D, E)**, n = 6 mice/each cell types (CIC or unsorted) in each cell types in three independent experiments represent mean +/- SD.**(F, G)**, n = 5 mice/each cell types (CIC, Non-CICs, or unsorted) in three independent experiments represent mean +/- SD, *P < 0.05, **P < 0.01, were considered significant, tumor growth using CICs were compared with tumors from bulk tumor cells. **(H)**, *P < 0.05, *P < 0.05, *P < 0.05, were considered significant for tumor sphere growth from cells from primary, secondary, and tertiary xenografts of CICs compared with bulk cells. Tumor growth kinetics data in L (n = 7) represent mean +/- SD, from at least 3 independent experiments.

Third, we evaluated tumor sphere forming ability between CICs and Non-CICs from PD-FR and SW480-FR/SQ tumor cells. Quantification of the sphere-formation assay demonstrates that CICs have elevated tumor sphere formation efficiency (**Figure 4A**). To establish the effects on CICs in functional assays, we performed SQ tumor growth assays (**Figures 4B, C**) for CICs, Non-CICs and unsorted bulk tumor cells. Importantly, tumors derived from freshly isolated CICs from SW480-FR and PD-FR tumor samples were able to generate larger tumors compared to those induced by unsorted cells in immunocompromised mice (**Figures 4B, C**). When CICs were injected in immune-compromised mice, 80-100% developed tumors compared to 50-65% of mice injected with unsorted (Bulk) cells (**Figure 4D**). Examples of tumor growth in cultures of SW480-FR/CICs and PD-FR/CICs isolated from xenograft tumors, and the inability of Non CICs from these tumors to do so, are shown in **Figures 4B-D**. Moreover, the CICs increased tumor incidence and reduced the latency of tumor formation by PD-FR, HT29-FR, SW480-FR and WID-FR cells with increased tumor sizes in mice implanted with CICs compared to unsorted (Bulk) tumor cells, which required 100-fold more cells compared to CICs (**Figure 4D**). In concordance with results of **Figures 4B, D**, when implanted into immunocompromised mice, despite the higher number of unsorted bulk tumor cells (5 x 10^5^) that were used, tumor formations following injection of purified CICs (2 x 10^3^) were faster and more efficient than tumor formations obtained with the total unsorted bulk cancer cell population (**Figures 4B-D**). Importantly, as high as 5 x 10^5^ Non-CICs from these specimens failed to form any tumors (**Figure 4E**).

Fourth, to investigate whether CICs from patient-derived PD-FR colon tumor cells, and from SW480-FR/SQ tumor cells display long-term tumorigenic potential, we evaluated their ability to generate tumors after serial transplantations. Indeed, injected CICs engrafted and generated tumors that grew rapidly and required the mouse to be sacrificed within 28 days. Similar to the results of **Figures 4D-E**, examples of tumor growth of freshly isolated SW480-FR/SQ/CICs and PD-FR/SQ/CICs, and the inability of Non-CICs from these tumors to do so, are shown in **Figures 4F-G**. Interestingly, despite the presence of CD44v6 (+) cells present in 5 x 10^5^ unsorted bulk cells, tumor formation following implantation of sorted CICs was quicker and more effective than tumor generation from the 5 x 10^5^ unsorted bulk tumor cell population (**Figure 4F**). To determine if the tumorigenic population in CRC is restricted to CD44v6 (+), CICs were evaluated by their ability to generate tumors after serial transplantations in secondary and tertiary xenograft models. To address this issue, 2 x 10^3^ CICs, 5 x 10^5^ Non-CICs, and 5 x 10^5^ unfractionated tumor cells from primary tumor xenografts were transplanted into a secondary xenograft model. The implanted CICs increased tumor incidence, grew rapidly and reduced the latency of tumor formation by CICs with increased tumor size (**Figures 4F, G**). Furthermore, CICs obtained from similar CIC derived secondary xenografts were subsequently transplanted into third generation of xenografts in mice. During the *in vivo* serial transplantation, CICs did not lose their tumorigenic potential but instead increased their long-lasting faster tumor growth (as measured by the tumor volume in **Figure 4G**). In contrast to the data of **Figures 4F, G**, importantly, the tumorigenic potentiality of the Non-CICs was entirely lost in secondary recipients (**Figures 4H**, as well as in 4F), providing evidence that Non-CICs include mainly differentiated nontumorigenic cells whereas tumorigenic colorectal CICs are restricted to the small population of CICs expressing CD44v6 (**Figures 4F-H**). Thus, the CIC population in colon tumors was able to generate serial xenografts showing a nearly unlimited tumor growth potential.

The relative resistance of CICs to chemotherapy (as seen in **Figure 4A-H**) suggests that CICs may be enriched after chemotherapy treatment. Indeed, the CIC immunophenotype (CD44v6 (+)) was increased 1.3-8-fold after chemotherapy treatment (**Figure 4I**). Importantly, the CIC population in the colon tumor tissue can generate serial transplantation derived SQ tumors indicating an essentially unlimited tumorigenic potential of CICs expressing CD44v6 (**Figures 4F–H**). Together, these data collectively support a model in which drug resistant colorectal CICs are confined to the small CD44v6 (+)/EpCAM (+)/ALDH1 (+)/CD133 (+) cell populations isolated from sphere-propagated tumor cells (as seen in **Figure 3D**), which are enriched with FOLFOX therapy (**Figure 4I**) with high tumorigenic potential (**Figures 4B-H**). The tumorigenic potential of CICs was not related to a higher content of CD44v6 (+) CICs in response to FOLFOX therapy as seen in **Figure 4E**, but may be related to *in vivo* selection of a highly tumorigenic subpopulation of CICs (**Figures 4F-H**). These results favor a cell-autonomous relative chemo resistant and virtually unlimited growth potential phenotype of colorectal CICs expressing CD44v6, indicating that CD44v6 can be used as a CIC marker, and as a therapeutic target for CRC.

### 3.3 FOLFOX-induced WNT3A and CD44v6 signaling establishes cell autonomous resistance to conventional FOLFOX chemotherapies in colorectal CICs

**Figure 5:**
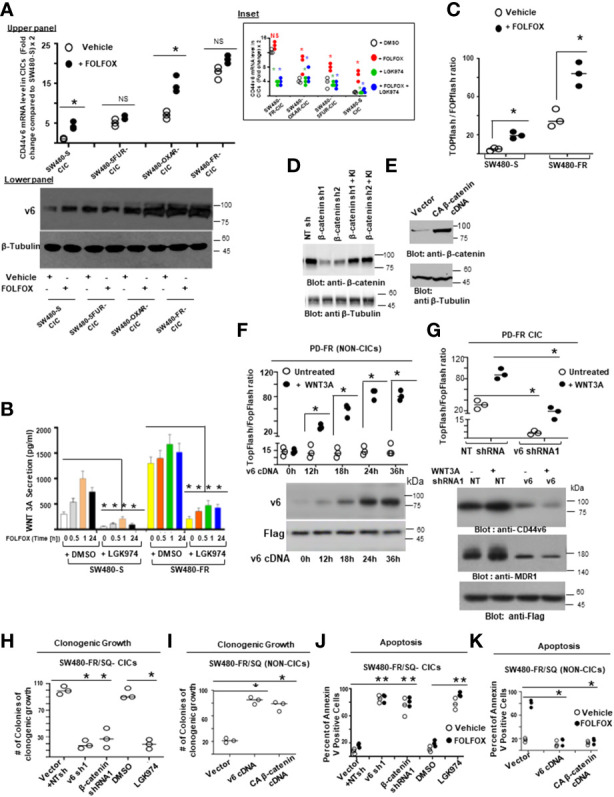
CICs isolated from resistant cells demonstrate resistance to FOLFOX treatment through WNT3A/*β*-catenin signaling. **(A)**, QPCR (Upper panel) and western blot (Lower Panel) data are shown for CD44v6 mRNA (Upper panel) and protein expression (Lower panel) in CICs isolated from SQ tumors of SW480-FR, SW480-OXA, SW-5-FU, and SW480-S cells that were treated with or without either 1 x FOLFOX for 12 hours, Inset: CICs isolated from SQ tumors of SW480-FR, SW480-OXA, SW-5-FU, and SW480-S cells that were treated with either 1 x FOLFOX, or 1.2 ng/ml of WNT inhibitor LGK974 (IC_50,_ for LGK974 in SW480-S and SW480-FR cells are 0.8 ng/ml and 1.15 ng/ml [data not shown]), or 1 x FOLFOX + LGK974 for 12 hours. QPCR analysis was done with total RNA extracted from these treated cells and data are shown for CD44v6 mRNA. **(B)**, Secretion of WNT3A was measured by ELISA in sensitive and FR cells of SW480 after treatment with DMSO. Or 1 x FOLFOX, or 1.2ng/ml of LGK974, or 1 x FOLFOX + LGK974 for the indicated times. **(C)**, Sensitive and FR cells of SW480 were transfected with 50 ng TOPFlash and 50 ng TK-Renilla vectors, or with 50 ng FOPFlash and 50 ng TK-Renilla vectors. The TOPFlash/FOPFlash promoter was activated by treatment with FOLFOX (1x) for 12 hours. Cells were lysed and subjected to luciferase measurements. **(D-E)**, Validations of *β*-catenin shRNAs (*β*-catenin sh1 and *β*-catenin sh2) **(D)** and of constitutively active *β*-actin **(E)** used in the following experiments **(H-K)** were examined. In “D”, the indicated shRNA mediated knockdown and the corresponding knock-in **(KI)** gene transfections were dune as described in Methods. Target proteins were analyzed by WB analysis (*β*-tubulin, internal control). **(F)**, CD44v6 negative PD-FR/NON-CICs were transfected with either TOPFlash and control TK-Renilla vectors, or with FOPFlash and TK-Renilla vectors together with increasing time of incubation with CD44v6 cDNAs. After 48 hours, the cells were stimulated with or without 20 ng/ml WNT3A for the indicated times. Then the cells were lysed and subjected to luciferase measurements (upper panel) or, in parallel, to WB analysis for CD44v6 and Flag. **(G)**, PD-FR CICs were transfected with NT sh1, or with CD44v6 sh1. After 48 hours, cells were analyzed for WNT3A stimulated *β*-catenin/TCF4 promoter luciferase activity as shown in upper panel or, in parallel, to WB analysis with the indicated proteins (lower panel). **(H, J)**, SW480-FR CICs were transfected with NT sh1, or CD44v6 sh1 (v6 sh1), or *β*-catenin sh1, or treated with DMSO, or 1.2 ng/ml of LGK974. 48 hours after the transfections, and 12 hours after the LGK974 treatment, cell growth was assessed by counting colonies in a clonogenic growth assay **(H)**, and apoptosis was assessed by the Annexin V positive stain assay **(J)**. **(I, K)**, SW480-FR Non-CICs were transfected with vector control, v6 cDNA, or CA *β*-catenin cDNA. 48 hours after the transfections, cell growth was assessed by clonogenic growth assay **(I)**, and apoptosis was assessed by the Annexin V positive stain assay **(K)**. Data are presented as Mean ± SD from n = 3-4 replicates in three independent experiments. All WB data are representative of 4 independent experiments. **(A)** *P < 0.05 was considered significant for red asterisks, CD44v6 mRNA levels of FOLFOX treated cells were compared with the DMSO treated cells; *P < 0.05 considered significant for the green and blue asterisks, CD44v6 mRNA levels of 1.2 ng/ml, LGK974 and FOLFOX + LGK974 treated cells were compared with DMSO, or FOLFOX treated controls. **(B)**, *P < 0.05, was considered significant, secreted WNT3A in LGK974 treated cells of SW480-S and SW480-FR were compared with their respective DMSO treated controls. **(C)**, *P < 0.05, was considered significant, FOLFOX treated cells of SW480-S and SW480-FR were compared with their respective DMSO treated controls. **(D-E)**, *P < 0.05, was considered significant, WNT3A treated PD-FR NON-CICs **(D)** and PD-FR CICs **(E)** at various time points were compared with their respective untreated controls. **(F, H)**, *P < 0.05, was considered significant, v6 shRNA1, *β*-catenin shRNA1, and LGK974 treated clonogenic growth **(F)**, and Annexin V positive **(H)** CICs were compared with their appropriate vector + NTshRNA, and DMSO controls. **(G, I)**, *P < 0.05, was considered significant, v6 cDNA, CA-*β*-catenin CDNA overexpressed clonogenic growth **(G)**, and Annexin V positive **(I)** NON-CICs were compared with their appropriate vector controls.

Given that human colorectal CICs can be defined based on high WNT signaling activity (113, 114), we examined if elevated CD44v6 regulated *β*-catenin activation, as seen in **Figures (2C-E)**, can define the FOLFOX resistance of the CIC fraction (as seen in **Figures 2F-G**). To address this, first we generated drug resistant (5-FU, OXA, and FOLFOX resistant) SW480 cells. To generate these drug resistant cells, we first determined IC_50_ values of the parent SW480 cells for 5-Flourouracil (5-FU) and oxaliplatin (OXA) (see **Figure 1B**), because these molecules are the components of FOLFOX. FOLFOX resistance cells were generated by incubating the sensitive parental cells of SW480 (SW480-S) with repeated exposure to increasing concentrations of the drug from 1x FOLFOX (50 µM 5-FU [IC_50_ values for SW480 cells for 5-FU] + 5 µM OXA [IC_50_ values for SW480 cells for 5-FU] + 1 µM leucovorin) to 5 x FOLFOX over 5 days. This exposure and withdrawal cycle was repeated five times for each dose of the drug. The surviving 5-FU resistant (5-FUR), OXA-resistant (OXAR) and FOLFOX-resistant (FR) clones were cultured in normal medium for 5 days and maintained with selection pressure of half the IC_50_ dose of SW480 cells for 5-FU, OXA and FOLFOX. In **Figure 5A**, we compared expression of CD44v6 mRNA (Upper panel, **Figure 5A**) and protein (Lower panel, **Figure 5A**) in sensitive (S), 5-FUR, OXAR, and FR clones with and without 1 x FOLFOX treatment for 8 hours. Results in **Figure 5A** show that FR cells express significantly highest levels (~20 ± 1.67-fold mRNA [Upper panel] and ~8 fold protein [Lower panel]) of CD44v6. OXAR and 5-FUR cells express moderately higher levels (~5-6 ± 0.9 - fold mRNA [Upper panel] and ~3-3.5 fold protein [Lower panel]) compared to sensitive SW480-S cells (**Figure 5A**). Since these resistant cells were selected by repetitive treatment of the sensitive cells with 5 µM OXA, 50 µM 5-FU, and 5 x FOLFOX drugs and maintained under selection pressure of 25 µM of 5-FU, 2.5 µM of OXA and 0.5 x FOLFOX, the basal levels of CD44v6 mRNA and protein in these cells are already elevated, and further addition of 1 x FOLFOX to these cells has little or moderate effect on CD44v6 expression (**Figure 5A**), whereas addition of FOLFOX to sensitive cells increase the CD44v6 mRNA expression to ~ 5 ± 0.49-fold and CD44v6 protein by ~2 fold (Lower panel, **Figure 5A**). These increases in CD44v6 expression in sensitive cells were decreased (data not shown) to basal level at ∼24–48 h due to the depletion of FOLFOX in the media as seen in the results of **Figures 2B, C**. In a further step, we analyzed the expression of CD44v6 mRNA in resistant and sensitive cells with and without treatment with WNT inhibitor LGK974 in FOLFOX treated CICs from resistant and sensitive cells (Inset, **Figure 5A**). The results (Inset, **Figure 5A**) indicate that, WNT3A inhibitor inhibited FOLFOX induced CD44v6 expression substantially in FOLFOX treated CICs from resistant and sensitive cells, suggesting that FOLFOX induced WNT3A maintains CD44v6 mRNA and protein expression in CICs. These data in **Figure 5A**, and in **Figures 2B-C** indicate that FOLFOX treated transient upregulation of WNT3A regulates CD44v6 activation in sensitive cells, and WNT3A induced constitutively active CD44v6 activation in resistant cells to maintain FOLFOX resistance in SW480-FR cells. Abrogation of the CD44v6-WNT3A signaling pathways restores sensitivity to cytotoxic drugs (**Figures 2F, G**).

Second, since cancer cells secrete cytokines to evade drug-induced death (115), and oxaliplatin, a component of FOLFOX, induces a WNT/*β*-catenin target IL-6 (116, 117), we determined whether our resistant cells and sensitive cells differentially secret WNT3A ligands in response to FOLFOX treatment. Results (**Figure 5B**) indicate that SW480-FR cells endogenously produce higher WNT3A levels compared to SW480-S cells, and additional FOLFOX treatment increases WNT3A secretion levels. Additionally, WNT inhibitor LGK974 inhibits the secretion of endogenous WNT3A and FOLFOX induced WNT3A protein in both the cells (**Figure 5B**). This higher WNT3A secretion in response to FOLFOX treatment suggests that WNT3A-induced TOPFlash transactivation may be enriched after FOLFOX treatment. To determine the reporter activity, we overexpressed a luciferase construct containing four native TCF/LEF binding sites (TOPFlash) or its negative-control counterpart (FOPFlash) containing four mutated LEF/TCF binding sites along with a Renilla construct. At 24 hours post-transfection, luciferase activity was measured using the dual-luciferase system. Indeed, the TCF/LEF responsive reporter TOPFlash transactivation increased significantly higher in SW480-FR cells compared to SW480-S cells in response to 1 x FOLFOX treatment for 12 hours (**Figure 5C**), indicating that FOLFOX stimulates WNT3A pathway activation.

Third to determine whether CD44v6-WNT3A signaling has a direct role in mediating FOLFOX resistance, we first investigated whether overexpression of a CD44v6 expression vector in CD44v6 negative PD-FR Non-CICs induces a time-dependent stimulation of WNT3A-mediated transactivation. Results in **Figure 5F** indicate that CD44v6 variant overexpression increases WNT3A-induced TOPFlash luciferase reporter activation. Conversely, we transfected PD-FR/Non-CICs expressing CD44v6 stably with the TCF/LEF responsive reporter TOPFlash and with CD44v6 shRNA. Treatment with WNT3A resulted in activation of WNT3A-induced *β*-catenin signaling, and this was significantly reduced by CD44v6 shRNA1 (**Figure 5G**). The results in **Figures 5F-G** provide evidence that CD44v6 clearly regulates WNT3A-induced *β*-catenin/TCF/LEF transactivation. Next, CD44v6 and *β*-catenin expressions in CICs were knocked down by specific shRNAs, and WNT3A production was inhibited by LGK974 in CICs isolated from SW480-FR cells. In untreated and vector controls, cell viability was not reduced, and cells were resistant to apoptosis upon FOLFOX treatment in SW480-FR/CICs (**Figures 5H, J**). In contrast, Non-CICs show sensitivity to FOLFOX as determined by reduced colony formation in clonogenic growth assays and increased apoptosis determined by apoptosis assay (**Figures 5I, K**). Knockdown of either CD44v6 or *β*-catenin or inhibiting WNT3A production by LGK974, restored FOLFOX sensitivity in SW480-FR/CICs by reversing the resistant phenotype (**Figures 5H, J**), while overexpression of CD44v6 and *β*-catenin in SW480-FR/Non-CICs induced FOLFOX resistance (**Figures 5I, K**). Validations of shRNA of **β*-catenin* were done by the indicated shRNA mediated knockdown and the corresponding shRNA resistant knock-in (KI) (shRNA sequences are in **Table 4** in Methods) gene overexpressions and consequent analysis of indicated proteins in western blots (WB) (**Figure 5D**) following our previously published method (38, 39). Validation of constitutively active (pCA)-*β*-catenin was demonstrated by the indicated protein expressions in WB analysis (**Figure 5E**). The results show: 1) increased resistance of PD-FR/CICs compared to PD-FR/NON-CICs in response to FOLFOX treatment (**Figures 5H** versus 5I); 2) pCA-*β*-catenin and overexpression of v6cDNA mediated increased FOLFOX resistance in SW480-FR/NON-CICs (**Figures 5I, K**); and 3) knockdown of CD44v6 variant, or *β*-catenin, or inhibition of WNT3A by LGK974 nearly eliminated FOLFOX-resistance in SW480-FR CICs (**Figures 5H, J**). These data indicate that CD44v6 regulated WNT3A/*β*-catenin signaling has a vital role for FOLFOX resistance. Indeed, enrichment of CD44v6 expressing CICs from indicated tumor samples after FOLFOX therapy was demonstrated in **Figure 4I**.

**Figure 6:**
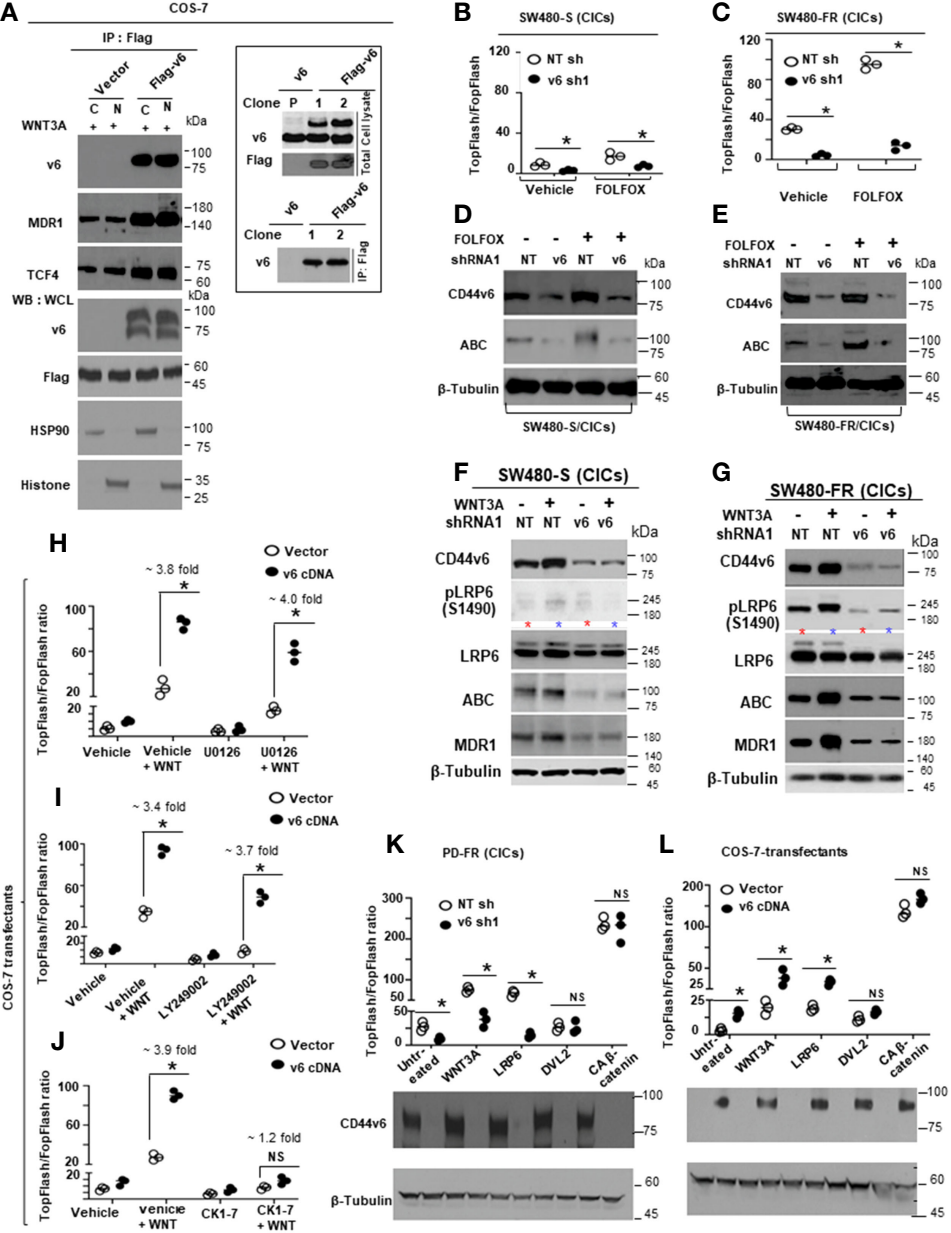
CD44v6 regulated *β*-catenin signaling establishes FOLFOX resistance in CRC-CICs. **(A)**, CD44 negative COS7 cells were stably transfected with vector control or with Flag-CD44v6 cDNA. Nuclear (N) and cytosolic **(C)** fractions were prepared from COS-7/Flag-tagged vector and COS-7/Flag tagged-CD44v6 stable transfectants and immunoprecipitated by the anti-Flag antibody. Flag-immunoprecipitated proteins were analyzed by Western blotting with the indicated antibodies. Upper panel of inset - Western blots of wild-type and Flag-tagged CD44v6 in Flag-CD44v6 knock-in clones and the parental clone (wild type CD44v6 transfectants of COS-7 cells) with either anti-CD44v6 or anti-Flag antibodies, Lower panel of inset - Cell lysates of CD44v6 × Flag knock-in and parental clones were immunoprecipitated with anti-Flag and western blotted with anti-CD44v6. **(B-E)**, CICs from SW480-S cells **(B, D)** and SW480-FR cells **(C, E)** were transfected with NT sh1 or v6 sh1. After 48 hours, CICs were analyzed for FOLFOX stimulated *β*-catenin/TCF4 promoter luciferase activity **(B, C)** as described in **Figure 5C** or, in parallel, to WB analysis with the indicated proteins **(D, E)**. **(F, G)**, CICs from SW480-S **(F)** and SW480-FR **(G)** cells were transfected with NT sh1 or CD44v6 sh1 vectors. After 48 hours, CICs were subjected to WB analysis for the indicated proteins. **(H–J)**, COS-7/vector and COS-7/Flag-CD44v6 stable clones were further transfected with 50 ng TOPFlash and 50 ng TK-Renilla vectors, or with 50 ng FOPFlash and 50 ng TK-Renilla vectors for 48 hours. They were then treated either with vehicle (DMSO) or with the MEK inhibitor U0126 (20 µM) **(H)**, or with the PI3K-Inhibitor Ly294002 (50 µM) **(I)**, or a casein kinase 1 inhibitor CKI-7 (hydrochloride) (CKI-7) (2 µM) **(J)** 2 hours prior to the addition of WNT3A. After 12 hours of induction with WNT3A, cells were lysed and subjected to luciferase measurements. **(K)**, PD-FR CICs were transfected with NT sh1 or v6 sh1. After 48 hours, cells were transfected with TOPFlash and TK-Renilla or with FOPFlash and TK-Renilla vectors. The TOPFlash/FOPFlash promoter was activated by stimulation with WNT3A (20 ng/ml) for 12 hours or by further transfection with LRP6, DVL2 or constitutively active (CA) *β*-catenin for 48 hours. Cells were lysed and subjected to luciferase measurements, and the data are presented as TOPFlash/FOPFlash promoter activity or, in parallel, to WB analysis. **(L)**, COS7-Vector and COS7-CD44v6 clones were transfected with TOPFlash and TK-Renilla vectors, or with FOPFlash and TK-Renilla vectors for 48 hours. The reporter was stimulated with 20 ng/ml WNT3A for 12 hours or by further transfection with LRP6, DVL2, or constitutively active (CA) *β*-catenin for 48 hours. Cells were lysed and subjected to luciferase measurements or, in parallel, to WB analysis. Data are presented as Mean ± SD from n = 3-6 replicates in three independent experiments. All WBs data are representative of 4 independent experiments. **(B, C)**, *P < 0.05 was considered significant, TOPFlash/FOPFlash activity in v6shRNA1 (v6sh1) transfected SW480-S/CICs and SW480-FR/CICs were compared with their NT shRNA (NT sh) transfected cells. **(H–J)**, *P < 0.05 was considered significant, TOPFlash/FOPFlash activity in WNT treated v6 cDNA overexpressed COS-7 cells were compared with untreated v6 cDNA transfectants; TOPFlash/FOPFlash activity in WNT plus inhibitors treated (U0126 [H], LY294002 [I], and CK 1-7 [J]) v6 cDNA overexpressed COS-7 cells were compared with inhibitors only treated v6 cDNA transfectants. **(K, L)**, *P < 0.05 was considered significant, TOPFlash/FOPFlash activity in v6 shRNA1 (v6 sh1) transfectant of PD-FR (CICs) **(K)**, and v6 cDNA overexpressed COS-7 cells **(L)** were compared with the NT shRNA (NT sh) transfected PD-FR (CICs) **(K)**, and vector control transfected COS-7 cells **(L)**.

Fourth, to validate that active *β*-catenin (ABC) and MDR1 protein expressions in CRC cells are correlated with CD44v6 expression, we evaluated the effects of FOLFOX on active *β*-catenin and MDR1 expressions in SW480 cells following FOLFOX treatment with or without CD44v6 shRNA transfection. Knockdown of CD44v6 variant in pCD44v6 overexpressing FOLFOX resistant cells down-regulated ABC and MDR1 expressions and inhibited the v6-containing variants but not the standard or v8 variants (as seen in **Figures 2D, E**). Thus, to define that CD44v6 is a positive regulator of WNT3A/*β*-catenin signaling, we tested whether CD44v6 transcript interacts with the nuclear complex of active *β*-catenin/TCF4 to transcriptionally regulate *MDR1* (118, 119). In an effort to gain a better understanding of the specific functions of the CD44v6 isoform expressed by drug resistant CRC cells, we used CD44-negative COS-7 cells (120). COS-7 cells were transfected with Flag-CD44v6 and control vector (not tagged with flag). In **Figure 6A** inset, we verified the CD44v6 expression and Flag expression in Flag-CD44v6 knock-in clones and in CD44v6 transfected parental COS-7 cells with either anti-CD44v6 or anti-Flag antibodies (upper panel of **Figure 6A** inset). This was further confirmed by immunoprecipitating cell lysates of CD44v6 × Flag knock-in and parental wild type CD44v6 transfectants of COS-7 cells with anti-Flag and subsequently western blotting with anti-CD44v6 (lower panel of **Figure 6A** inset). Co-IPs showed that WNT3A stimulated MDR1 and TCF4 were in a nuclear complex with CD44v6 in CD44v6 overexpressing COS-7 cell (**Figure 6A**, Flag tagged CD44v6 compared to Flag tagged vector transfectant cells).

Since FOLFOX stimulates WNT3A secretion and WNT3A/*β*-catenin transcriptional activity (**Figures 5B, C**), we determined whether knocking down CD44v6 expression would alter FOLFOX-mediated activation of *β*-catenin signaling in CICs isolated from SW480-S and SW480-FR cells. A WNT3A pathway was activated significantly with 1 x FOLFOX treatment for 12 hours in SW480-FR/CICs compared to SW480-S/CICs (as shown in **Figures 6C, E** compared to **Figures 6B, D**). This activation of *β*-catenin-dependent transcription in FR CICs was significantly reduced by knocking down CD44v6 in these CICs (**Figures 6C, E**). Together the results indicate that CD44v6 clearly regulates FOLFOX-induced *β*-catenin activation (hypo phosphorylated, active *β*-catenin [ABC] expression) in SW480-FR CICs compared to SW480-S/CICs (**Figures 6C, E** compared to **Figures 6B, D**).

Fifth, using CICs from sensitive and FR cells of SW480, we specifically tested the involvement of CD44v6 in LRP6 phosphorylation at Serine 1490, an early event in the activation of the WNT signaling (121). Importantly, FOLFOX resistant CICs predominantly express LRP6 phosphorylation at Serine 1490, whereas this phosphorylation was nearly absent in sensitive cells (**Figures 6D, F** compared to **6E, G**). Furthermore shRNA1-mediated knockdown of CD44v6 variant in these CICs strongly inhibited WNT3A-induced phosphorylation of LRP6 (S1490) in SW480-FR CICs (**Figure 6G**). We also observed significant induction of ABC and MDR1 expression in SW480-FR CICs, whereas very little stimulations of endogenous expression of these two proteins by WNT3A was found in SW480-S-CICs, and these inductions are CD44v6 dependent (**Figure 6G** compared with **Figure 6F**). These results indicate that phosphoLRP6 (S1490) distributions, active *β*-catenin (ABC), and MDR1 expression were significantly obstructed in CICs of sensitive cells, whereas LRP6 (S1490) is a positive regulator of LRP6-mediated *β*-catenin (ABC) signaling in SW480-FR CICs (**Figure 6G** compared with **Figure 6F**). Moreover, WNT3A induced a mature glycosylated membrane bound form of LRP6 (upper band of LRP6) that is reduced by knocking down CD44v6 in SW480-FR CICs (**Figure 6G**), whereas in sensitive cells, knocking down CD44v6 represses both the immature endoplasmic reticulum bound (ER) form of LRP6 (faster migrating band) and the mature membrane bound form of LRP6 (slower moving band) (**Figure 6F**). These results provide evidence that WNT3A stimulated CD44v6 expression drives the matured form of LRP6, and that subsequent LRP6 phosphorylation at Serine 1490 activates *β*-catenin signaling and its localization at the membrane in FR-CICs compared to sensitive CICs (**Figure 6G** compared with 6F). Moreover, knocking down CD44v6 variant in SW480-FR CICs reduced both FOLFOX and FOLFOX+WNT3A induced mature glycosylated membrane bound form of LRP6 (122) (upper band of LRP6, red and blue stars) (**Figure 6G**). On the other hand, in sensitive cells, knocking down CD44v6 represses both the immature endoplasmic reticulum bound (ER) form of LRP6 (faster migrating band) and the mature membrane bound form of LRP6 (slower moving band represented by red and blue stars) (**Figure 6F**). These results provide evidence that FOLFOX/WNT3A stimulated CD44v6 drives the matured form of LRP6, and that subsequent LRP6 phosphorylation at Serine 1490 activates *β*-catenin signaling and its localization at the membrane in FR-CICs compared to sensitive CICs (**Figure 6G** compared with 6F). In contrast, WNT3A stimulated CD44v6 drives both mature and immature forms of LRP6 and inactivates *β*-catenin signaling in sensitive SW480-S/CICs (**Figure 6F**). Together, these data demonstrate that CICs have autonomous resistance to FOLFOX therapy that is dependent on CD44v6 expression and CD44v6-dependent WNT3A signaling activation, and on MDR1 expression.

### 3.4 CD44v6-dependent *β*-catenin/TOPFlash transactivation is mediated by the membrane WNT3A and LRP6

CD44v6 regulates multiple receptor tyrosine kinase and non-tyrosine kinase signaling pathways (29–39), and RTKs induce LRP6 phosphorylation/*β*-catenin signaling *via* the mitogen-activated protein kinase (MAPK)/Erk and phosphatidylinositol 3 kinase (PI3K)/Akt signal transduction pathways (123). Moreover, casein kinase 1 (CK1) family members, particularly CK1γ, are known to phosphorylate LRP6 (124). To understand the role of MEK, or PI3K or CK1 in CD44v6 regulated LRP6 phosphorylation, we used their pathway inhibitors and examined whether these pathways affect CD44v6-LRP6/WNT signaling. The results indicate that MEK or PI3K pathways did not impact CD44v6 regulated WNT3A-induced TOPFlash transactivation in a COS-7-CD44v6 stable transfectant clone (**Figures 6H, I**). However, inhibiting CK1 substantially blocked CD44v6 regulated LRP6 phosphorylation in response to WNT3A (**Figure 6J**), indicating that CD44v6 regulated WNT3A/*β*-catenin transcriptional activation in association with CK1. To further confirm that CD44v6 regulated WNT3A-induced TOPFlash transactivation is regulated exclusively by CD44v6 function, we knocked out CD44v6 in SW480-FR CICs and then stimulated them with WNT3A, or co-transfected them with LRP6, or with cytoplasmic protein disheveled 2 (DVL-2), or with CA *β*-catenin overexpressing vectors (**Figure 6K**). Conversely, a CD44v6 gain-of function experiment was done in pCD44v6 overexpressing COS-7 cells, which were then either stimulated with WNT3A, or co-transfected with cDNAs for LRP6, or with DVL-2, or with a CA-*β*-catenin plasmid (**Figure 6L**). **Figures 6K, L** show that TOPFlash promoter activation is decreased by WNT3A and LRP6 treatment when CD44v6 variant has been knocked down (**Figure 6K**), and that they are increased when CD44v6 is increased with transfection using CD44v6 cDNA(**Figure 6L**). In contrast neither the pDVL2 nor the pCA-*β*-catenin treatments alter TOPFlash transactivation. Therefore, TOPFlash promoter activation occurs by membrane constituents WNT3A or LRP6, but not by transfection with cDNAs for cytoplasmic molecules such as DVL2 or CA-*β*-catenin, providing evidence that WNT3A-induced TOPFlash transactivation occurs only in the membrane associated LRP6 activated by CD44v6 presumably in association with CK1 (**Figures 6J, K, L**).

### 3.5 Caveolin-mediated endocytosis is essential for CD44v6-LRP6-*β*-catenin signaling

**Figure 7:**
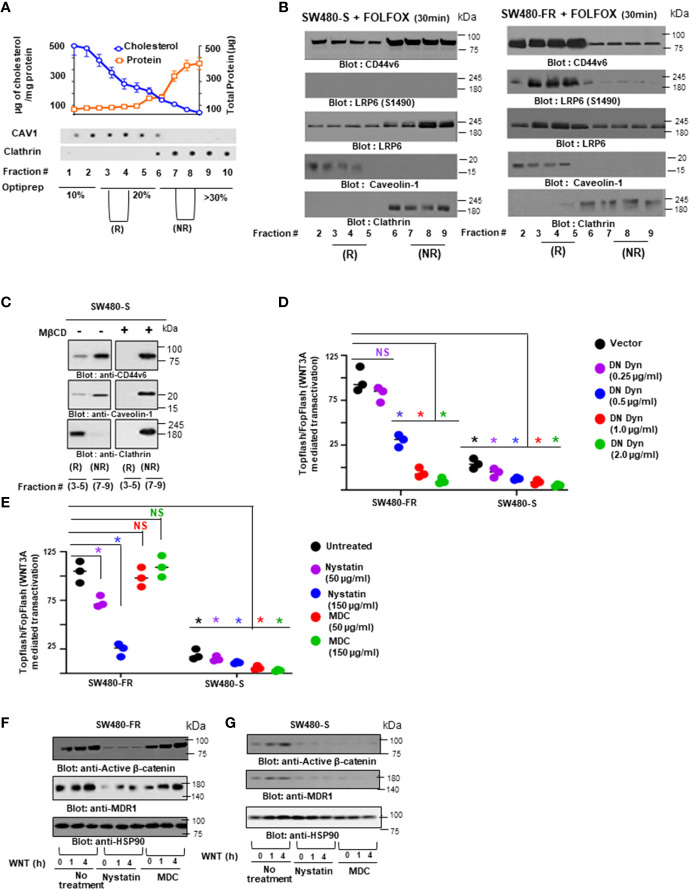
Caveolin-mediated endocytosis is essential for CD44-LRP6-*β*-catenin signaling to maintain FOLFOX resistance. **(A)**, Detergent-resistant membranes, Triton X-100 (1%) insoluble fractions of FR and sensitive cells, were separated in the OptiPrep linear gradients, and distributions of protein and cholesterol across the gradient are shown (details in Methods). Dot Blot analyses show the presence of caviolin1 (CAV1) and clathrin in different Optiprep fractions. **(B)**, SW480-S and SW480-FR cells were treated with 1 x FOLFOX for 30 minutes. The raft (R) < 20% OptiPrep fractions [2-5], and the non-raft (NR) > 20% OptiPrep fractions [6-9] were isolated and analyzed by western blots (WBs) for CD44v6, phosphorylated LRP6 (S1490), LRP6, caveolin-1, and clathrin. **(C)**, SW480-S cells were treated with or without 5 mM methyl-*β*-cyclodextrin (M*β*CD) for 1 hour, and the R and NR fractions were analyzed by WBs for CD44v6 and clathrin. **(D)**, SW480-FR and SW480-S cells transfected with dominant negative dynamin (DN Dyn) [DN K44A] were co-transfected with 50 ng TOPFlash and 50 ng TK-Renilla vectors, or with 50 ng FOPFlash and 50 ng TK-Renilla vectors. After stimulation with WNT3A (20 ng/ml) for 12 hours, cells were lysed and subjected to luciferase measurements. **(E)**, SW480-S and SW480-FR cells were transfected with TOPFlash and TK-Renilla vectors, or with FOPFlash and TK-Renilla vectors luciferase reporter constructs. Transfected cells were treated for 1 hour with the indicated concentrations of nystatin, known to block caveolin-1-mediated endocytosis, or with monodansylcadaverine (MDC), known to block clathrin-mediated endocytosis. After stimulation with WNT3A (20 ng/ml) for 12 hour, cells were lysed and subjected to luciferase measurements. **(F)**, SW480-FR cells and SW480-S cells **(G)** SW480-S cells were treated for 1-4 hours with Nystatin (150 µg/ml) or MDC (150 µg/ml). After stimulation with WNT3A (20 ng/ml) for 1 or 4 hour, cells were lysed and subjected to western blots. Data are presented as Mean ± SD from n = 3-6 replicates in four independent experiments. All WBs data are representative of 4 independent experiments. **(D)**, *P < 0.05 was considered significant, TOPFlash/FOPFlash activity in DN Dyn transfectants of SW489-S, and SW480-FR cells were compared with their vector control transfectants. **(E)**, *P < 0.05 was considered significant, Nystatin and MDC treated SW489-S, and SW480-FR cells were compared with their untreated control cells.

Endocytosis of transmembrane signaling receptors is an important regulatory event in signal transduction including CD44/CD44v6 (33, 76, 125) and WNT/LRP6/*β*-catenin signaling (126, 127). Clathrin-mediated endocytosis has a crucial role in terminating cell survival signaling by inhibiting association of cell surface receptors (128, 129). A CAV1-endocytosis pathway has been shown to function as a platform for receptor mediated signaling by accelerating the sequestering of receptors and signaling molecules within caveolae (130, 131). With evidence that CD44v6 regulates WNT signaling at the level of association with mature LRP6 (as seen in **Figure 6G**), and at the membrane (as seen in **Figures 6K-L**), we hypothesized that formation of intact lipid-rafts at the membrane microdomain may be required for CD44v6 to interact with LRP6. To address this, lighter lipid raft (expressing caveolin-1 [CAV1]) and heavier non raft (Clathrin) membrane fractions were isolated using OptiPrep gradient centrifugation of the Triton X-100-insoluble fractions of the cell lysates that were prepared from SW480-S and SW490-FR cells after 1 x FOLFOX treatment for 30 minutes. Gradient fractions were analyzed for the cholesterol content, protein concentration, and density of the gradient layers after centrifugation. As shown in **Figure 7A**, the low protein content of the 1-5 fractions mostly exhibited high cholesterol and CAV1 expression. The 6-10 fractions with low cholesterol and high protein content exhibit clathrin. To avoid contamination, we used the 3-4 fraction as shown in **Figure 7A**, which is the caveolin-raft fraction between 15%-20% Optiprep gradient layers and depicted as “R”. Similarly, the 7-8 fraction as shown in **Figure 7A** is clathrin-non-raft fractions > than the 30% Optiprep gradient layer and depicted as “NR”. As shown in **Figure 7B**, increased levels of CD44v6 and LRP6 (S1490) localized in R fractions of FR cells, which were greatly reduced in NR fractions expressing clathrin. As shown in **Figure 7B**, transient WNT3A stimulation had little effect on the stimulation of the relatively lower density distribution of CD44v6 and LRP6 to the R fraction in sensitive SW480-S cells compared with the significantly higher density distribution of CD44v6 and LRP6 to the R fraction in SW480-FR cells. Furthermore, phospho-LRP6 (S1490), indicative of activated WNT signaling, co-sediments at higher density with LRP6 and CD44v6 in caveolin containing fractions. Since WNT-mediated phosphorylation of LRP6 at S1490, which is postulated to be required for interaction with and modulation of the *β*-catenin destruction complex (124), is not observed when the receptor is internalized in clathrin containing endocytic vesicles; we can conclude that the increased WNT3A mediated CD44v6-LRP6 (S1490)/*β*-catenin signaling in caveolin-lipid raft is required for maintaining CIC autonomous resistance in FOLFOX-resistant cells (as seen in **Figures 2F-G**, **Figure 4**, and **Figures 5F, H**). Methyl-*β*-cyclodextrin, a cholesterol depleting agent, abolished recruitment of CD44v6 and LRP6 to lipid-rafts (**Figure 7C**).

To confirm the role of endocytosis in FOLFOX induced WNT/*β*-catenin signaling in our FR cells versus sensitive SW480 cells, we transfected these cells with a dominant-negative dynamin (DN) K44A (DN Dyn) that inhibits both clathrin and caveolin-mediated receptor endocytosis (132). After 48 hours, the cells were co-transfected with the TOPFlash reporter and increasing concentrations of DN Dyn. Our FR cells of SW480 that express high levels of CD44v6 and WNT3A (**Figures 5A, B**) show higher TOPFlash promoter activity than sensitive (S) cells of SW480 (**Figure 7D**). Additionally, this increased TOPFlash promoter activity of SW480-FR cells was inhibited by DN Dyn in a dose-dependent way (**Figure 7D**). To further characterize the endocytic pathway mediating WNT3A-induced TOPFlash transactivation in our FR cells compared to our sensitive SW480 cells, we treated these cells with monodansyl-cadaverine (MDC), which blocks clathrin-mediated endocytosis (133), or with nystatin, which disrupts lipid rafts and caveolin dependent endocytosis (132). Results in **Figure 7E** show that Nystatin, but not MDC, inhibits FOLFOX-induced WNT/*β*-catenin TOPFlash promoter activity in FR cells, whereas TOPFlash transactivation was significantly inhibited in SW480-S cells regardless of Nystatin or MDC treatment. In agreement to TOPFlash transactivation blockage by Nystatin, but not by MDC treatment, inhibited active *β*-catenin expression and its target MDR1 expression (**Figure 7F**). However, SW480-S cells show inhibition of the WNT3A-induced active *β*-catenin expression and its target MDR1 expression irrespective of whether the caveolin (Nystatin) or clathrin (MDC) endocytic pathways are modulated (**Figure 7G**). Overall, these results provide evidence that repeated exposure to FOLFOX as it happens in chemo resistant tumors promotes coalescence of CD44v6 and LRP6 induced by LRP6 (S1490) in a SW480-FR/caveolin compartment, whereas in SW480-S cells, brief exposure to FOLFOX fails to associate LRP6 S1490 with a CD44v6-LRP6 complex in a clathrin-microdomain (as seen in **Figure 7B**).

### 3.6 Recruitment of a CD44v6-LRP6 complex toward clathrin-dependent endocytosis in sensitive cells requires DAB2 protein in the complex

**Figure 8:**
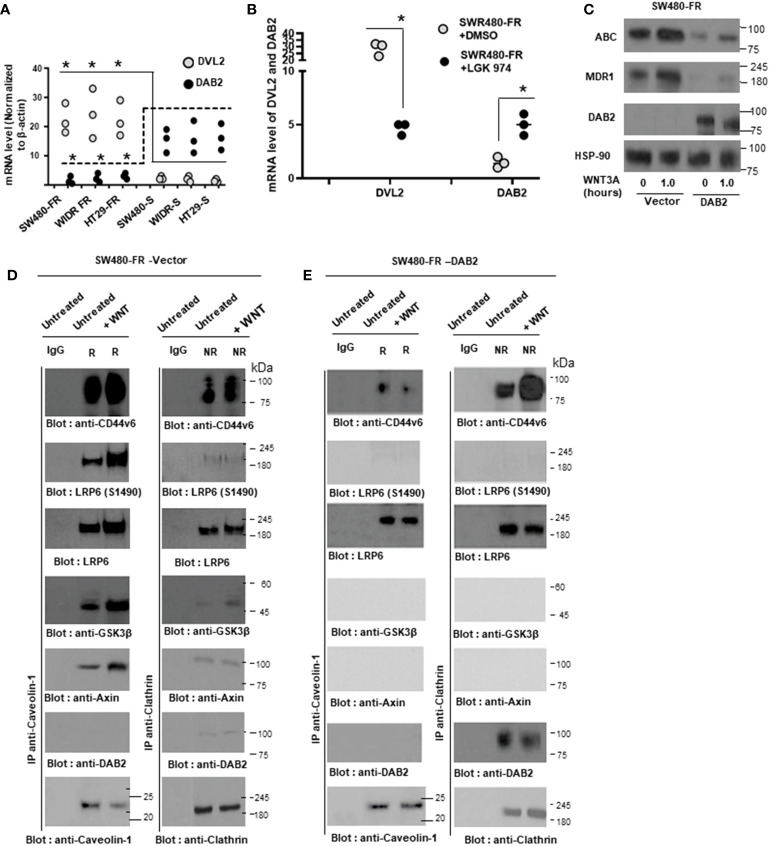
DAB2 favorably sequesters a CD44v6-LRP6 complex in the direction of clathrin-dependent endocytosis to retain FOLFOX sensitivity. **(A)**, mRNA expressions of disheveled protein 2 (DVL-2) and DAB2 protein normalized to *β*-actin in sensitive and resistant pairs of cells are shown by qPCR. **(B)**, Whole cell lysates (WCL) of Vector and DAB2 cDNA transfected SW480-FR cells at time 0 or after 12 hours treatment with or without WNT3A were analyzed by WB for the indicated proteins. Vector **(C)**, and DAB2 cDNA **(D)** transfected SW480-FR cells were treated with or without WNT3A for 12 hours, and the Triton X-100 (1%) insoluble fractions were separated into lipid raft (R) and non-raft (NR) by OptiPrep gradient centrifugation. Pooled OptiPrep gradient fractions (light fractions (3–4) and non-raft heavy fractions (7–8)) were immunoprecipitated with anti-caveolin-1 (left panels) and anti-clathrin (right panels) antibodies. Immunocomplexes were immunoblotted for CD44v6, LRP6 (S1490), LRP6, Axin, GSK3*β*, and DAB2, and for clathrin or caveolin (bottom panels). IgG antiserum was used as negative control for the various immunoprecipitations. QPCR data represent results from 3 independent experiments done in n = 3-6 replicates. All WB data are representative of 4 independent experiments. **(A)**, *P < 0.05 was considered significant, *DVL2* and *Dab2* mRNA expressions in FR cells were compared with S cells. **(B)**, *P < 0.05 was considered significant, *DVL2* and *Dab2* mRNA expressions in LGK974 treated SW480-FR cells were compared with untreated SW480-FR cells.

A recent study links LRP6 to DAB2 in a clathrin microdomain (134). This suggests that LRP6 and CD44v6 distributions to a clathrin domain in sensitive cells (as seen in **Figure 7B**) may be linked to DAB2 in response to brief treatment with FOLFOX or WNT3A. Thus, with evidence that knocking down CD44v6 substantially reduced *β*-catenin/TCF4 promoter TOPFlash activation in sensitive cells compared to the FR counterpart of SW480 cells (as seen in **Figure 6B** versus **Figure 6C**), we hypothesized that DAB2-mediated internalization of LRP6 through the clathrin pathway may be the likely mechanism for stabilization of the destruction complex and the subsequent attenuation of the *β*-catenin signaling activation. To further understand the mechanism of FOLFOX resistance through CD44v6-LRP6-WNT3A/*β*-catenin signaling, the levels of *DVL-2* and *DAB2* gene expressions in our sensitive and FR pairs were determined. As shown in **Figure 8A**, *DVL-2* is highly expressed in SW480-FR cells, whereas SW480-S cells express higher levels of *DAB2*.

Moreover, inhibiting WNT3A by LGK974 substantially suppressed DVL2 with a moderate increase in DAB2 levels (**Figure 8B**). Further, SW480-FR cells are WNT signaling competent as shown by active *β*-catenin (ABC) accumulation and MDR1 induction (**Figures 8B, C**), whereas in SW480-FR cells that ectopically express DAB2 cDNA (SW480/FR-DAB2 clones), WNT3A/*β*-catenin signaling was attenuated (**Figure 8C**).

To further examine this redistribution of LRP6 towards clathrin by DAB2, we fractionated the lipid raft (R, caveolin containing pooled fractions 3-4) and the non-lipid raft (NR, clathrin containing pooled fractions 7-8) fractions in SW480-FR cells that ectopically express vector and DAB2, and determined the relative associations and distributions of -catenin signaling modulators. As shown in **Figures 8D, E**, CD44v6 and phospho-LRP6 (S1490) distributions were substantially impacted by the presence of DAB2. In the absence of DAB2, in vector transfected cells, CD44v6 and LRP6 (S1490) co-immunoprecipitated with the lighter caveolin containing R fractions, and WNT3A stimulation appears to promote this association into a caveolin-compartment. Importantly, phospho-LRP6 (S1490), indicative of activated WNT3A/*β*-catenin signaling, is significantly increased with caveolin containing fractions in SW480-FR-vector clones (**Figure 8D**) and is not present in SW480/FR-DAB2 clones following WNT3A treatment (**Figure 8E**).

Next, we determined the relative associations and distributions of other *β*-catenin modulator proteins. In agreement with the distribution of phospho-LRP6 (S1490), we also found that the presence of axin and GSK3*β* in caveolin-immunoprecipitates depended on WNT3A (**Figures 8C, D**). In contrast, in DAB2 transfected cells, clathrin co-immunoprecipitated with LRP6 and CD44v6 in a WNT3A-dependent manner, but not with axin or GSK3*β*. Collectively, these results provide evidence that DAB2 regulates the localization of a CD44v6-LRP6 receptor complex following WNT3A stimulation in a clathrin compartment, resulting in *β*-catenin destruction with attenuation of interaction with axin and GSK3*β* (**Figures 8C, D**). These results further suggest that LRP6-CD44v6 distributions to a clathrin domain in sensitive cells may be linked to DAB2 in response to brief treatment with WNT3A. In contrast, in FR cells that endogenously secrete WNT3A *via* FOLFOX (as seen in **Figure 5B**), WNT3A-mediated activation of LRP6 phosphorylation at S1490 through CD44v6 (as seen in **Figures 6D-G, 6K, L**) regulated CK1 (**Figure 6J**), which shunts the receptor complex to the caveolin endocytic pathway (as seen in **Figure 7B**).

### 3.7 Palmitoylation and the nuclear localization site of CD44v6 are essential for caveolin1-lipid-raft

#### 3.7.1 Mediated endocytosis to enhance WNT3A-*β*-catenin mediated TCF4 promoter activation

**Figure 9:**
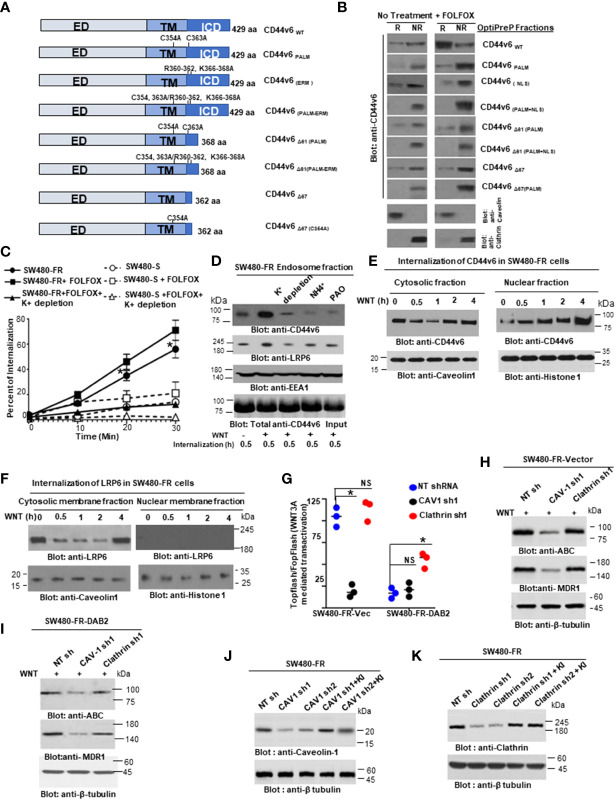
Both palmitoylation and linkage to a nuclear localization site of CD44v6 contribute to recruitment of LRP6 to caveolin1-rafts to regulate CD44v6-induced WNT3A/*β*-catenin signaling. **(A)**, Schematic representations of individual CD44v6 mutants are shown; ED, extracellular domain; TM, transmembrane domain; ICD, intracellular domain. **(B)**, CD44v6 negative SW480-FR/(NON-CICs) were transfected with individual CD44v6 mutants as depicted. Individual CD44v6 cell clones were either untreated (control) or challenged with 1 x FOLFOX for 30 minutes. Raft (R) and non raft (NR) fractions were prepared as described in Methods. **(C)**, SW480-S and SW480-R cells were incubated with biotin conjugated anti-CD44v6 antibody at 4°C separately followed by further incubation at 37°C for 10, 20 and 30 minutes as indicated. The percentage of internalization was measured by flow cytometry after staining with fluorescein conjugated anti-biotin antibody. Data were calculated by setting the mean fluorescence intensity of cells after biotin labeling without glutathione incubation as 100%. **(D)**, SW480-FR cells were cultured in complete media with and without K+ depletion at 37°C for 1 hour followed by further stimulation with WNT3A for 30 minutes. Total cell lysates and endosomes purified by sucrose gradient centrifugation were analyzed by western blotting. **(E-F)**, SW480-FR cells were surface labeled with biotinylating agent (non-cleavable Sulfo-NHS-LC-Biotin). Cells were stimulated with WNT3A at 37°C for the times indicated and placed at 4°C for 1 hour of labelling with the biotinylating agent. Cytosolic membrane and nuclear membrane fractions were affinity purified with avidin-conjugated beads and analyzed by western blotting. **(G-I)**, Sensitive and FR SW480 cells were stably transfected with vector or a DAB2 construct. These stable clones were co-transfected with NTshRNA1, or caveolin1 (CAV1) shRNA1, or clathrin shRNA1. After 48 hours, cells were then transfected with TOP/FOPFlash luciferase reporter constructs prior to 20 ng/ml WNT3A stimulation for 12 hours, and cell lysates were subjected to luciferase activity determination **(G)** and processed for WB analysis for the indicated proteins **(H-I)**. **(J-K)**, Validations of CAV1shRNAs (CAV1 sh1 and CAV1 sh2) and Clathrin shRNAs (Clathrin sh1 and Clathrin sh2) were done by the indicated shRNA mediated knockdown and the corresponding knock-in **(KI)** gene transfections as described in Methods. Target proteins were analyzed by WB analysis (*β*-tubulin, internal control). FACS data in ‘C’ represent are representative of 4 independent experiments. All WBs are representative of 3 independent experiments. QPCR data represent results from 3 independent experiments done in n = 3-6 replicates. **(C)**, *P < 0.05 was considered significant, Internalization of SW480-FR cells was compared with SW480-S cells. Data in ‘G’ represent results from 3 independent experiments performed in triplicates; *P < 0.05 was considered significant, TOPFlash/FOPFlash activity of CAV1 shRNA1 transfectant results were compared with NT shRNA transfectant of SW480-FR-Vector transfectant cells; TOPFlash/FOPFlash activity of clathrin shRNA1 transfectant results were compared with NT shRNA transfectant of SW480-FR-DAB2 cells.

Since *β*-catenin signaling modulators were associated with CD44v6 in lipid-rafts of FR cells (as seen in **Figures 8C, D**), we examined first the mechanism of localization of CD44v6 in lipid-rafts and then the mechanism of recruitment of LRP6 to CD44v6 in response to WNT3A that is secreted in response to FOLFOX (as seen in WNT3A stimulation by FOLFOX in **Figure 5B**). Previous studies identified the inhibitory effects of mutants of CD44 membrane-proximal cysteines, which are palmitoylation sites of CD44 that are essential for the association of CD44 with caveolin lipid-rafts (135, 136). In order to examine whether the CD44v6-palmitoylation sites (present at Cys354 and at Cys363), or the putative CD44v6-nuclear localization sequence (NLS) (^360^RRRCGQKKK^368^) are involved in association of CD44v6 with lipid rafts, we generated a series of the intracellular domain (ICD)-deletion and point mutation mutants from the pCD44v6_429aa_ wild type (pCD44v6-WT) (**Figure 9A**). Next, we expressed these CD44v6 mutant cDNAs as well as pCD44v6-WT constructs in CD44v6 (–)/SW480-FR Non-CICs and explored whether the association of these sites of CD44v6 are involved in caveolin1-lipid raft organization through interaction with cellular *β*-catenin signaling modulator proteins. Data in **Figure 9A, B** indicate that exclusion of the palmitoylation-sites of CD44v6 by changing cysteine at C354 and C363 to alanine (C354A and C363A) did not fully prevent the re-localization of the resultant CD44v6_PALM_ mutants (palmitoylation mutants) into the raft (R) fractions upon FOLFOX treatment for a brief period (30 minutes) in SW480-FR Non-CIC clones expressing these mutants.

Next, we tested whether the ICD of CD44v6 is required for lipid raft affinity through interaction with LRP6 protein. Serial deletion of most of the ICD domain of CD44v6 (CD44v6_Δ67_ [deletion of aa 362 to 429]), and CD44v6 _Δ61C354A, C363A_ (CD44v6_Δ61_ [deletion of aa 368 to 429]) did not block fully the association of the CD44v6 mutants in control and FOLFOX treated SW480-FR/CIC-CD44v6 clones (**Figure 9A, B**). To verify whether CD44v6 is also associated with lipid-rafts *via* the NLS sequence [^360^RRR^362^CGQ^366^KKK^368^]) of CD44v6, a CD44v6_NLS_ Mutant was generated by changing the putative NLS sequence ^360^RRRCGQKKK^368^ to ^360^AAACGQAAA^368^. Because this site is required for cross-linking transmembrane receptors, including CD44v6, to actin-based cytoskeletons (137), the CD44v6-NLS-site (^360^RRR^362^CGQ^366^KKK^368^) is the site where ezrin, radixin, and moesin (ERM) bind (138). Our pCD44v6_Δ67_Mut is devoid of NLS sites of CD44v6. As demonstrated in **Figure 9B**, overexpression of the pCD44v6_Δ67PALM_ Mut and the pCD44v6_Δ61PALM+NLS_ Mut completely block the association of CD44v6 to lipid rafts. Importantly the pCD44v6_NLS_ Mut did not completely block association of this mutant to lipid rafts. Since, the engagement of the pCD44v6_Δ67PALM_ Mut and the pCD44v6_Δ61PALM+NLS_ Mut that were defective in PALM and NLS sites fail to induce lipid raft redistribution/reorganization of CD44v6 (**Figure 9B**), we can conclude that both PALM and NLS sites of CD44v6 are required for CD44v6 to be associated with lipid rafts.

To address the effects of WNT3A on endocytosis directly, first the kinetics of CD44v6 internalization were followed in SW480-FR and SW480-S cells that were incubated with biotin-conjugated anti-CD44v6 antibodies prior to WNT3A treatment for various lengths of time to allow internalization of CD44v6. Cellular intake of biotin-conjugated anti-CD44v6 antibodies increased as a function of time, and a greater intake of CD44v6 was observed with FR cells, suggesting that repeated FOLFOX exposure in SW480-FR cells increases the internalization of CD44v6 compared to that of SW480-S cells (**Figure 9C**). In agreement with this, increasing amounts of CD44v6, as well as of LRP6, were recovered from the early endosome fraction isolated from SW480-FR cells after WNT3A treatment for 30 minutes (**Figure 9D**). Pretreatment of cells with NH4+ or phenyl arsine oxide (PAO), or depletion of K+ to block endocytosis, significantly inhibited the internalization and uptake of CD44v6 and LRP6 (**Figure 9D**). A biotin-labeled endocytosis assay showed that WNT3A stimulation indeed increased the rapid internalization of the biotin-labeled CD44v6 as well as its subsequent nuclear localization (**Figure 9E**). Interestingly, WNT3A stimulation only increased the rapid internalization of the biotin labeled LRP6 but not its nuclear localization (**Figure 9F**). Thus, following triggering by WNT3A, once internalized, endogenous LRP6 and CD44v6 then translocated to membrane-bound vesicles known as early endosomes (**Figure 9F**), where they are sorted, and LRP6 is recycled back to the cell surface within 4 hours of the initial stimulation with WNT3A (**Figure 9F**) presumably for reuse. In contrast, CD44v6 was delivered to the nucleus for further use (**Figure 9E**). Indeed, a previous study has shown that a fragment of CD44 can directly interact with the transcriptional machinery, resulting in the up-regulation of genes containing the TPA (12-*O*-tetradecanoylphorbol 13-acetate) - responsive element, including *CD44* itself (139). However, the mechanism of CD44v6 regulated LRP6 endosomal sorting and of full length-CD44v6 nuclear import/function through its engagement with WNT3A to regulate FOLFOX resistance is not known.

To further confirm whether the increased WNT3A mediated TOPFlash promoter activation can corroborate with the LRP6 (S1490)-CD44v6 complex in a caveolin compartment, we silenced both caveolin and clathrin in SW480-FR-Vec and SW480-FR-DAB2 cells by targeted shRNA for caveolin (CAV1) and clathrin. **Figures 9G-I** show that caveolin knockdown blocks WNT3A/*β*-catenin transcriptional activation, and active *β*-catenin (ABC) and MDR1 expressions in vector transfectant cells, whereas clathrin knockdown overturned the inhibitory effect of DAB2 on WNT3A/*β*-catenin mediated TOPFlash transactivation (**Figure 9G**) as well as on ABC and MDR1 expressions in DAB2 transfected FR cells (**Figures 9H-I**). Validations of CAV1 and clathrin shRNAs are shown in **Figures 9J, K**) following our published methods (38, 39). These results provide evidence that DAB2 segregates a LRP6-CD44v6 complex towards clathrin and away from the interaction of this complex with caveolin to inactivate WNT3A/*β*-catenin signaling regulated by CD44v6 (see **Table 4** for shRNA sequences).

### 3.8 Nuclear translocation of CD44v6 with TCF4 through endosomal sorting contributes to enrichment of TCF4/TOPFlash activation

**Figure 10:**
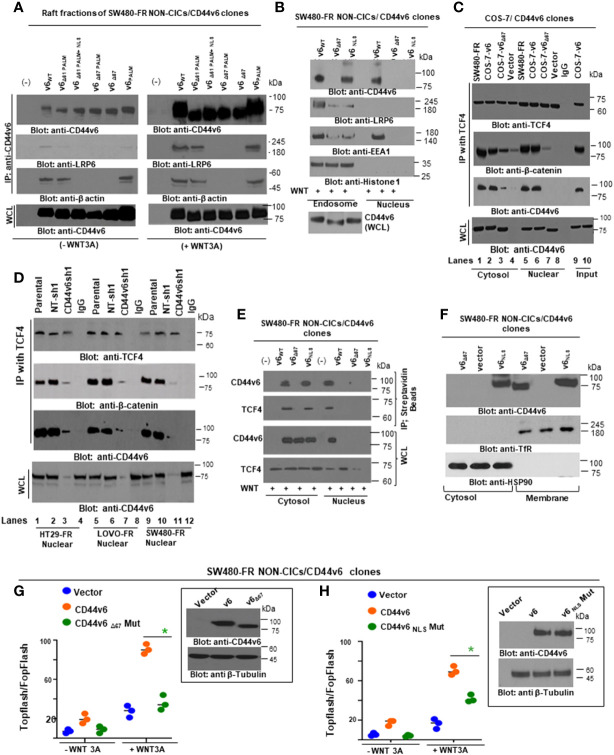
Nuclear localization site (NLS) in the ICD domain of CD44v6 is required for nuclear translocation of CD44v6 through endosomal sorting, and its subsequent association with TCF4 contributes to enrichment of TCF4/TOPFlash transcription. **(A)**, Associations of CD44v6 with LRP6 and actin were examined in pooled lipid raft fractions isolated from SW480-FR NON-CICs/CD44v6 cell clones expressing the indicated CD44v6 mutants (see the structures of CD44v6 mutants in **Figure 9A**). After stimulation with WNT3A for 12 hours, the cell lysates from the individual CD44v6-expressing SW480-FR NON-CIC clones were immunoprecipitated (IP) with an anti-CD44v6 antibody, followed by fractionation and western blotting. **(B)**, WB analyses are shown for endosomal and nuclear fractions in individual SW480-FR NON-CICs/CD44v6 cell clones expressing the v6_Δ67_ mutant (devoid of ICD) and v6 _NLS_ mutants (devoid of nuclear localization site; see **figure 9A**). **(C)**, Nuclear (N) and cytosolic **(C)** fractions were immunoprecipitated with TCF4 or IgG (Control) followed by Western blotting for the CD44v6, *β*-catenin, MDR1 and TCF4 proteins in the SW480-FR cells, and in the COS-7-CD44v6 clones expressing the indicated mutants and vector controls. **(D)**, Nuclear extracts were prepared from the parental HT29-FR, LOVO-FR, and SW480-FR cells, or from cell clones stably harboring lentivirus-encoded NT sh1, or v6 sh1, and they were immunoprecipitated with TCF4 antibody followed by Western blotting with the indicated antibodies. Whole cell lysates (WCL) from the same experiment were used as input and subjected to WB analysis for CD44v6. **(E)**, SW480-FR NON-CICs/CD44v6 cells were incubated with biotin-conjugated CD44v6 at 4°C for 1 hour followed by an additional hour of incubation at 37°C. Cytosolic and nuclear fractions were isolated and immunoprecipitated with streptavidin beads and analyzed by Western Blotting. **(F)**, Lysates from indicated SW480-FR-NON-CIC/CD44v6 cell clones expressing the indicated CD44v6 mutants were subjected to cytosol and membrane fractionation and then analyzed by WBs. The relative purities of the membrane and cytosolic fractions were confirmed by probing for the cytoplasmic protein HSP90 and the membrane protein transferrin receptor (Tf-R). **(G–H)**, SW480-FR-NON-CIC/CD44v6 cell clones expressing the pCD44v6/Δ67mutant **(G)**, and the pCD44v6/NLS mutant **(H)**, were transfected with TOPFlash and control TK-Renilla vectors, or with FOPFlash and TK-Renilla vectors in the presence or absence of 20 ng/ml of WNT3A. After 48 hours, cells were lysed and subjected to luciferase measurements and in parallel to WB analysis. All WBs are representative of 4 independent experiments. All luciferase data represent at least 3 independent experiments done in triplicates. **(G, H)**, *P < 0.05 was considered significant, TOPFlash/FOPFlash activity of WNT3A treated SW480-FR/NON-CICs/CD44v6_Δ67_Mut cells were compared with SW480-FR/NON-CICs/CD44v6 cells, and TOPFlash/FOPFlash activity of WNT3A treated SW480-FR/NON-CICs/CD44v6_NLS_Mutant was compared with SW480-FR/NON-CICs/CD44v6 cells.

With the evidence that FOLFOX stimulates WNT3A secretion (as seen in **Figure 5B**) and that WNT3A enhanced the internalization of the biotin-labeled receptor LRP6 followed by nuclear localization of CD44v6 but not LRP6 (as seen in **Figure 9E, F**), we investigated the mechanism of recruitment of LRP6 by CD44v6 to the lipid rafts. Co-immunoprecipitation assays, as shown in **Figure 10A**, indicate that mutation of the PALM motif in the NLS-deleted-CD44v6_Δ67_ (CD44v6_Δ67PALM_ Mut), or mutation of this NLS motif in CD44v6_Δ61PALM_ (CD44v6_Δ61PALM-NLS_ Mut), disrupted the association of CD44v6 with LRP6 and actin protein in the lipid raft fraction predominantly in WNT3A stimulated cells. In contrast, CD44v6 proteins containing an intact NLS motif in the CD44v6_Δ61PALM_ Mut and the CD44v6_PALM_ Mut, were constantly associated with actin and LRP6, and engagement of CD44v6 strongly enhanced the formation of the CD44v6-LRP6-actin signalosome in lipid-rafts in response to WNT3A stimulation (**Figure 10A**). These results further corroborate that WNT3A induces a CD44v6-LRP6-actin complex in FR cells, and that CD44v6 binds LRP6 through its NLS site (**Figure 10A**). To substantiate that endosomal sorting as well as the NLS site of CD44v6 are essential for the nuclear localization of CD44v6 protein, we tested the subcellularly fractionated endosomal and nuclear fractions in SW480 FR-Non-CIC/CD44v6 transfected clones and found that when the wild-type (WT)-CD44v6 construct was overexpressed in the SW480 FR-Non-CICs, CD44v6 was readily detected in the endosomal and nuclear fractions, whereas LRP6 in these cells was detected only in endosomes (**Figure 10B**). In contrast, CD44v6_Δ67_ failed to internalize. However, the CD44v6_NLS_ mutant internalized efficiently but failed to enter the nucleus, indicating that CD44v6 is internalized through endosomal sorting and imported to the nucleus through the nuclear pore complex (**Figure 10B**). Thus, our data indicate that the FOLFOX induced WNT3A mediated posttranslational modifications of CD44 ((as seen in **Figure 5A**), were required for efficient interaction between CD44v6 clones and LRP6 in membrane and in endosome compartments in SW480-FR-Non CIC/CD44v6 transduced cells (**Figures 10A, B**). Our data also provide evidence that MDR1 and *β*-catenin participate in the formation of the CD44v6-TCF4-*β*-catenin-MDR1 complex in the cytosol and nucleus through its interaction with TCF4 (**Figure 10C**). We also observed that little *β*-catenin was associated with TCF4 in vector transfected COS-7 cells, whereas elevated *β*-catenin was found in the SW480-FR cells and in COS-7-CD44v6_WT_ cells, and that removal of the CD44v6 ICD region (CD44v6_Δ67_) prohibited its interaction with TCF4 and significantly suppressed the association of *β*-catenin with TCF4 (**Figure 10C**). To further confirm that CD44v6 facilitates the association of *β*-catenin with TCF4, we knocked down the expression of endogenous CD44v6 in HT29-FR, LOVO-FR, and SW480-FR cells and showed that the associations of *β*-catenin with TCF4 were substantially precluded (**Figure 10D**). When SW48-FR-Non-CIC/CD44v6 clones were incubated with biotin-labeled CD44v6 in an endocytosis assay, the internalized CD44v6 formed a complex with TCF4 in both the cytosol and in the nucleus, whereas the CD44v6_NLS_ Mutant only formed a complex with TCF4 in the cytoplasm, and CD44_Δ67_ was not internalized (**Figure 10E**). TCF4 translocation to the nucleus was inhibited in cells overexpressing the CD44v6_NLS_ mutant (**Figure 10E**). These data provide evidence that internalized CD44v6 forms a complex with TCF4 in the cytosol, and CD44v6-TCF4 co-translocates to the nucleus in a CD44v6-NLS dependent manner. To test the ability of pCD44v6_Δ67_ and pCD44v6_NLS_ to amplify WNT/*β*-catenin signaling in SW480-FR-NON-CICs, cDNAs were overexpressed for full length pCD44v6-WT, and pCD44v6Δ67 and pCD44v6NLS mutants in SW480-FR-Non-CICs and they were tested for their ability to augment WNT/*β*-catenin signaling in these cells (**Figures 10G, H**). In contrast to full-length pCD44v6-WT, pCD44v6_Δ67_, which is a membrane-localized protein (**Figure 10F**), failed to increase WNT3A-induced TOPFlash activation (**Figure 10G**). Moreover, since the pCD44v6_NLS_ mutant does not allow the CD44v6-TCF4 complex to migrate to the nucleus (**Figure 10E**), the pCD44v6_NLS_ mutant failed to increase WNT/*β*-catenin signaling activation (**Figure 10H**) demonstrating that the effect of CD44v6 on WNT3A/*β*-catenin signaling is mediated through the CD44v6-LRP6 binding, which requires the CD44v6/NLS site (as seen in **Figure 10A**). CD44v6 then sorted in the endosome and translocated to the nucleus with TCF4 (as seen in **Figures 10B, E**) for augmentation of WNT/*β*-catenin signaling in FOLFOX resistant cells (as seen in **Figures 7D, E**).

Thus our results confirm our findings that: 1) CD44v6 regulated LRP6 activation (as seen in **Figures 6F, G**); 2) this activation is at the membrane level (as seen in the **Figures 6K, L**); 3) importantly, FOLFOX stimulated WNT3A induces lipid raft coalescence for CD44v6-LRP6 signaling (**Figures 7B, D, E**); 4) formation of the CD44v6-LRP6 signalosome complex in response to WNT3A stimulation (**Figure 10A**) requires the NLS motif in CD44v6 (**Figure 10B**); 5) the internalized CD44v6 formed a complex with TCF4 in both the cytosol and the nucleus, whereas the pCD44v6_NLS_ mutant only formed a complex with TCF4 in the cytoplasm, and the pCD44v6_Δ67_ mutant was not internalized (**Figure 10E**); and 6) TCF4 was sequestered from the nucleus in cells overexpressing the pCD44v6_NLS_ mutant (**Figure 10E**). These data provide evidence that internalized CD44v6 formed a complex with TCF4 in the cytosol and that CD44v6-TCF4 co-migrated to the nucleus in a CD44v6-dependent manner that depends on a particular CD44v6-NLS site (**Figures 10C-E**) to induce TCF4/FOPFlash promoter activation (**Figures 10G, H**) and subsequent augmentation of drug resistance (as seen in **Figures 2F, G**).

### 3.9 Nuclear CD44v6 stimulates FOLFOX resistance through elevation of efflux of oxaliplatin

**Figure 11:**
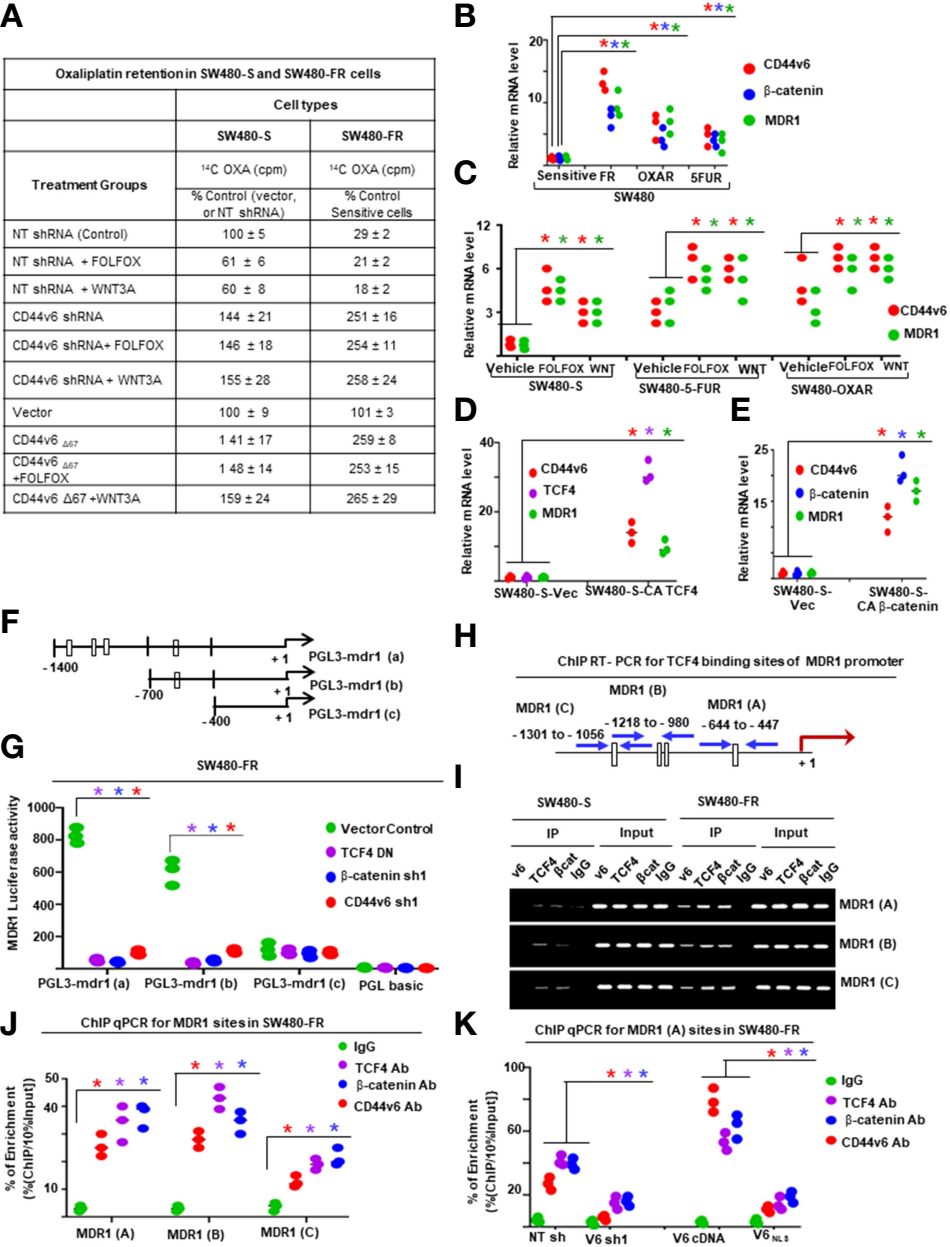
Nuclear CD44v6 associates with TCF4 and functions to modulate MDR1 transcription in FOLFOX resistant cells. **(A)**, The intracellular domain **(ICD)** of CD44v6 induces ^14^C Oxaliplatin Efflux/Retention in SW480-S and SW480-FR cells by FOLFOX and WNT3A treatments. For analyzing drug retention, tumor cells were transfected for 48 hours with CD44v6shRNA, or with a CD44_Δ67_ construct (devoid of the ICD region of CD44v6). They were then treated with ^14^C-oxaliplatin for 24 hours, washed and incubated in drug-free medium alone or with 1 x FOLFOX, or with WNT3A (50 ng/ml) for 12 hours. Cells were harvested and washed, and their numbers were measured by a coulter counter. The radioactivity associated with cells (indicated as intracellular drug retention) were then measured by a liquid scintillation counter as described in Methods. **(B)**, QPCR analyses of CD44v6, *β*-catenin and *MDR1* levels in SW480-S, SW480-FR, SW480-OXAR and SW480-5FUR cells are shown. **(C)**, QPCR analyses of CD44v6 and *MDR1* mRNAs in SW480-S, SW480-5FUR and SW480-OXAR cells treated with or without 1 x FOLFOX or 20 ng/ml WNT3A for 12 hours are shown. **(D-E)**, QPCR analyses are shown for *TCF4*, CD44v6 or *MDR1* levels in SW480-S cells overexpressing constitutively active **(CA)** pTCF4 cDNA **(D)**, or for*β*-catenin, CD44v6 or MDR1 levels in SW480-S cells overexpressing pCA-*β*-catenin **(E)**. **(F-G)**, Transcription activities of the MDR1 promoter with TCF4 binding sites were measured using the indicated pGL3 reporters. **(F)**, The scheme shows the constructs with TCF binding sites in the pGL3 MDR1 promoter. **(G)**, MDR1 Luciferase activity reporter assays are shown for SW480-FR cells overexpressing shRNA for NT (Control), or *β*-catenin, or CD44v6, or a dominant negative pTCF4-DN construct. **(H-I)**, *MDR1* gene expressions regulated by TCF4 in SW480-FR cells are shown. **(H)**, The sketch map shows the predicted TCF4 binding sites (CTTTGA) within the indicated *MDR1* promoter. The transcriptional start site was at +1, and ATG is at the translation start site. The putative TCF4 binding sites (*MDR1* [A], *MDR1* [B] and *MDR*1 [C]) are shown, and their locations are labeled. **(I)**, Semiquantitative PCR products using ChIP PCR primers for *MDR1* **(A–C)** were amplified. **(J)**, ChIP assays were done using anti-CD44v6 (red), anti-TCF4 (purple), anti-*β*-catenin (blue), or irrelevant IgG antibody (green) as negative control using indicated ChIP primers in SW480-FR cells. Total genomic DNA was used as control for the PCR. Quantitative qPCR data representing the qPCR products in immunoprecipitated DNA versus 10% input DNA of ChIP primers for the designated *TCF4* binding sites on *MDR1* [A], *MDR1* [B] and *MDR*1 [C] are shown. **(K)**, ChIP assays were done using either anti-CD44v6 (red), anti-TCF4 (purple), anti *β*-catenin (blue), or irrelevant IgG antibody (green) in SW480-FR cells overexpressing CD44v6 shRNA1, or NT-shRNA1, or with pCD44v6 WT, or pCD44v6_NLS_ mutant constructs. Quantitative ChIP-QPCR data representing the PCR products in immunoprecipitated DNA versus 10% input DNA of ChIP primers for the designated *TCF4* binding sites on *MDR1* **(A, H)** are shown. QPCR and ChIP PCR data represent mean +/- SD, n = 5 replicates from at least 3 independent experiments. **(B)**, *P < 0.05 was considered significant, CD44v6, *β*-catenin, and *MDR1* mRNA levels in 5-FUR, OXAR, and FR SW480 cells were compared with SW480 sensitive cells. **(C)**, *P < 0.05 was considered significant, CD44v6, and *MDR1* mRNA levels of FOLFOX and WNT treated cells were compared with vehicle controls in each cell type.**(D)**, *P < 0.05 was considered significant, CD44v6, TCF4 and *MDR1* mRNA levels of CA-TCF4 transfectant were compared with vector transfectant. **(E)**, *P < 0.05 was considered significant, CD44v6, *β*-catenin, and *MDR1* mRNA levels of CA- *β*-catenin transfectant were compared with vector transfectant. **(G)**, *P < 0.05 was considered significant, CD44v6, *β*-catenin, and *MDR1* mRNA levels of CA- *β*-catenin transfectant were compared with vector transfectant. **(G)**, Luciferase data in “**G”** represent results from 3 independent experiments performed in triplicates. *P < 0.05 was considered significant, Luciferase activity of TCF4 DN. *β*-catenin sh1, and CD44v6 sh1 transfectant of SW480-FR cells for all the PGL3-mdr1 **(A)**, PGL3-mdr1 **(B)**, and PGL3-mdr1 **(C)**, constructs were compared with that of vector control. **(J)**, *P < 0.05 was considered significant, ChIP PCR data for all MDR1 sites **(A–C)** of TCF4. *β*-catenin, and CD44v6 antibody data were compared with that of IgG control in SW480-FR cells.**(K)**, *P < 0.05 was considered significant, ChIP PCR data for MDR1 **(A)** site of TCF4. *β*-catenin, and CD44v6 antibody data were compared with that of IgG control in v6 shRNA1 (v6 sh1) and v6 _NLS_ Mut transfectant of SW480-FR cells were compared with respective controls such as NT shRNA, and v6 cDNA transfectant of cells.

MDRl (P-gp) is known to be involved in the drug efflux and multidrug resistance of solid tumors including CRC (140–142). Recent studies have revealed that both hyaluronan and CD44/CD44v6 stimulate drug resistance by promoting transcriptional up-regulation of the *MDR1* gene and the stimulation of multidrug resistance expression in different cancer types (5, 29, 143, 144). However, the molecular mechanisms underlying the acceleration of *MDR1* gene expression and the stimulation of drug efflux by FOLFOX stimulated WNT/CD44v6 signaling are not well understood. Our data indicate that elevated MDR1 was found in the CD44v6-expressing COS7 cells, and that removal of the CD44v6 ICD (CD44v6_Δ67_) region precluded its interaction with TCF4, and of *β*-catenin with MDR1, which significantly suppressed the association of TCF4 and *β*-catenin (as seen in **Figure 10C**). Moreover, knocking down the expression of endogenous CD44v6 in FR cells prohibited this association of TCF4 with *β*-catenin (as seen in **Figure 10D**) and inhibited cell viability/proliferation (as seen in **Figures 2F, G**). These data suggest that localization of CD44v6 in the nucleus is an important aspect of its FOLFOX resistance function. Thus, we used radioactively labeled ^[14C]^oxaliplatin (OXA) to measure drug effluxes after FOLFOX stimulated WNT3A/*β*-catenin/TCF4/MDR1 signaling in SW480-FR and SW480-S cells. **Figure 11A** shows that the efflux of ^14^C-OXA (a component of FOLFOX) was elevated, leaving low levels of intracellular drug retention after the addition of 1 x FOLFOX, or of 20 ng/ml WNT3A for 2.0 hours. Elevation of efflux of ^14^C-OXA in these cells increases in a time-dependent manner reaching a plateau level 2.0 - 2.5 hours after FOLFOX, or WNT3A treatments (data not shown). Our results clearly show that the efflux of oxaliplatin was elevated in control FR-tumor cells compared to sensitive SW480 cells (**Figure 11A**). This high level of FOLFOX-mediated drug efflux causes low levels of intracellular OXA retention in FR cells compared to sensitive cells (**Figure 11A**). The retention of OXA was further downregulated with FOLFOX or WNT3A treatments (**Figure 11A**). We also observed that knocking down CD44v6, or expressing pCD44v6_Δ67_, or expressing pCD44v6_NLS_ without FOLFOX or WNT3A addition reduced drug efflux, resulting in high levels of intracellular OXA retention (**Figure 11A**). Addition of FOLFOX, or WNT3A to the cells pre-transfected with CD44v6shRNA or with pCD44v6_Δ67_ or with pCD44v6_NLS_ could not reverse the low level of OXA efflux caused by CD44v6shRNA, or pCD44v6_Δ67_, or pCD44v6_NLS_ overexpression (**Figure 11A**). These results clearly indicate that the WNT3A-CD44v6-*β*-catenin/TCF4-CD44v6 interaction has an important role in regulating MDR1-linked drug efflux/retention and multidrug resistance.

### 3.10 Nuclear *β*-catenin/TCF4 associates with CD44v6 to modulate transcription of MDR1 and CD44v6

**Figure 12:**
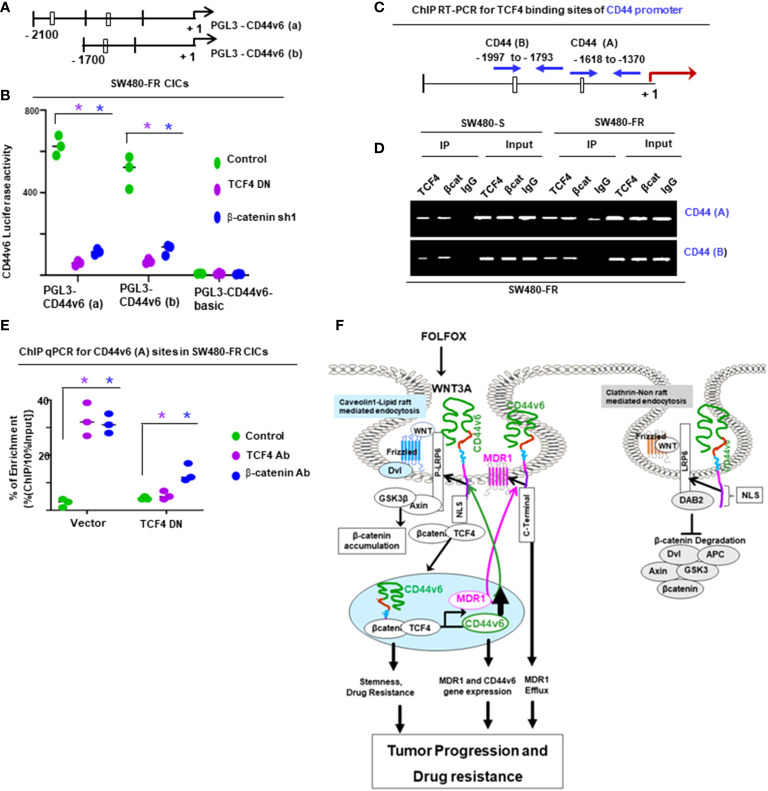
Nuclear TCF4 modulates CD44v6 transcription in resistant cells, and the ICD domain of CD44v6 is required for oxaliplatin (a component of FOLFOX) efflux. **(A-D)**, CD44v6 was transcriptionally regulated by TCF4 in SW480-FR cells. **(A)**, The sketch maps of predicted TCF4 binding sites (CTTTGA) within the CD44v6 luciferase promoter **(A)**, and CD44v6 **(B)** are shown in SW480-FR CICs. **(B)**, CD44v6 luciferase (Luc) activity reporter assays are shown for SW480-FR cells CICs overexpressing dominant negative (DN) TCF4, or *β*-catenin shRNA, or NT-shRNA (control). **(C, D)**. **(C)** The sketch map shows the predicted *TCF4* binding sites (CTTTGA) within the indicated CD44 promoter. The transcriptional start site was at +1, and ATG is at the translation start site. The putative TCF4 binding sites (CD44v6 [A], CD44v6 [B]) are shown, and their locations are labeled by blue arrows. **(D)** Semiquantitative PCR products using ChIP PCR primers for the designated TCF4 binding sites on CD44v6 **(A)** and CD44v6 **(B)**, were amplified in SW480-FR CICs. **(E)**, ChIP-qPCR using PCR primers for designated CD44v6 **(A)** sites (as shown in the schematic diagram in **(C)**) were used for amplification of the CD44v6 mRNA of untreated SW480-FR CICs cells and of CICs overexpressing the indicated vector and TCF4-DN cDNA. **(F)** Proposed model is shown for a positive feedback loop coupling *β*-catenin/TCF4 activation and CD44v6 alternate splicing that sustains cancer initiating cell proliferation and FOLFOX resistance. Left panel: In FOLFOX-resistant cells, in the absence pf DAB2, elevated WNT3A induces CD44v6 that recruits LRP6 to caveolin-micro domain through its nuclear localization site (NLS). The CD44v6-lRP6 complex is internalized through the caveolin-mediated endocytosis followed by endosomal sorting, resulting in accumulation of a TCF4-CD44v6 complex that causes transcriptional activation and the expression of its target genes including CD44v6 and *MDR1* genes. Our results are the first demonstration of a positive feedback loop linking FOLFOX mediated increased WNT3A signaling-dependent alternative splicing of CD44 which is important for cell cycle progression resulting in FOLFOX-resistance in CRC-CICs in the absence of DAB2. Right panel: In sensitive cells in the presence of DAB2, the CD44v6-LRP6 complex is internalized through the clathrin-mediated endocytosis pathway and promotes *β*-catenin destruction and fails to recruit the *β*-catenin/TCF4-CD44v6 complex into the nucleus. Data in B, E represent at least 3 independent experiments performed in triplicates. Values represent means ± SD.; n = 3–5; Semi-quantitative PCR data in “D” are representative of three experiments. **(B)**, *P < 0.05 was considered significant, Luciferase activity of TCF4 DN. and *β*-catenin sh1 transfectant of SW480-FR cells for all the PGL3-CD44v6 **(A)**, and PGL3-CD44v6 **(B)** constructs were compared with that of vector control. **(E)**, *P < 0.05 was considered significant, ChIP PCR data for CD44v6 **(A)** sites of TCF4. and *β*-catenin antibody data were compared with that of IgG control in SW480-FR cells.

As noted above CD44 expression is downstream of the WNT3A/*β*-catenin signaling (30, 88, 89). However, a similar regulation of CD44v6 by WNT/*β*-catenin in response to FOLFOX stimulation has yet to be identified. Our data show that CD44v6-LRP6 is internalized in the presence of FOLFOX-stimulated WNT3A, and after internalization the CD44v6 is translocated to the nucleus by the stimulation of WNT3A. After being internalized and trafficked to the nucleus, the full-length CD44v6 form complexes with TCF4 and MDR1 because CD44v6 and *MDR1* promoters have TCF4 binding sites (see **Figures 11H** and **12C**). To understand the mechanism of this regulation, chromatin immunoprecipitation (ChIP) was done to identify DNA sequences bound by nuclear CD44v6 and *β*-actin complexes. DNA fragments were pulled down by an anti-CD44v6 antibody from a total of 11 clones. A National Center for Biotechnology Information basic local alignment search tool analysis shows that these clones contained sequences corresponding to the promoters of several genes, including *MDR1* (**Table 5**). Among them, 9 clones contained sequences for *TCF4*, and 11 clones contained sequences for *MDR1*. Thus, we tested whether nuclear CD44v6 exerts its transcriptional regulatory function on *MDR1* through interacting with *β*-catenin/TCF4 pathway.

To understand the mechanism of CD44v6-*β*-catenin-MDR1 regulation in CRC, we first determined the mRNA expressions of CD44v6, *β*-catenin and MDR1 in our sensitive and resistant SW480 cells (**Figure 11B**). The results show that resistant SW480-FR cells express these three molecules in significantly increased levels compared to sensitive SW480-S cells. Second, we manipulated the *β*-catenin signaling level through treatment of sensitive and resistant SW480 cells with 1 x FOLFOX or WNT3A and found that these treatments increased CD44v6, *β*-catenin and *MDR1* mRNA expressions in these cells (**Figure 11C**). Importantly, WNT3A had modest effects in sensitive cells compared to resistant cells (**Figure 11C**). Third, we overexpressed cDNAs for TCF4 and *β*-catenin in sensitive SW480-S cells and showed that these strategies increased CD44v6 variant and *MDR1* gene expressions (**Figures 11D, E**).

To further evaluate the relative contribution of TCF4 transcription factor to the regulation of MDR1 promoter activity, transient transfection assays were done using SW480-FR cells with constructs containing TCF binding sites within the *MDR1* promoter cloned into a luciferase reporter plasmid (**Figure 11F**). These constructs were transfected with or without manipulations of CD44v6, TCF4 and *β*-catenin by knocking them down and measuring their luciferase activities using a luciferase assay for the PGL3-mdr1 constructs. The results showed that luciferase activity increases in the presence of TCF binding sites in these cells (**Figure 11G**). Even with only one TCF binding site, pGL3-mdr1(b) construct transfection, a higher luciferase activity was observed compared with a basal promoter lacking TCF binding sites (pGL3-mdr1-c), but less activity than when more than one TCF binding site was present (pGL3-mdr1-a) (**Figure 11G**). The *MDR1* promoter luciferase constructs negatively responded to co-transfection with dominant-negative TCF4-DN cDNA, shRNA1 (sh1) for *β*-catenin and CD44v6. These inhibitory constructs reduced the responsiveness in PGL3-mdr1(a), and PGL3-mdr1(b) in SW480-FR cells (**Figure 11G**). These reductions provide evidence that *TCF* promoter binding and activation of *MDR1* is mediated through both CD44v6 variant and **β*-catenin* in the nucleus.

We used ChIP assays to understand the interaction of CD44v6 and *β*-catenin proteins at TCF binding regions of the MDR1 promoter and of their bound chromatin from the protein mixture that was extracted from SW480-FR CICs. ChIP assays were done and immunoprecipitated, and the input DNAs were amplified using primers (see **Table 6**) covering the indicated *TCF4* binding sites as shown in **Figure 11H**. ChIP assays (**Figure 11I**) showed that *β*-catenin/TCF4 bound to three *MDR1* sites in both sensitive and resistant SW480 cells. CD44v6 only bound to these three sites in resistant cells and was associated with markedly increased binding of TCF4 and *β*-catenin when compared to sensitive cells (**Figure 11I**). ChIP analyses provided direct evidence for the ability of TCF4 and *β*-catenin to bind to the promoters of CD44v6 (**Figure 11J**). Knockdown of CD44v6, or blocking the NLS site of CD44v6 using the pCD44v6_NLS_ mutant, showed reduced endogenous *MDR1* promoter binding in SW480-FR cells (**Figure 11K**). This validates our results from the luciferase reporter assay that CD44v6 and TCF4/*β*-catenin co-regulate *MDR1* expression in a CD44v6-regulated manner in FOLFOX resistant cells, and this CD44v6-regulated *MDR1* gene expression through *TCF4* sites requires the CD44v6 NLS site.

Several putative TCF binding sites were located 2 kilobases upstream of the transcriptional start site of the *CD44* gene (**Figure 12A**). A fragment of the *CD44* promoter (-2100 to 500 bp) was fused upstream of the firefly luciferase gene in pGL3-CD44v6 (a), and similarly pGL3-CD44v6 (b) was prepared (-1700 to 500 bp). Both pGL3-CD44v6 (a) and pGL3-CD44v6 (b) contain *TCF4* binding sites. Luciferase assays were used to directly examine the interaction between *β*-catenin/*TCF4* and the *CD44* promoter in SW480-FR CICs. The luciferase activities in SW480-FR cells transfected with dominant negative TCF4-DN and shRNA1 (sh1) for *β*-catenin were significantly lower than in the vector group (**Figure 12B**), while *β*-catenin and TCF4 overexpression significantly increased the luciferase activity (data not shown). This provides evidence that *β*-catenin/TCF4 increases CD44v6 transcription activity. To identify whether *β*-catenin can bind to TCF4 binding sites in the *CD44* promoter in SW480-FR cells CICs, ChIP assays were done and immunoprecipitated, and input DNAs were amplified using primers (see **Table 6**) covering the indicated TCF4 binding sites of the *CD44* promoter as shown in **Figure 12C**. To validate these results, conventional ChIP analyses were done, and they provided direct evidence for the ability of TCF4 to bind to the *CD44* promoter in SW480-FR CICs (**Figures 12D, E**). As noted in **Figures 5B, C**, FOLFOX induces secretion of WNT3A and WNT3A/*β*-catenin transactivation in CICs. Further, **Figures 5** and **6** show that FOLFOX mediates CD44v6 expression to regulate WNT3A/*β*-catenin TCF4 signaling, and in this section, we showed that a *β*-catenin/TCF4 pathway promotes both *CD44* and *MDR1* gene expressions in FR-CICs (**Figures 11** and **12**).

Overall, this study indicates that FOLFOX treatment induces both WNT3A and CD44v6 through its NLS site, recruits LRP6 to CAV1-rafts, and activates LRP6 (S1490) to promote WNT3A/*β*-catenin/TCF4 signaling that induces CD44v6 expression (**Figures 5**-**12**). This functions through a positive feed-back loop between CD44v6 and FOLFOX induced WNT3A/*β*-catenin/TCF4 activation stimulates *MDR1* gene expression and CD44v6 splicing that sustains FOLFOX resistance. Furthermore, we have found that the failure to recruit MDR1 into a complex with CD44v6 using overexpression of pCD44v6Δ67 or silencing CD44v6 variants abolishes FOLFOX-induced active multidrug efflux and increases drug retention.

## 4 Discussion

5-FU, a component of FOLFOX, promotes CD44v6 (6), and induces stemness/self-renewal in CRC activation by WNT/*β*-catenin signaling (145), and CICs are more resistant to therapy. Thus, it is not difficult to understand how the induction of CD44v6 and WNT/*β*-catenin signaling in human colorectal CICs directly affects the treatment outcome. WNT/*β*-catenin signaling is one of the key cascades regulating development and stemness, and has also been tightly associated with CICs in the gut and with promoting self-renewal of CRC-CICs (19, 146–148). Our data indicate strong support that WNT signaling and CD44v6-containing variant expression might be coordinately controlled by a positive feedback loop in CICs isolated from FOLFOX resistant colorectal tumor. This is in accordance with our findings that feedback regulation is a key aspect of CD44v6 signaling in which WNT/*β*-catenin signaling promotes CD44v6 splicing, and CD44v6 then sustains WNT/*β*-catenin signaling, which is important for cell sycle progression and uncontrolled drug resistance in CRC-CICs (**Figure 2**). In line with this, negative feedback mechanisms are likely necessary in normal colon cells to regulate uncontrolled WNT/*β*-catenin/CD44v6 activation. Further, targeting WNT or CD44v6 showed increased FOLFOX sensitivity and completely blocked the WNT3A mediated transactivation of the CICs cell cycle profile and drug resistance (as seen in **Figures 2F, G, H**, **Supplemental Figure 1D**, **2I** and **5G**). This indicates that the CIC’s FOLFOX resistance was generated by WNT/*β*-catenin *via* CD44v6.

In our recent study, the importance of CD44v6-YB-1-MDR1 signaling that maintains chemo resistance in CICs was described (5). However, upstream signaling mechanisms leading to drug resistance with potential crosstalk between CD44v6 signaling and WNT-receptor LRP6 involvement in FOLFOX-therapy in CRC are largely unknown. To explore the link between chronic FOLFOX-therapy stress and colorectal CIC “stemness”, we initiated a comprehensive molecular and functional analysis of CD44v6 regulation of WNT/*β*-catenin signaling and its effects in CICs isolated from FOLFOX resistant human tumor specimens, and from SQ tumor samples. Since the expression of CIC markers may not be regulated in a coordinated fashion, we analyzed three widely used stem-cell/progenitor markers (EpCAM, ALDH1 and CD133) in CD44v6 (+) FACS sorted cells to isolate CICs and used them in several cellular/molecular functional tests. These included WNT/*β*-catenin/TCF4 mediated TOPFlash promoter activity, lipid raft localization assays, internalization/endosomal sorting and nuclear trafficking analysis, cell viability, Annexin V positive cells expressing cell apoptosis, tumor sphere formation, xenograft tumor growth, and MDR1 and CD44v6 transcriptions through a nuclear CD44v6-TCF4 complex.

We investigated drug resistance and consequent tumor relapse as a mechanism to mediate self-renewal functions of CICs (149) and evaluated the tumorigenic potential of freshly isolated CD44v6 (+) CICs and CD44v6 (–) populations (Non-CICs), and of unsorted bulk cells to form colon tumors by implantation of these cells into immunocompromised mice. In this xenograft model of CRC, implantation of 5 x 10^5^ Non-CICs from colorectal tumor cells did not induce tumor formation (**Figures 4F-G**). Even though a higher number of CD44v6 (+) cells was present in 5 x 10^5^ unfractionated bulk tumor cells, tumor formation capacity of as few as 2 x 10^3^ CD44v6 (+) CICs was faster and more efficient than tumor formation obtained with the unfractionated bulk tumor cells (**Figures 4F-4G**). To evaluate whether CD44v6 (+) CICs can reproduce long-term tumorigenic potential in progressive recipients, we analyzed their ability to generate tumor sphere formation after serial transplantations in secondary and tertiary mouse recipients. During the *in vivo* passaging, CD44v6 (+) CICs did not lose their tumorigenic potential but instead increased their faster tumor size and growth (**Figure 4G**). Data in **Figure 4H** show that only CD44v6 (+) CICs form tumor spheres in primary, second and third generations of mice whereas tumorigenic potential of CD44v6-Non-CICs was completely lost in secondary and tertiary recipients of xenografts. Thus, the CD44v6 (+) CICs are confined to a small cell population resident in the colon tumor and have the ability to reproduce long-term tumorigenic potential in serial recipient xenografts with unlimited tumorigenic potential, whereas CD44v6 (–)Non-CICs include transient amplifying of differentiated cells.

Gain-in-function and loss-in-function of CD44v6 experiments (**Figures 6K, L**) demonstrated that CD44v6 acts at the level of WNT3A and LRP6 and upstream of DVL-2 and *β*-catenin. Nonetheless DVL-2 was shown to be required for LRP6 phosphorylation (150). These data are further supported by the finding that CD44v6 regulates WNT3A-dependent LRP6 phosphorylation at the level of CIC membranes (as seen in **Figures 6F, G, K, L**). These data and the findings show that either clathrin-mediated (Non-lipid raft) or caveolin-mediated (lipid-raft) internalization of LRP6 is key for the WNT/*β*-catenin signaling. This led us to study the interaction of DAB2 with CD44v6 and LRP6 in Non-lipid raft or lipid-raft micro-domains that regulate WNT3A/*β*-catenin signaling. Additionally, we provide evidence that WNT3A-mediated interaction of CD44v6 with LRP6 phosphorylation (LRP6 [S1490]) by CD44v6 is required for its association with DVL-2 and caveolin in lipid rafts, whereas DAB2 attenuates the WNT signaling by shifting the CD44v6-LRP6 complex to clathrin mediated endocytosis in sensitive cells (**Figures 7**, **8**). In the absence of DAB2, WNT induces internalization of CD44v6, which in turn interacts with LRP6 through caveolin mediated endocytosis (**Figure 7B**) and promotes membrane localization of matured LRP6. This results in WNT/*β*-catenin signaling in a CD44v6 mediated CK2-dependent manner but not through CD44v6 regulated (MAPK)/Erk and phosphatidylinositol 3 kinase (PI3K)/Akt pathways (**Figure 6**), whereas in its presence DAB2 binds CD44v6 and LRP6 in a WNT-dependent manner, and pushes LRP6 towards clathrin mediated endocytosis and suppresses *β*-catenin signaling and MDR1 expression (**Figure 8**). We propose, therefore, that FOLFOX regulates cellular DVL-2 and DAB2 levels that modulate CD44v6-LRP6 interaction and consequent WNT/*β*-catenin signaling by regulating endocytosis of LRP6 and CD44v6.

In a further step, we have presented new evidence indicating that FOLFOX induces WNT signaling through CD44v6 mediated recruitment of LRP6/LRP6 (S1490) to caveolin-dependent internalization of these receptors (**Figure 7**, **Figures 9C-F**). The internalized CD44v6 and LRP6 were sorted in endosomes, and CD44v6 formed a complex with TCF4 in both the cytosol and nucleus. Then the CD44v6 with TCF4 co-translocated to the nucleus in a CD44v6-dependent manner, and this association requires the nuclear localization signal (NLS) of CD44v6 (**Figures 10B-E**). The NLS motif that mediates CD44v6 nuclear translocation was mapped to the intracellular domain of CD44v6 (**Figure 9A**). Importantly, internalized CD44v6 forms a complex with TCF4 and *β*-catenin, and this complex is translocated to the nucleus through a CD44v6 dependent manner (**Figure 10C**). Expression of a CD44 (NLS) mutant sequesters TCF4 in the cytosol (**Figure 10E**). In the nucleus, the TCF4 remains associated with CD44v6 and binds to the *TCF4, MDR1* and *CD44* promoters, leading to increased *MDR1* activity and drug efflux (**Figures 11**, **12**). Further our data provide evidence that WNT3A-mediated phosphorylation of S1490 of LRP6 mediated by CD44v6 is required for its association with DVL-2 and caveolin, whereas DAB2 attenuates the WNT signaling by shifting the CD44v6-LRP6 complex to clathrin mediated endocytosis in sensitive cells (**Figures 8** and **9G-I**). Consequently, all these events contribute to CD44v6-WNT3A-mediated therapeutic drug resistance in CICs of colon tumor cells. This provides evidence that targeting the CD44v6-WNT3A mediated *β*-catenin/TCF4-MDR1 signaling pathways and the increased MDR1 efflux function may represent a novel approach to overcome chemotherapy resistance in colon tumor CICs.

## 5 Conclusion

### 5.1 Proposed model for a positive feedback loop that couples *β*-catenin/TCF4 activation and CD44v6 alternate splicing that sustains CIC drug resistance

We propose that FOLFOX mediated WNT3A stimulation of CD44v6 through its NLS site recruits LRP6 in a caveolin-microdomain in the absence of DAB2. The CD44v6-LRP6(S1490)/LRP6-signalosome is internalized through endosomal sorting resulting in nuclear accumulation of a *β*-catenin/TCF4-CD44v6 complex, which then transcriptionally activates stemness-associated *MDR1* and a CD44v6-containing isoform, which sustains drug-resistance in CRC-CICs. In contrast, a CD44v6-LRP6 complex is internalized through the clathrin microdomain in sensitive cells and fails to recruit the *β*-catenin/TCF4-CD44v6 complex in the nucleus. Our data, suggest a biphasic activation of *β*-catenin in response to either WNT3A or FOLFOX (**Figure 2I**). Furthermore, upon WNT3A stimulation either alone or through FOLFOX (**Figure 5B**) produces an early surge of nuclear *β*-catenin activation independent of CD44v6 variants. This signal is rapidly down regulated by CD44v6 shRNA when FOLFOX or WNT3A generates CD44v6 expression after 2 hour of either WNT3A, or of FOLFOX stimulation (**Figure 2I**). However WNT3A/*β*-catenin activation signal initiates a positive feedback loop by inducing CD44v6 expression through alternative splicing (**Figure 12F**).

## Data availability statement

The datasets presented in this study can be found in online repositories. The names of the repository/repositories and accession number(s) can be found below: https://www.ncbi.nlm.nih.gov/, (http://portals.broadinstitute.org/gpp/public/).

## Ethics statement

The studies involving human participants were reviewed and approved by Medical University of South Carolina. The patients/participants provided their written informed consent to participate in this study. The animal study was reviewed and approved by IACUC, all animal care and experiments were in accordance with the institutional guidelines of IACUC-2017-00250 (approval date: 2019/03/14- 2021/03/29).

## Author contributions

The paper was written by SM and SG. SG and SM designed the study, physically worked together at any time and made an equal contribution to the conception of every claim of the work involved for the study. Being an expert for his work on hyaluronic acid and CD44 research, VH analyzed the effect of CD44v6shRNA on *β*-catenin/TCF4 signaling. VH reviewed, edited the multiple versions of the drafts and final versions of the text, figures, figure legends, participated in designing experiments and supplied reagents. RM participated in conception of the idea in validating the CICs and CAFs that were used in this study. RM edited the drafts, provided reagents, and participated in designing experiments. NK, further discussed with SM and SG to validating the specificity of the shRNAs. NK helped in editing the drafts and participated in the experimental design. We thank Christopher Koivisto from Hollings Cancer Center for sharing his views for this manuscript. All authors contributed to the article and approved the submitted version.

## Funding

This work was supported by the: 1) 1R03CA167722-01A1 (to SM and SG); 2) 1K12HL141952-02 (to VH); 3) 2P20GM10399; NIH IDeA Network for SC Biomedical Research Excellence, 40 2 P30 GM131959-01, 5) 19TPA34900016 (to RM), and 7) SCTR grant (UL1 TR001450 for SM).

## Conflict of interest

The authors declare that the research was conducted in the absence of any commercial or financial relationships that could be construed as a potential conflict of interest.

## Publisher’s note

All claims expressed in this article are solely those of the authors and do not necessarily represent those of their affiliated organizations, or those of the publisher, the editors and the reviewers. Any product that may be evaluated in this article, or claim that may be made by its manufacturer, is not guaranteed or endorsed by the publisher.

## Supplementary material

Supplementary Figure 1
**(A, B)**, Validations of CD44v6 shRNAs (v6 sh1 and v6 sh2) and WNT3A shRNAs (WNT3A sh1 and WNT3A sh2) used in panels **(D–G)** were done by the indicated shRNA mediated knockdown and the corresponding knock-in (KI) gene transfections in SW480-FR cells as described in Methods. Target proteins were analyzed by WB analysis (*β*-tubulin, internal control). **(C)**, The effect of shRNA-mediated knockdown of CD44v6 in SW480-FR cells on the expression of CD44v6 mRNA was determined by real-time PCR (at 24 h; RQ, relative quantification). Validation of expression vector CD44v6 cDNA (v6 cDNA) was done by WB analysis (*β*-tubulin, internal control) **(D)**, Bar graph summarizing the flow cytometry cell cycle profile analysis after WNT3A stimulation in G2.M-arrested-SW480-cells which were previously transfected with NT shRNA or v6 shRNA for 24 hours. These cells were collected at indicated times after WNT3A -stimulation and cell cycle were analyzed (details in Method section). Data are presented as Mean ± SD from n = 3 replicates in three independent experiments. FACs data are representative of three experiments *P < 0.05, **P < 0.01 were considered significant, percent cells in S phase in CD44v6 shRNA transfected cells compared with NT shRNA transfected cell.

## Abbreviations

CD44s, Standard isoform of CD44 having no variant exons; CD44v, CD44 splice variant; CD44v6, variant 6 of CD44; CIC, cancer initiating cell; CRC, colorectal cancer; 5-FU,5-fluorouracil; OXA, oxaliplatin; FOLFOX, 5-FU plus OXA plus leucovorin; ALDH1, aldehyde dehydrogenase; LRP6, low-density lipoprotein receptor-related protein; TCF, T-cell factor; LEF, lymphoid enhancer factor; Dkk1, Dickkopf 1; DAB2, endocytic adaptor disabled-2; DVL, Disheveled family *of* proteins in mammals; Tf, transferrin; Nano (Tf-PEG-PEI nanoparticles); MDR1, multidrug resistance protein 1; IP, immunoprecipitation; WB, Western Blotting; DN, dominant negative; sh, shRNA; SQ, sub cutaneous; R, lipid-raft fraction; NR, non lipid-raft fraction.
