# Supplementary material for: Chemotherapy induces feedback up-regulation of CD44v6 in colorectal cancer initiating cells through β-catenin/MDR1 signaling to sustain chemoresistance
Source: Front Oncol. 2022 Oct 18;12:906260. doi: 10.3389/fonc.2022.906260 (PMC9623568; doi:10.3389/fonc.2022.906260)
Supplement: Supplementary Figure 1 — (A, B), Validations of CD44v6 shRNAs (v6 sh1 and v6 sh2) and WNT3A shRNAs (WNT3A sh1 and WNT3A sh2) used in panels (D–G) were done by the indicated shRNA mediated knockdown and the corresponding knock-in (KI) gene transfections in SW480-FR cells as described in Methods. Target proteins were analyzed by WB analysis (β-tubulin, internal control). (C), The effect of shRNA-mediated knockdown of CD44v6 in SW480-FR cells on the expression of CD44v6 mRNA was determined by real-time PCR (at 24 h; RQ, relative quantification). Validation of expression vector CD44v6 cDNA (v6 cDNA) was done by WB analysis (β-tubulin, internal control) (D), Bar graph summarizing the flow cytometry cell cycle profile analysis after WNT3A stimulation in G2.M-arrested-SW480-cells which were previously transfected with NT shRNA or v6 shRNA for 24 hours. These cells were collected at indicated times after WNT3A -stimulation and cell cycle were analyzed (details in Method section). Data are presented as Mean ± SD from n = 3 replicates in three independent experiments. FACs data are representative of three experiments *P < 0.05, **P < 0.01 were considered significant, percent cells in S phase in CD44v6 shRNA transfected cells compared with NT shRNA transfected cell. [file Presentation_1.pptx]

## Slide 1
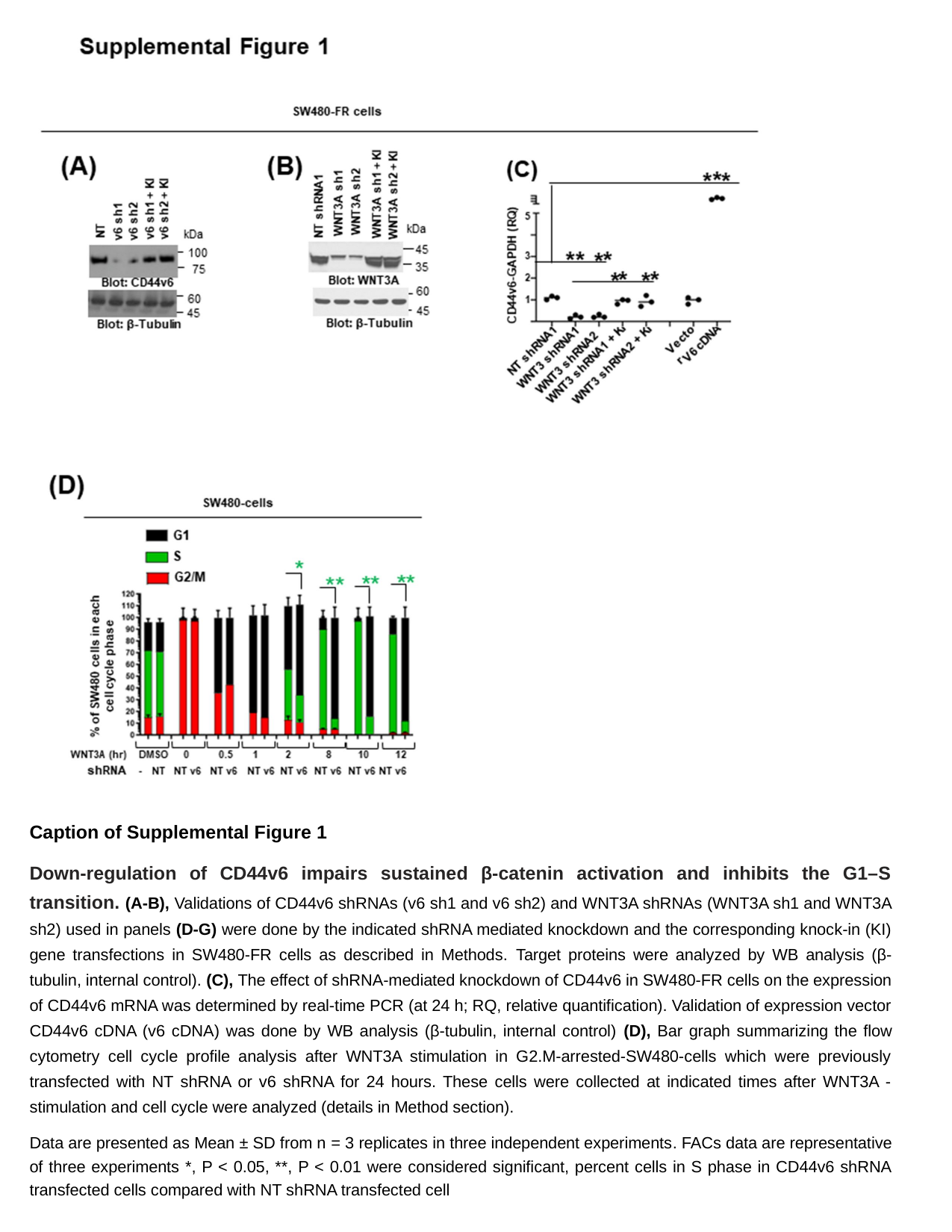

Caption of Supplemental Figure 1
Down-regulation of CD44v6 impairs sustained β-catenin activation and inhibits the G1–S transition. (A-B), Validations of CD44v6 shRNAs (v6 sh1 and v6 sh2) and WNT3A shRNAs (WNT3A sh1 and WNT3A sh2) used in panels (D-G) were done by the indicated shRNA mediated knockdown and the corresponding knock-in (KI) gene transfections in SW480-FR cells as described in Methods. Target proteins were analyzed by WB analysis (β-tubulin, internal control). (C), The effect of shRNA-mediated knockdown of CD44v6 in SW480-FR cells on the expression of CD44v6 mRNA was determined by real-time PCR (at 24 h; RQ, relative quantification). Validation of expression vector CD44v6 cDNA (v6 cDNA) was done by WB analysis (β-tubulin, internal control) (D), Bar graph summarizing the flow cytometry cell cycle profile analysis after WNT3A stimulation in G2.M-arrested-SW480-cells which were previously transfected with NT shRNA or v6 shRNA for 24 hours. These cells were collected at indicated times after WNT3A -stimulation and cell cycle were analyzed (details in Method section).
Data are presented as Mean ± SD from n = 3 replicates in three independent experiments. FACs data are representative of three experiments *, P < 0.05, **, P < 0.01 were considered significant, percent cells in S phase in CD44v6 shRNA transfected cells compared with NT shRNA transfected cell
